# Supplementary figures and images for: In-silico tool based on Boolean networks and meshless simulations for prediction of reaction and transport mechanisms in the systemic administration of chemotherapeutic drugs
Source: PLoS One. 2025 Feb 7;20(2):e0315194. doi: 10.1371/journal.pone.0315194 (PMC11805580; doi:10.1371/journal.pone.0315194)

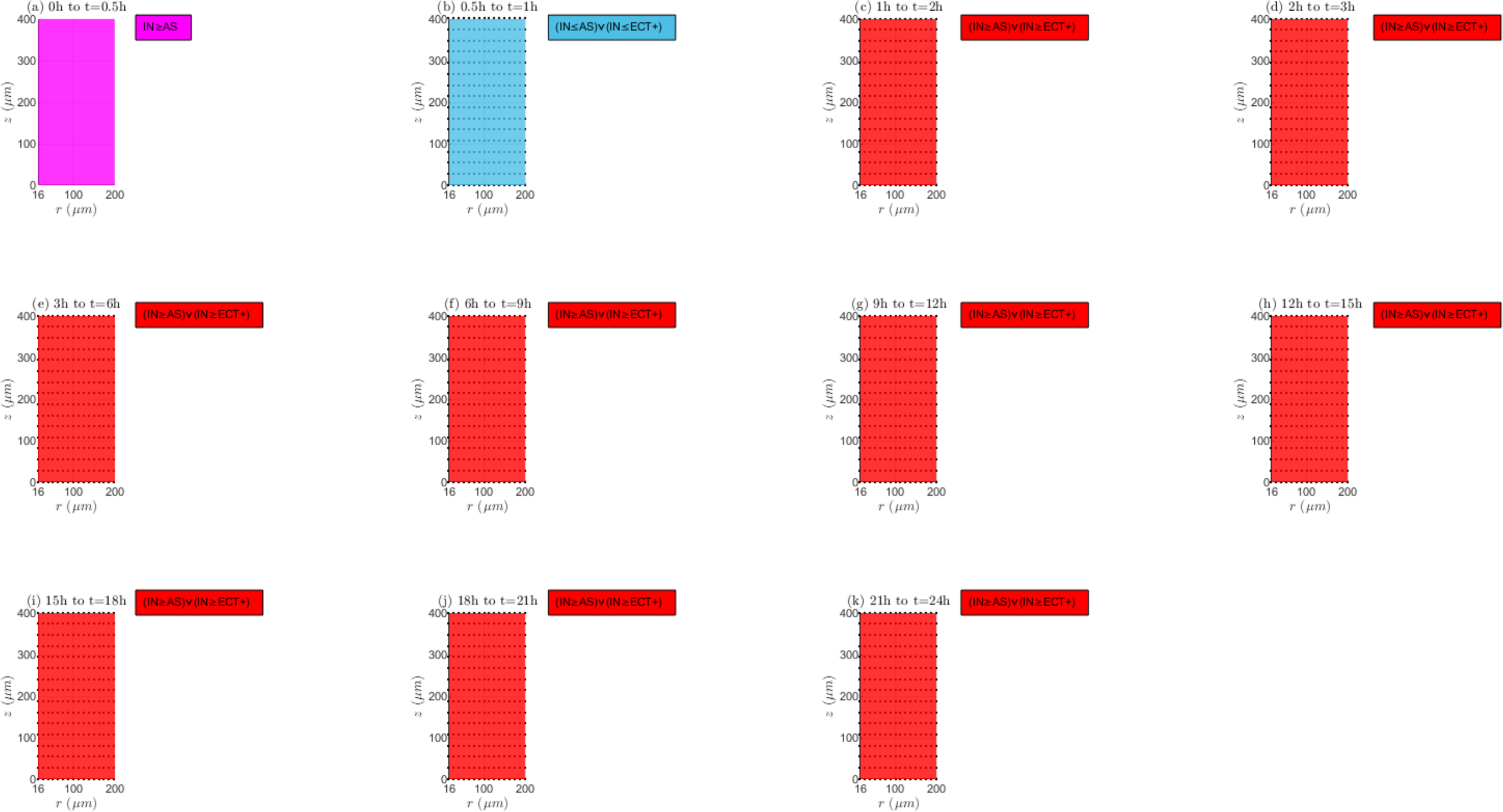

Supplement: S1 Fig — (TIF) [file pone.0315194.s001.tif]

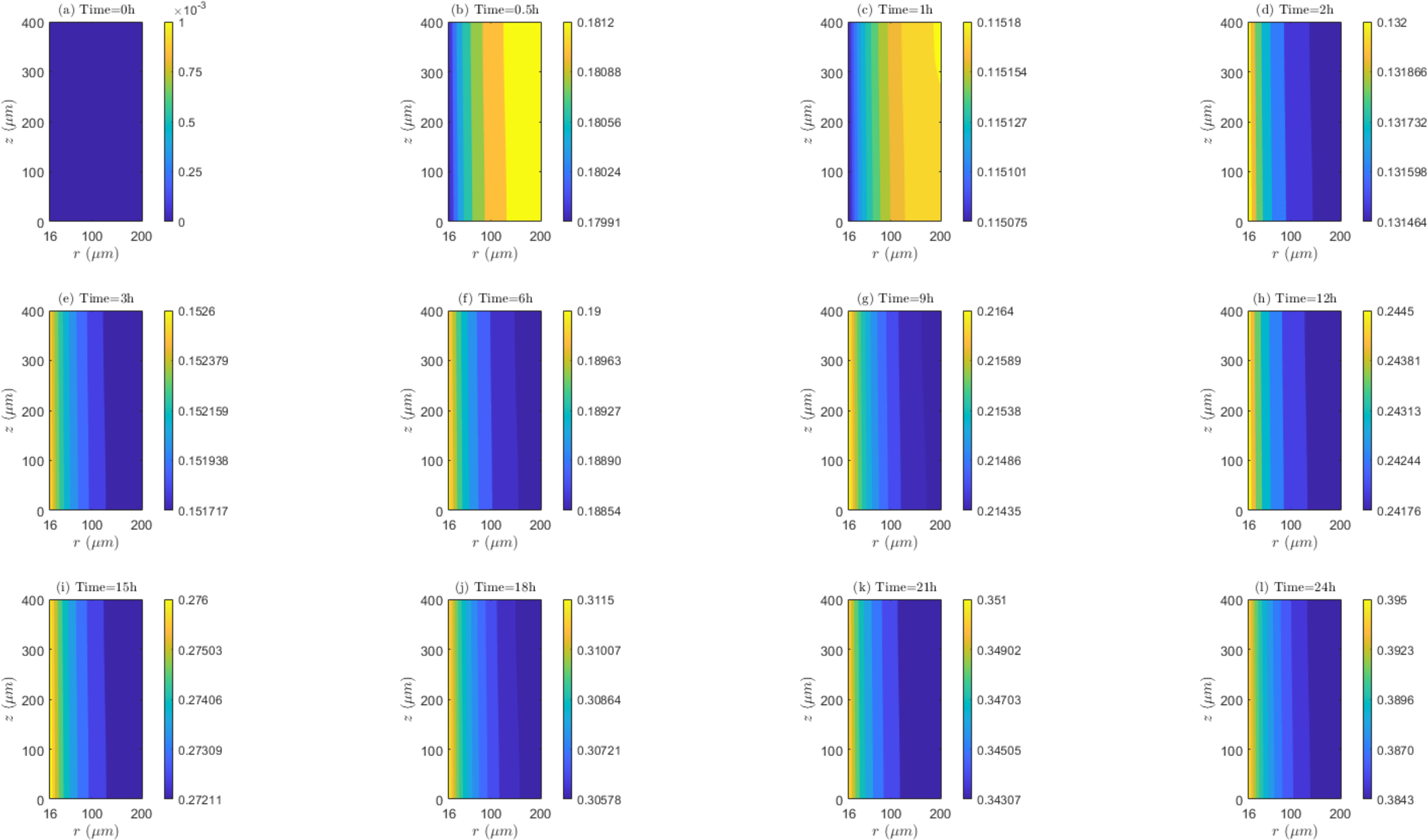

Supplement: S2 Fig — (TIF) [file pone.0315194.s002.tif]

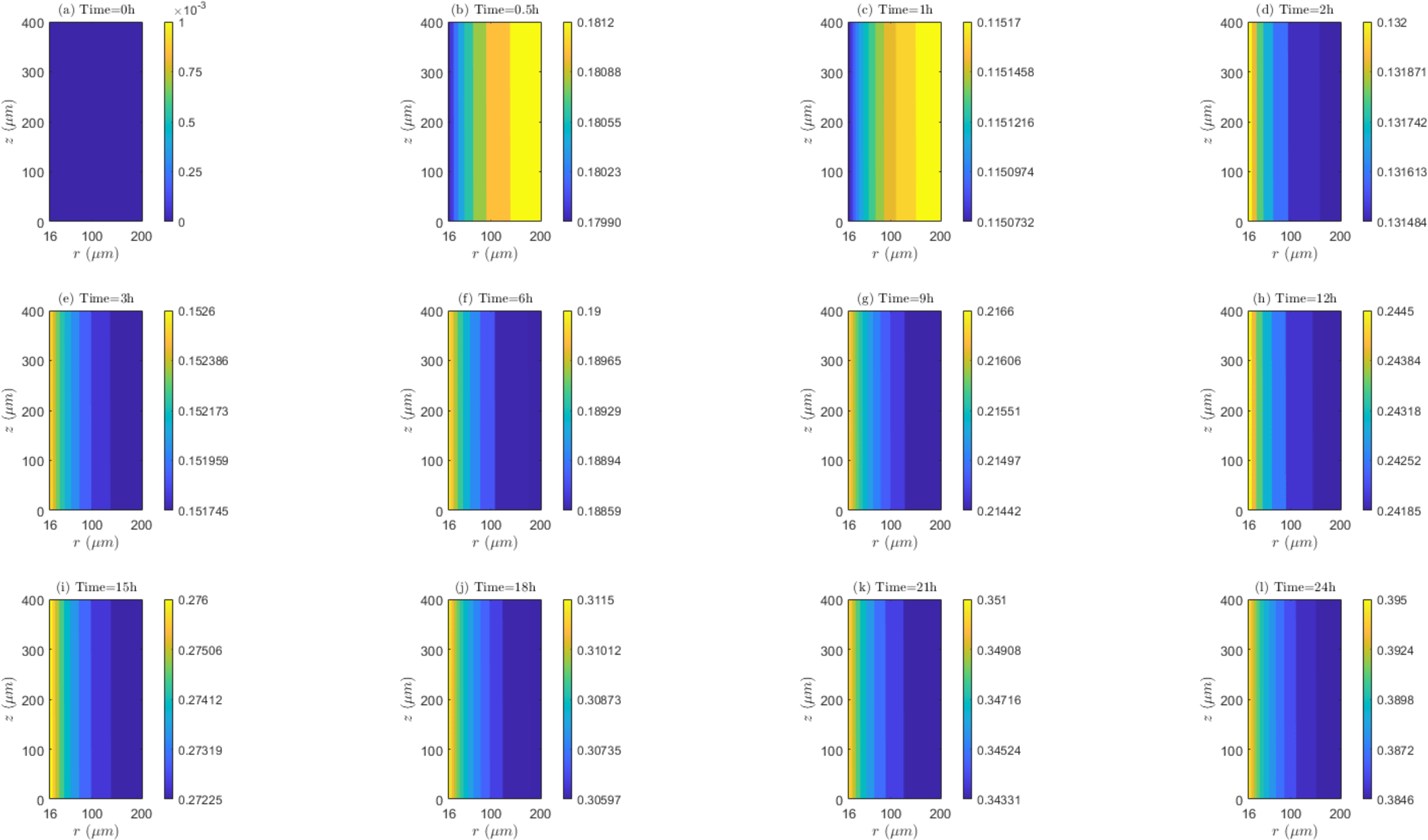

Supplement: S3 Fig — (TIF) [file pone.0315194.s003.tif]

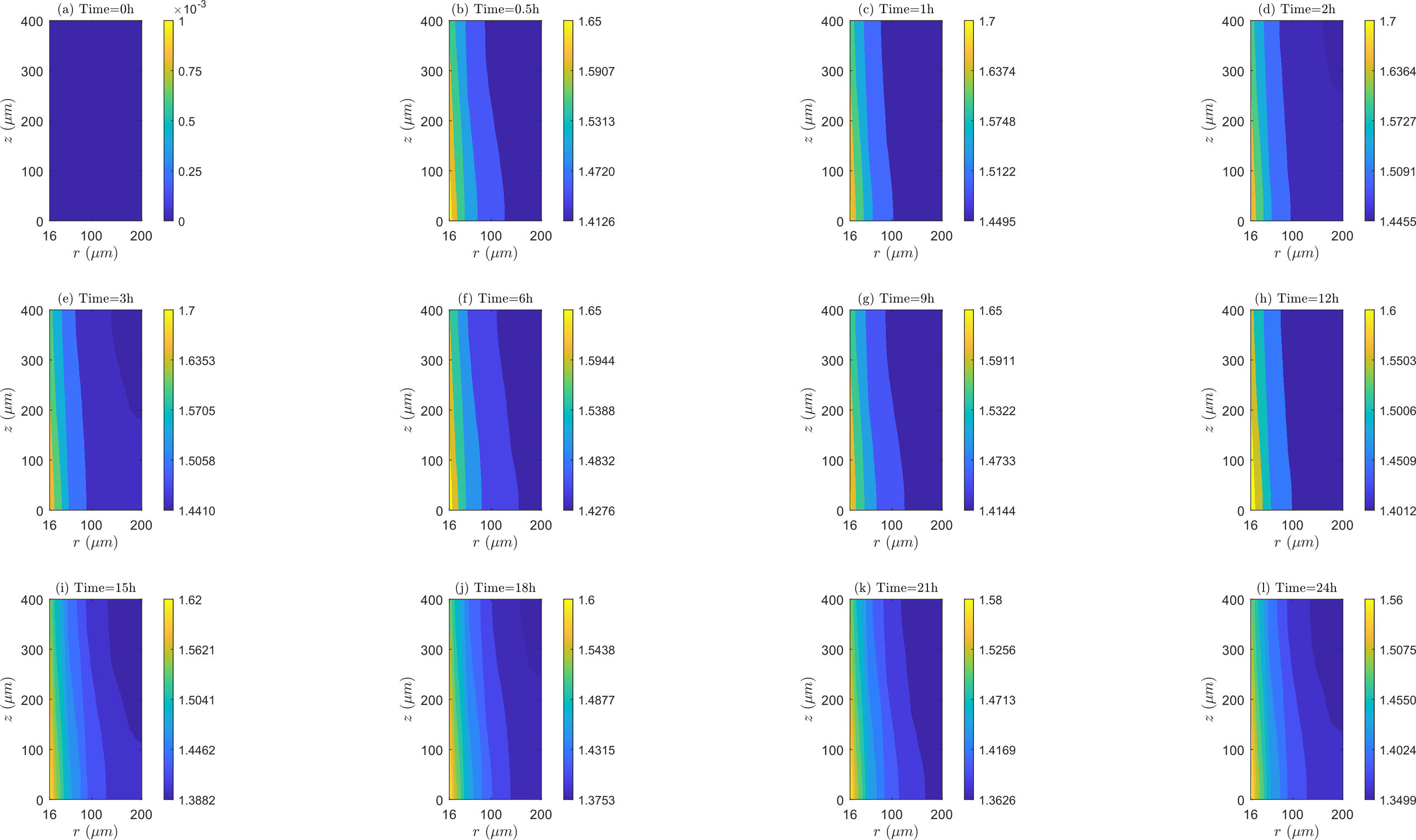

Supplement: S4 Fig — (TIF) [file pone.0315194.s004.tif]

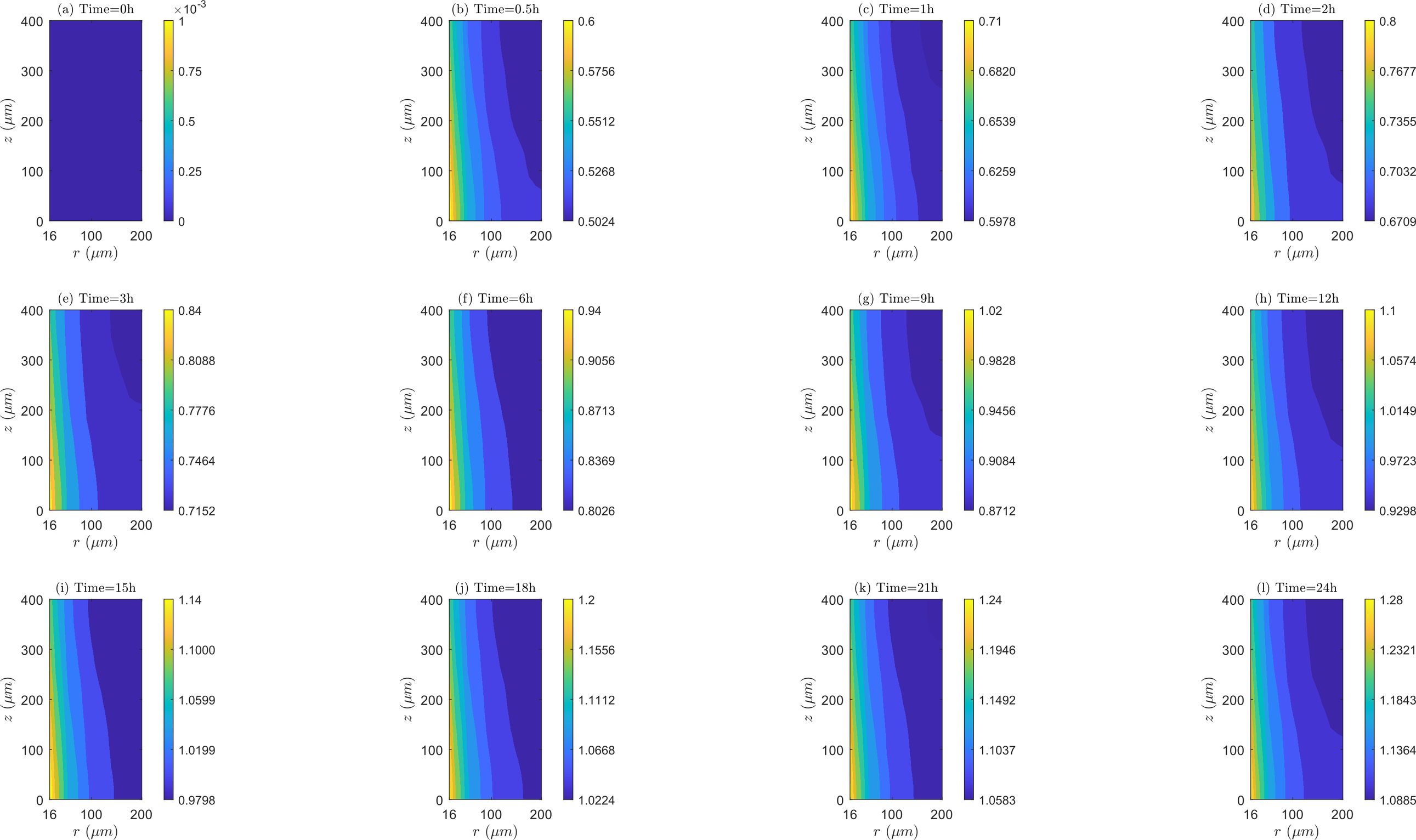

Supplement: S5 Fig — (TIF) [file pone.0315194.s005.tif]

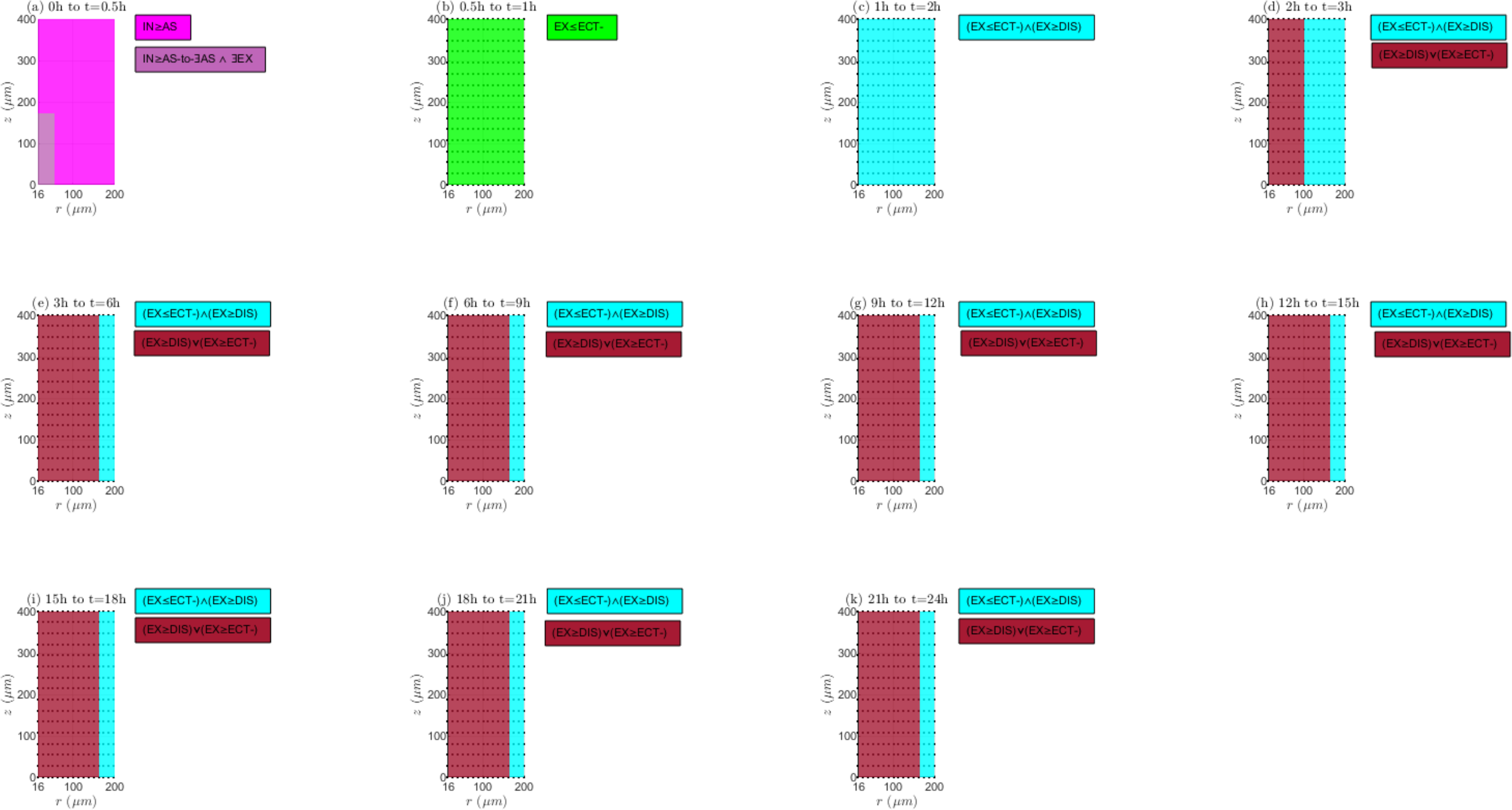

Supplement: S6 Fig — (TIF) [file pone.0315194.s006.tif]

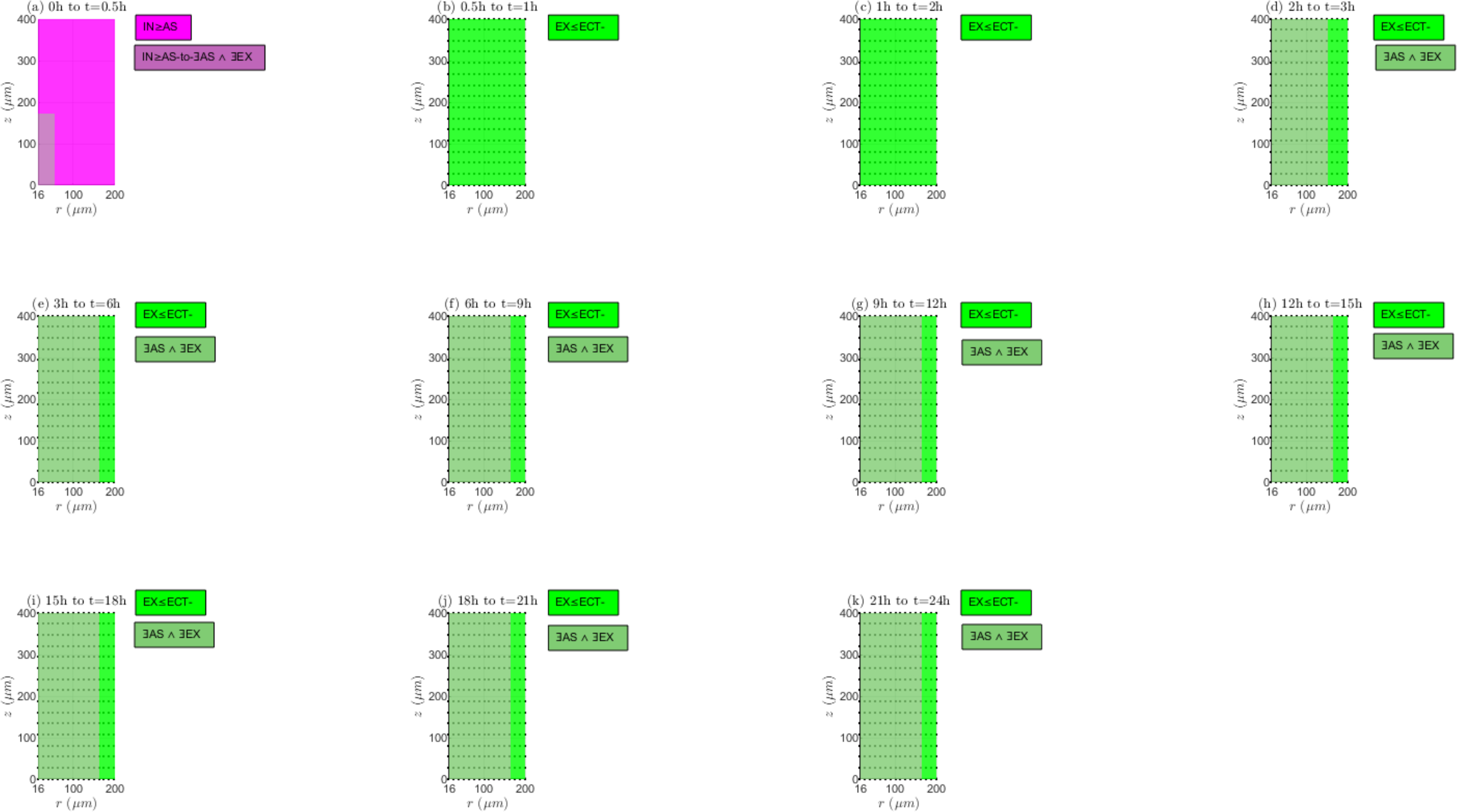

Supplement: S7 Fig — (TIF) [file pone.0315194.s007.tif]

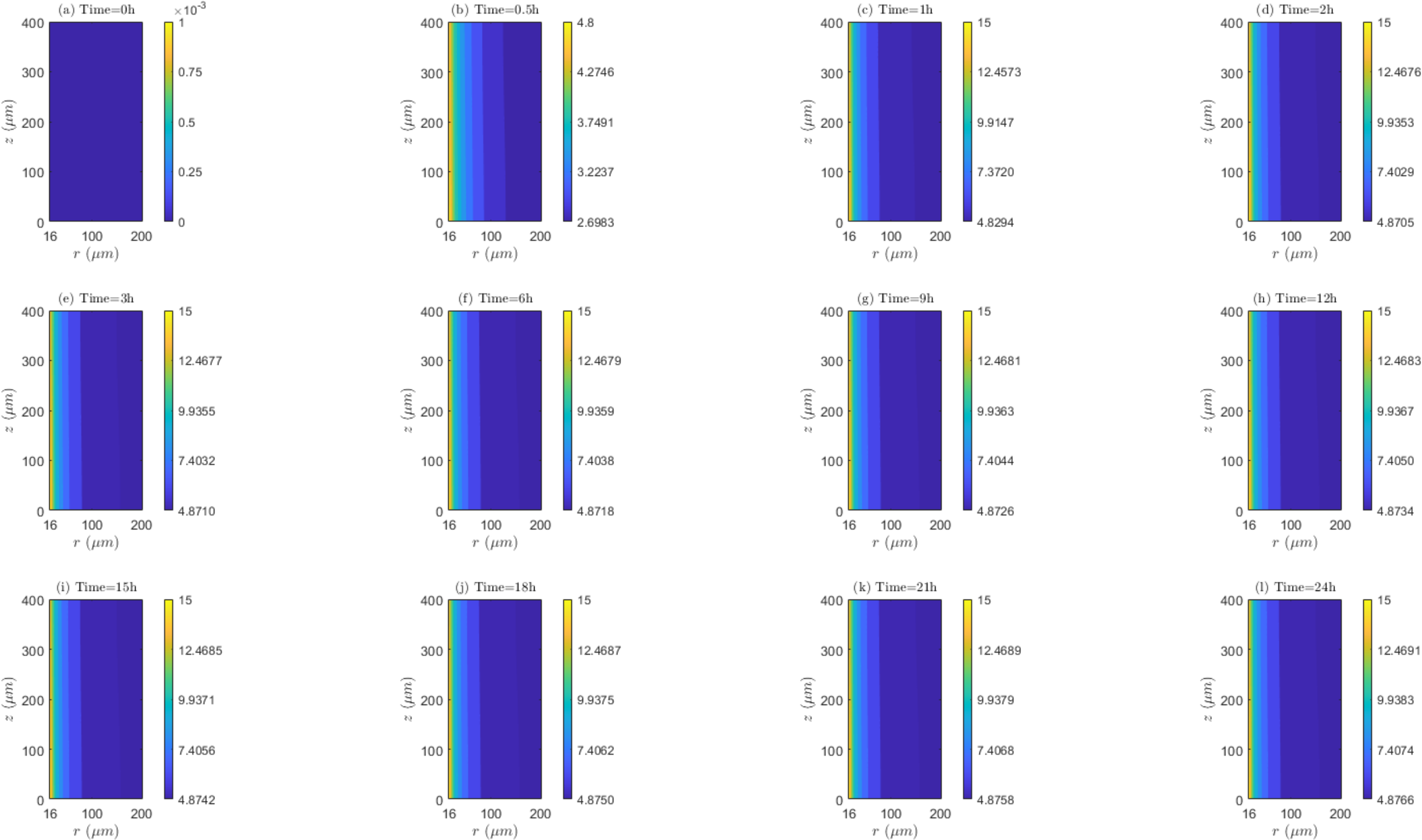

Supplement: S8 Fig — (TIF) [file pone.0315194.s008.tif]

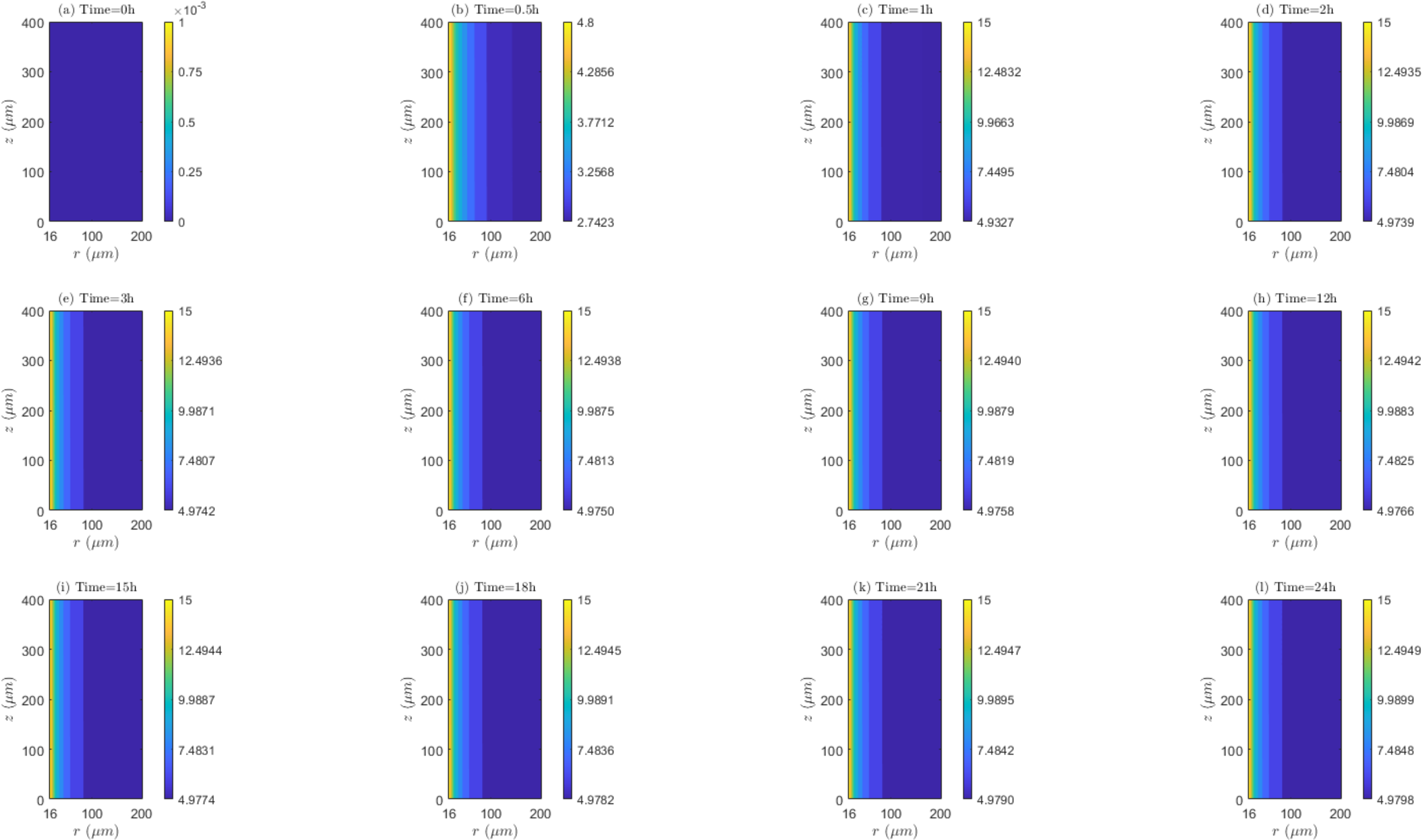

Supplement: S9 Fig — (TIF) [file pone.0315194.s009.tif]

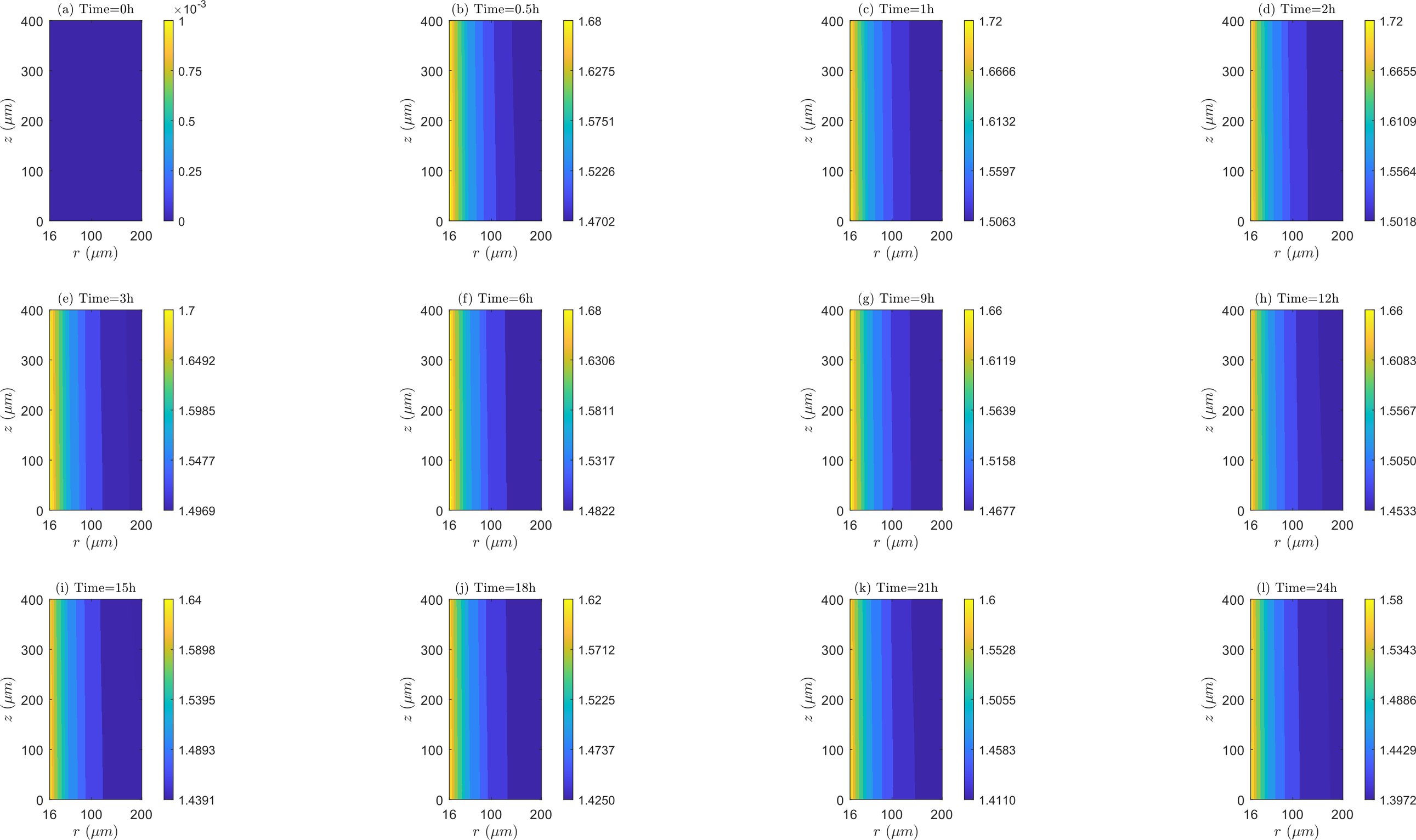

Supplement: S10 Fig — (TIF) [file pone.0315194.s010.tif]

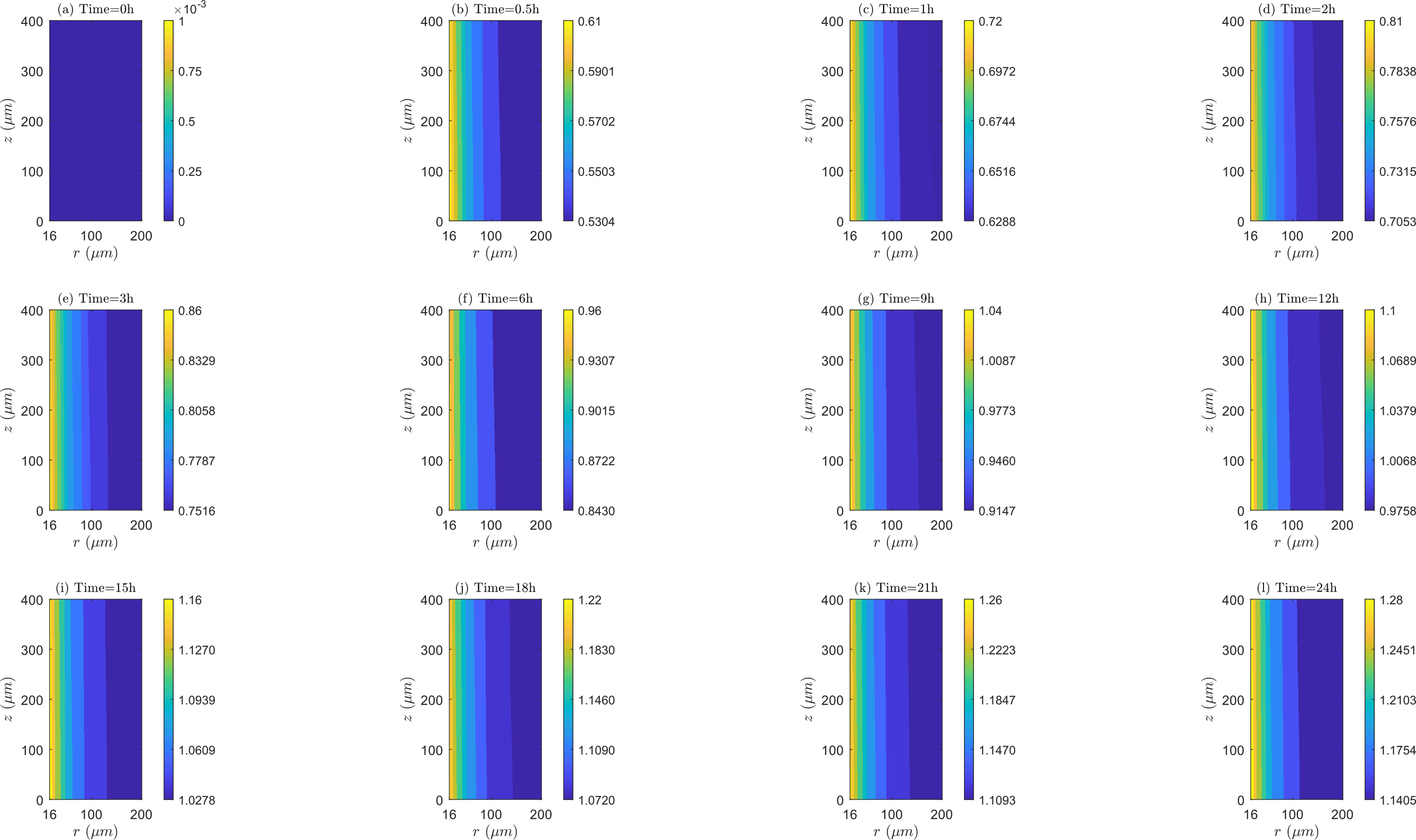

Supplement: S11 Fig — (TIF) [file pone.0315194.s011.tif]

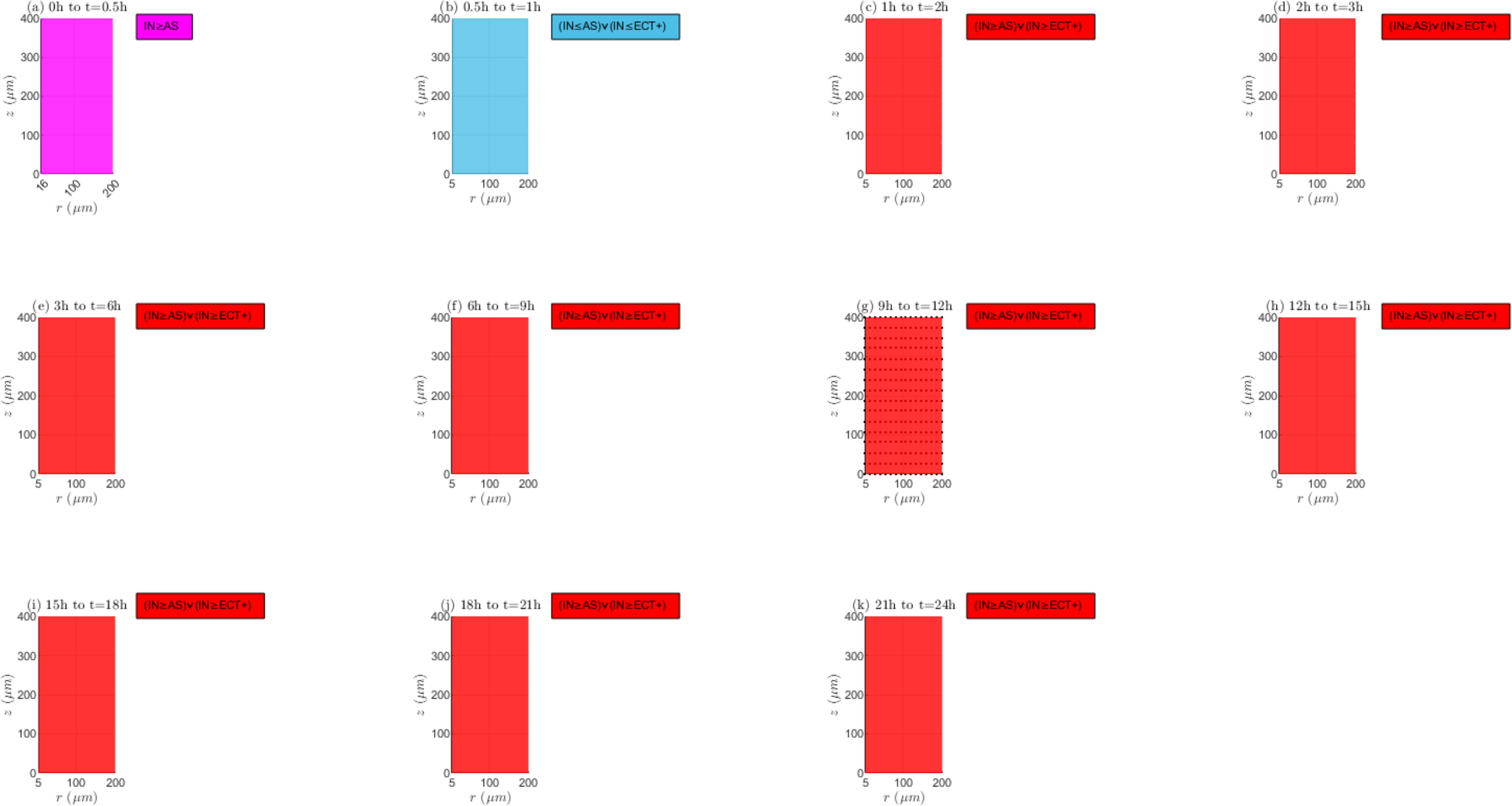

Supplement: S12 Fig — (TIF) [file pone.0315194.s012.tif]

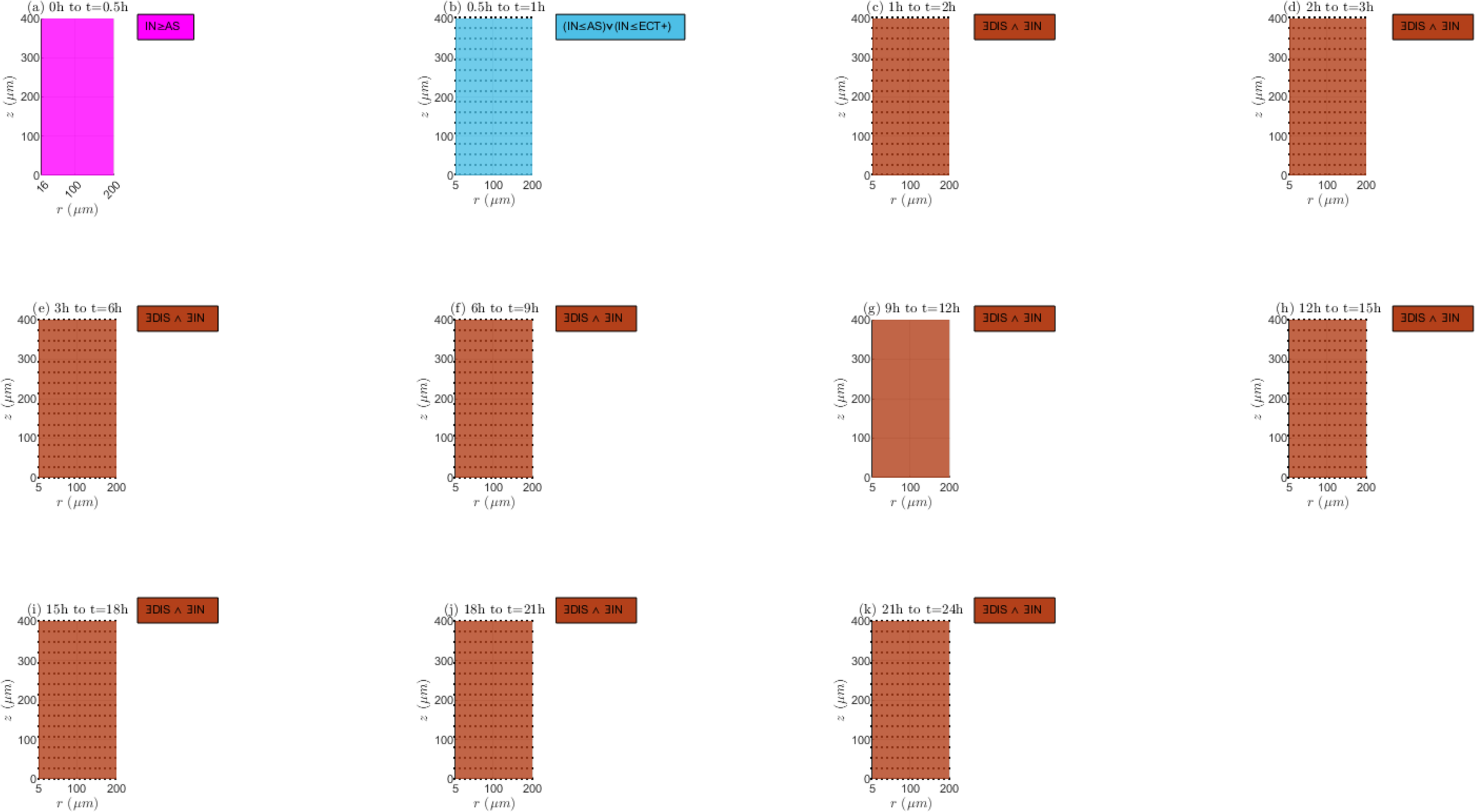

Supplement: S13 Fig — (TIF) [file pone.0315194.s013.tif]

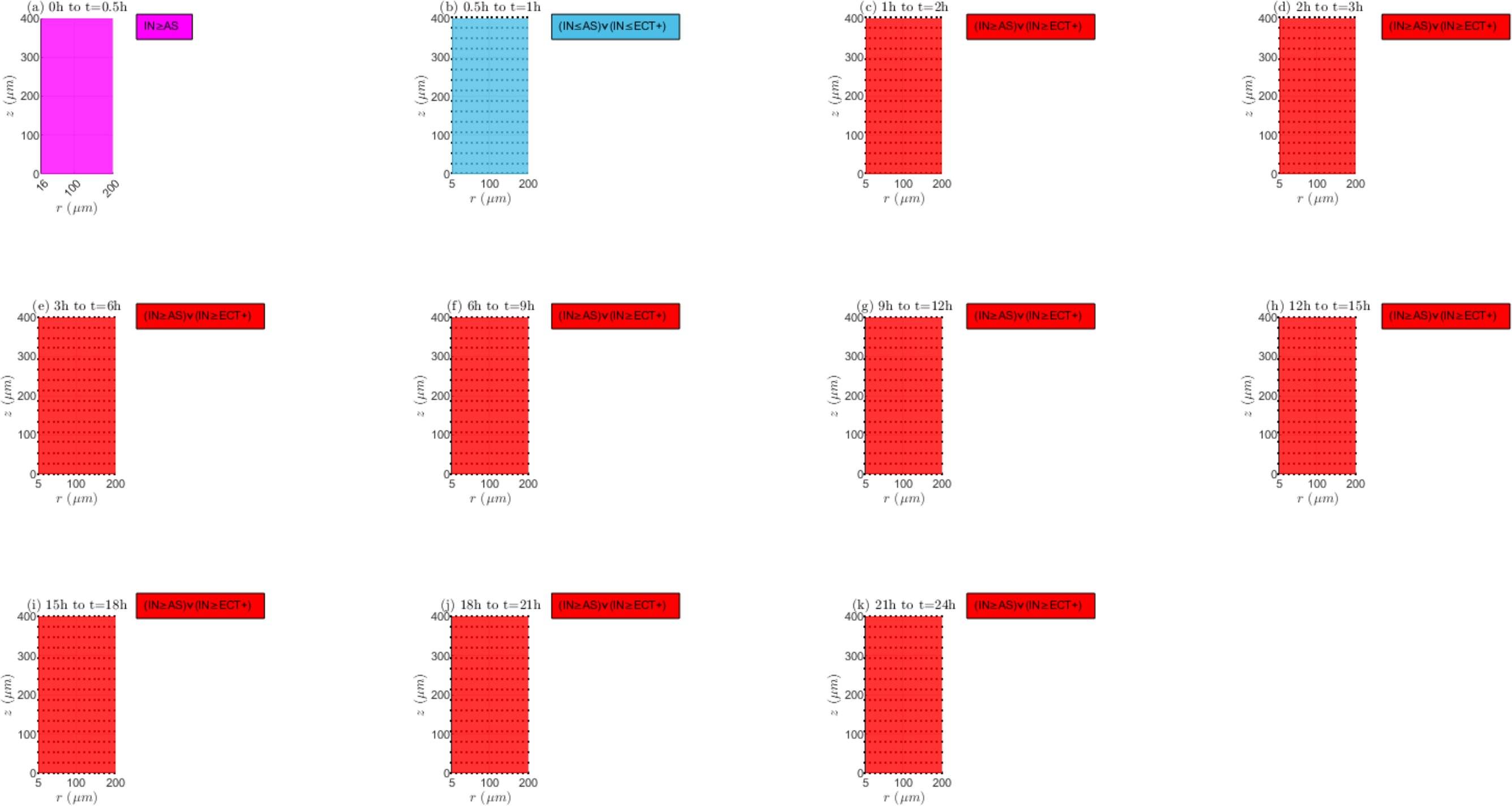

Supplement: S14 Fig — (TIF) [file pone.0315194.s014.tif]

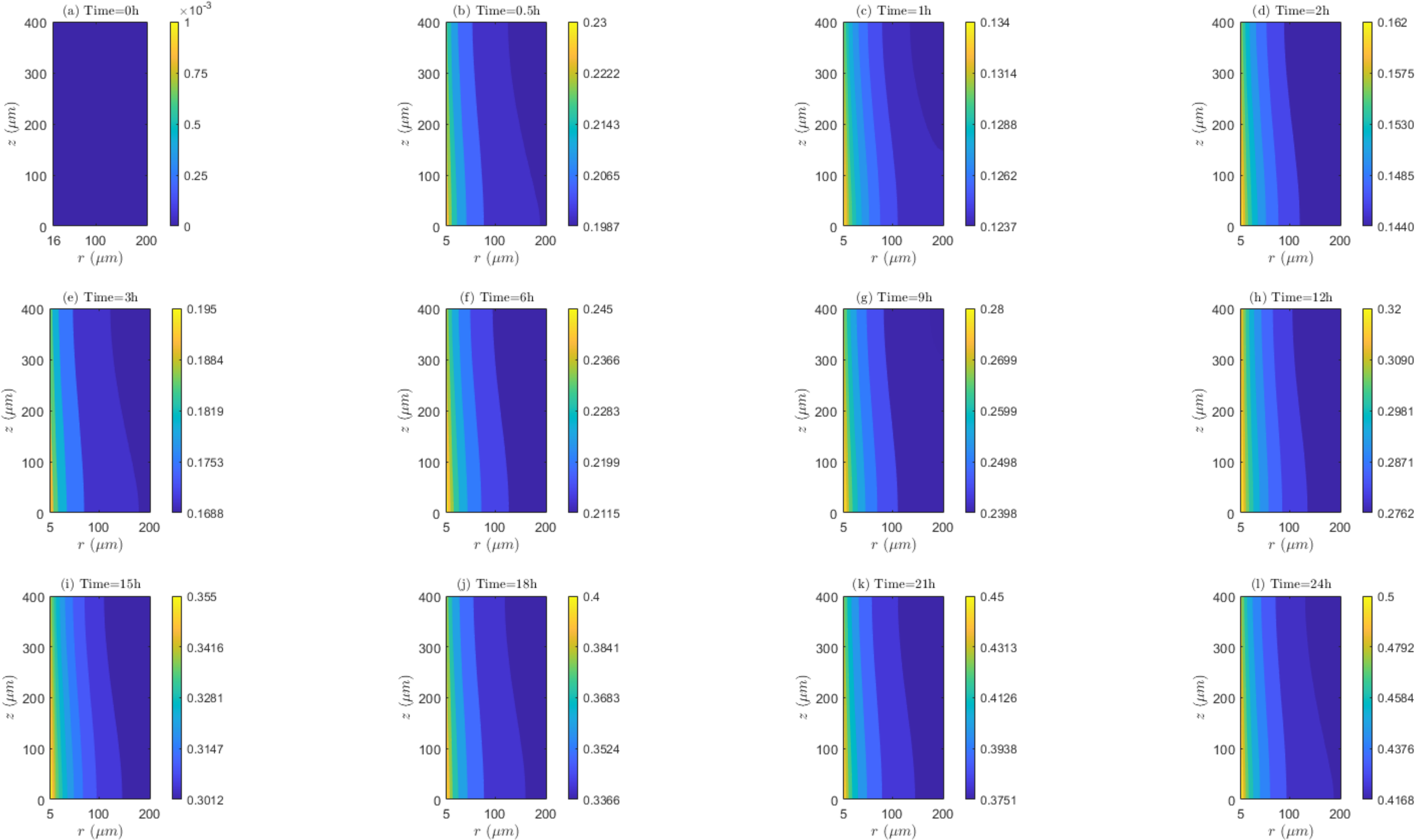

Supplement: S15 Fig — (TIF) [file pone.0315194.s015.tif]

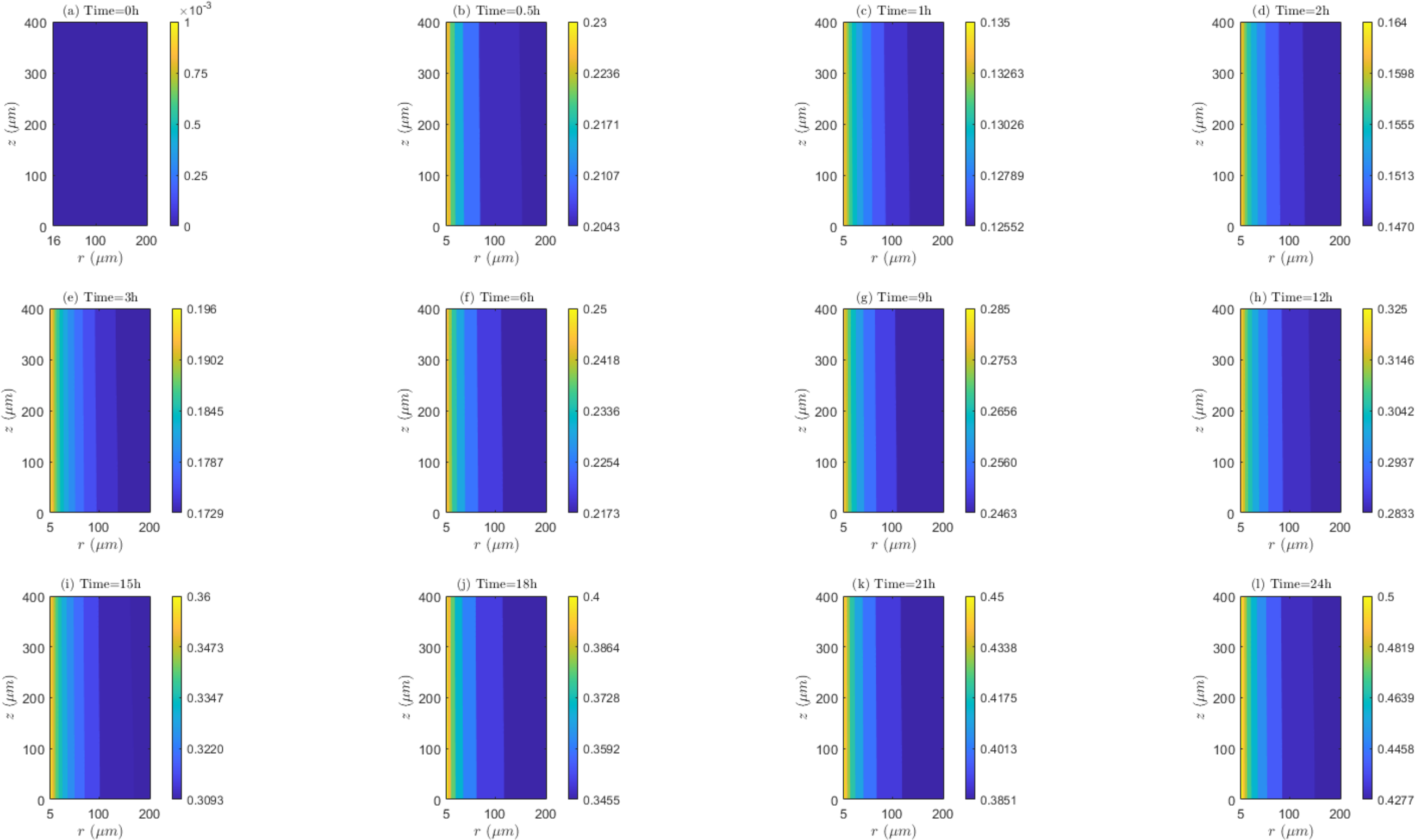

Supplement: S16 Fig — (TIF) [file pone.0315194.s016.tif]

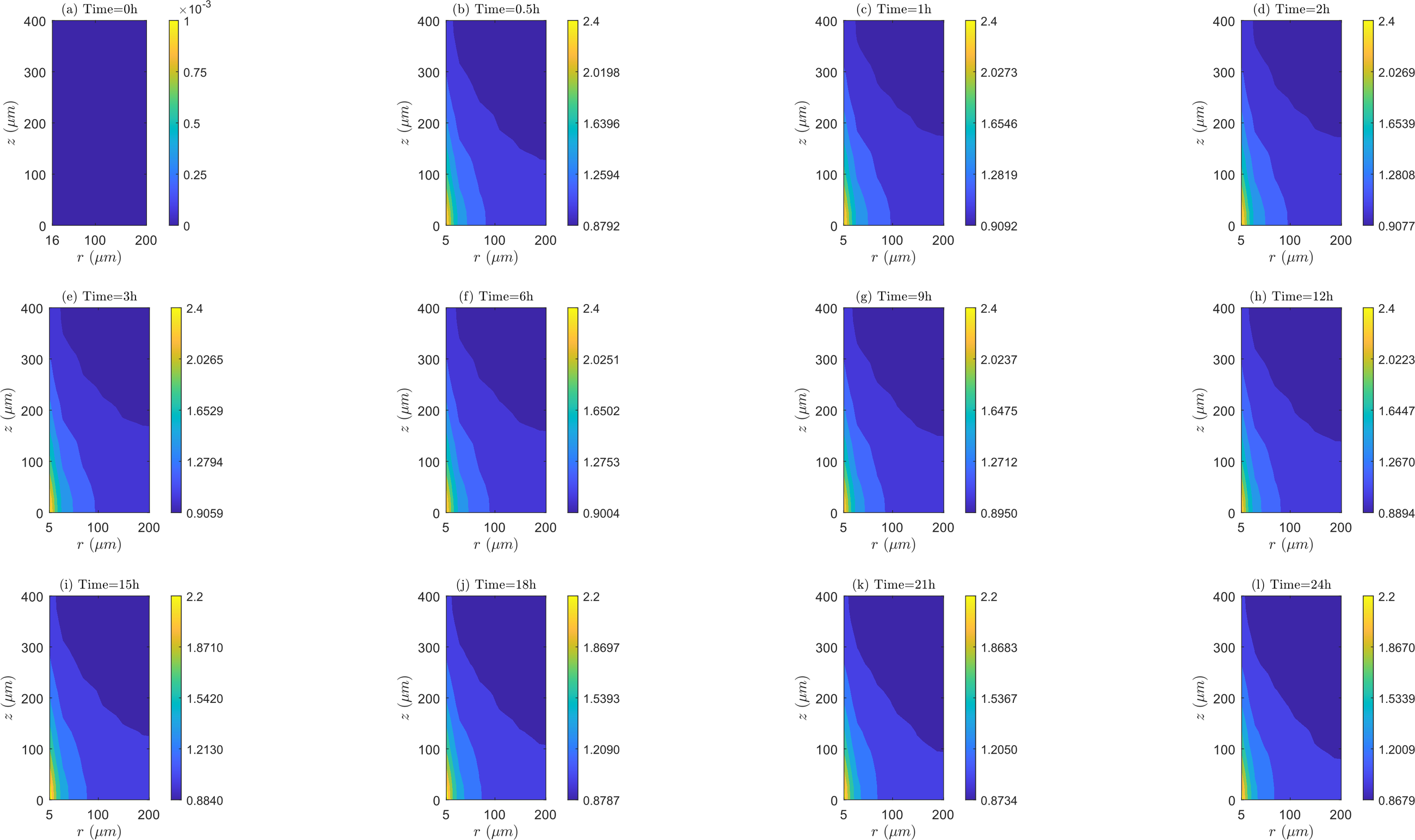

Supplement: S17 Fig — (TIF) [file pone.0315194.s017.tif]

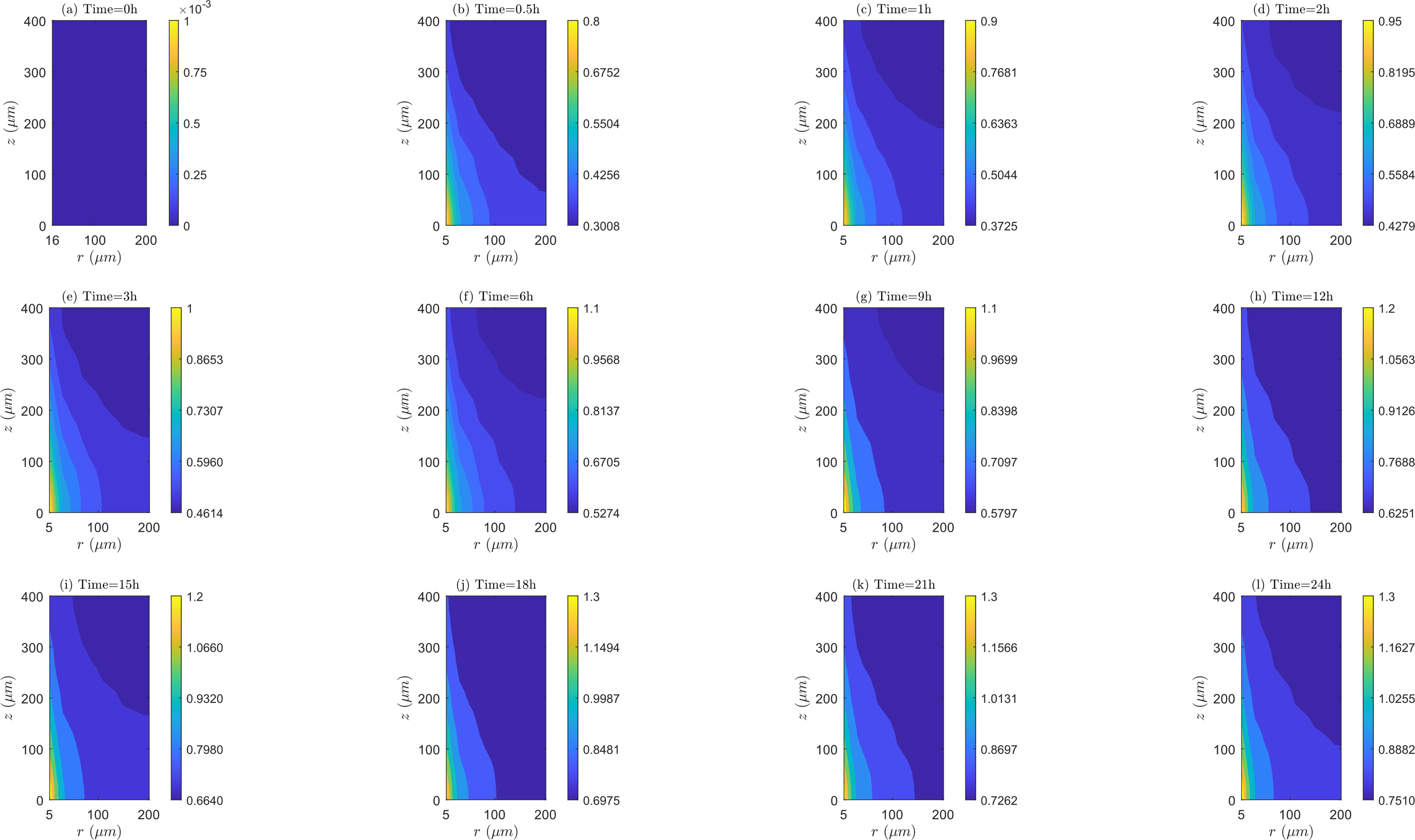

Supplement: S18 Fig — (TIF) [file pone.0315194.s018.tif]

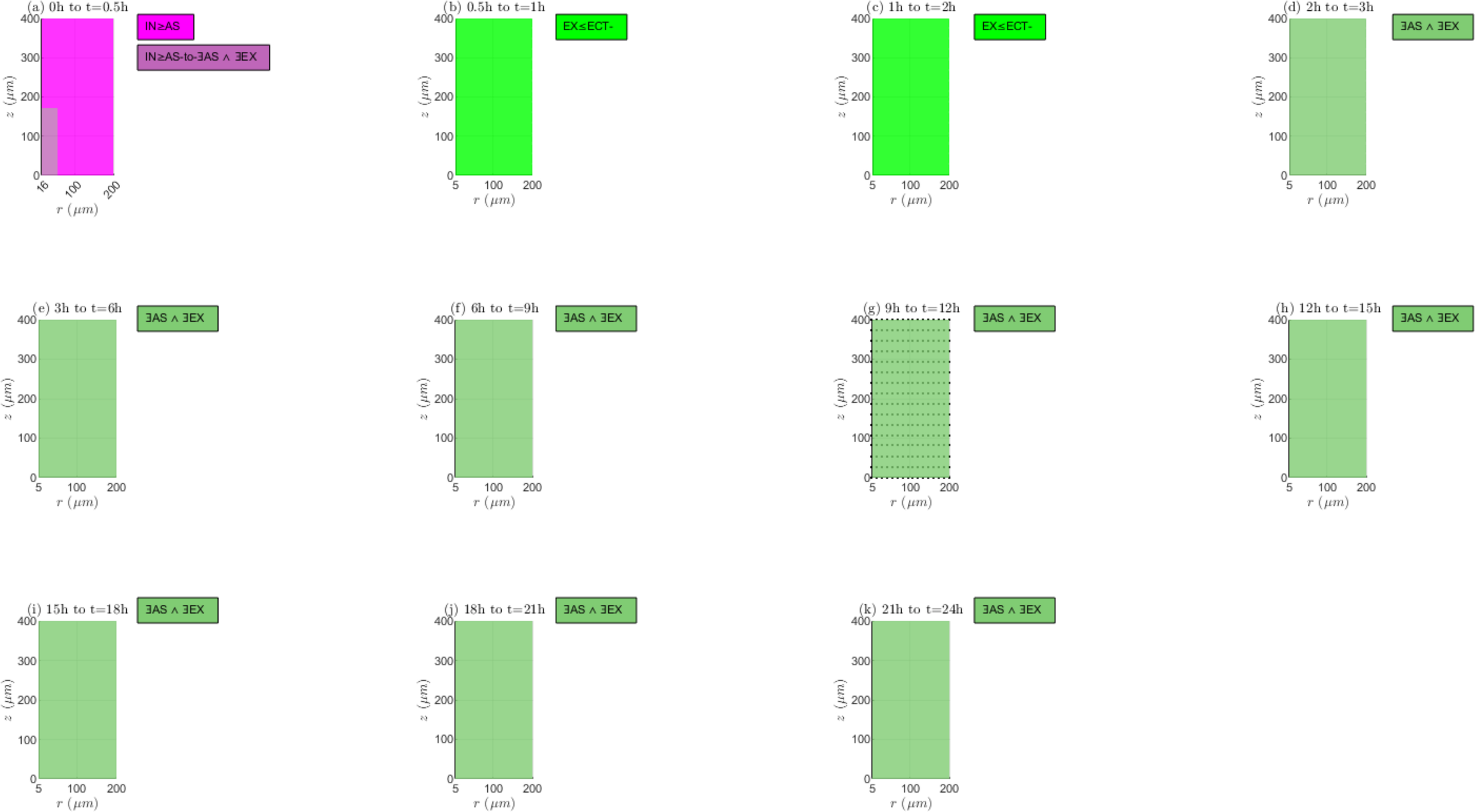

Supplement: S19 Fig — (TIF) [file pone.0315194.s019.tif]

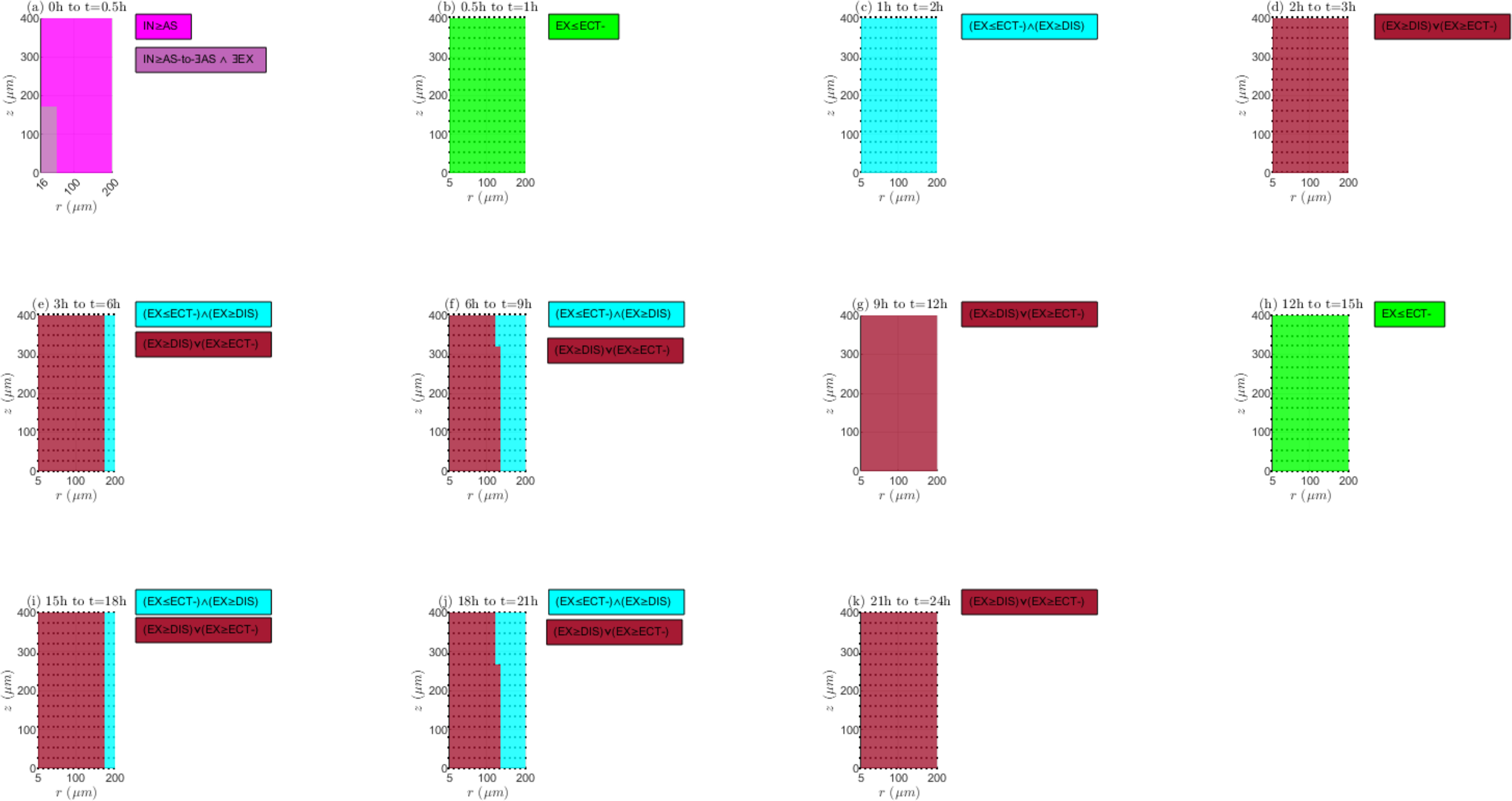

Supplement: S20 Fig — (TIF) [file pone.0315194.s020.tif]

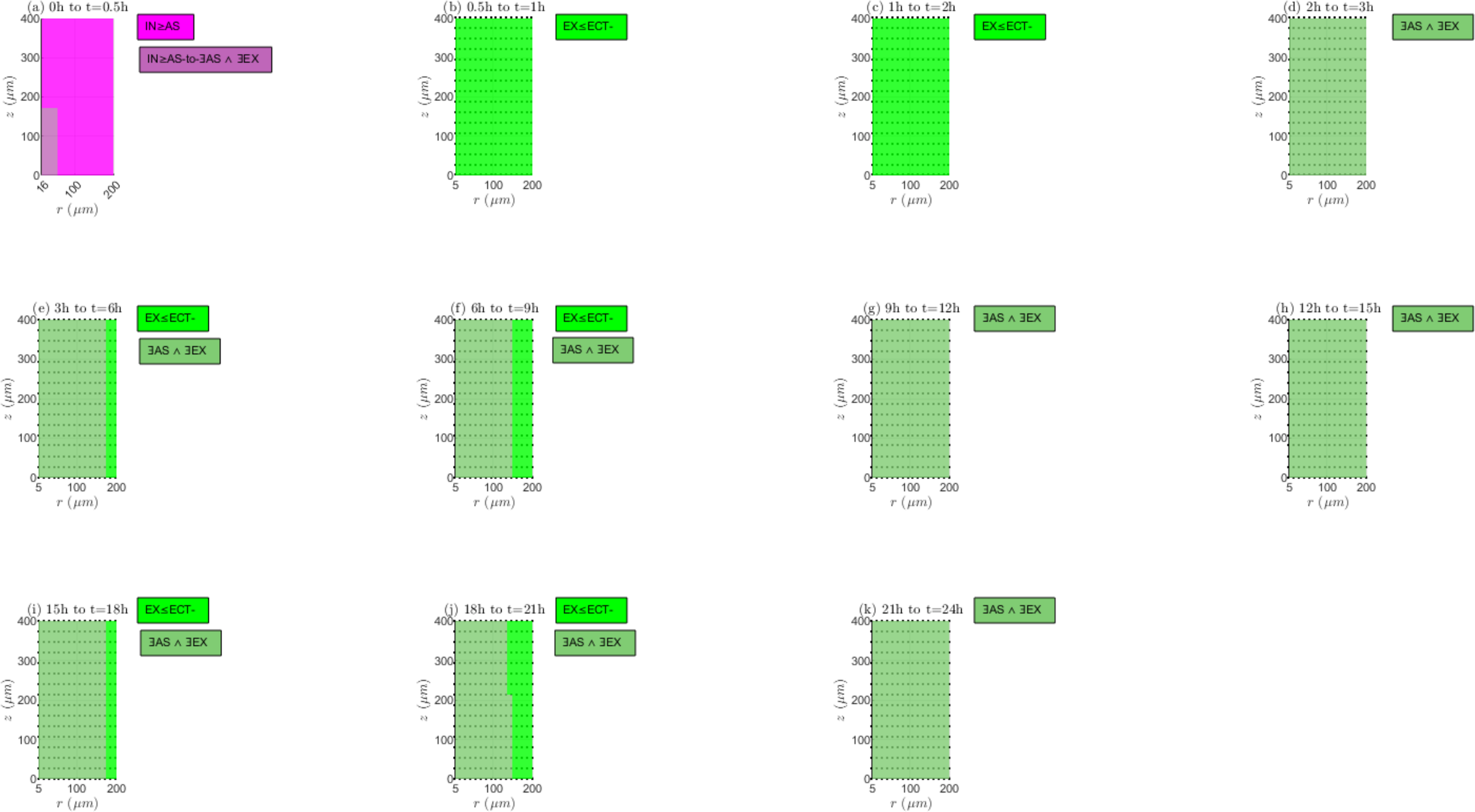

Supplement: S21 Fig — (TIF) [file pone.0315194.s021.tif]

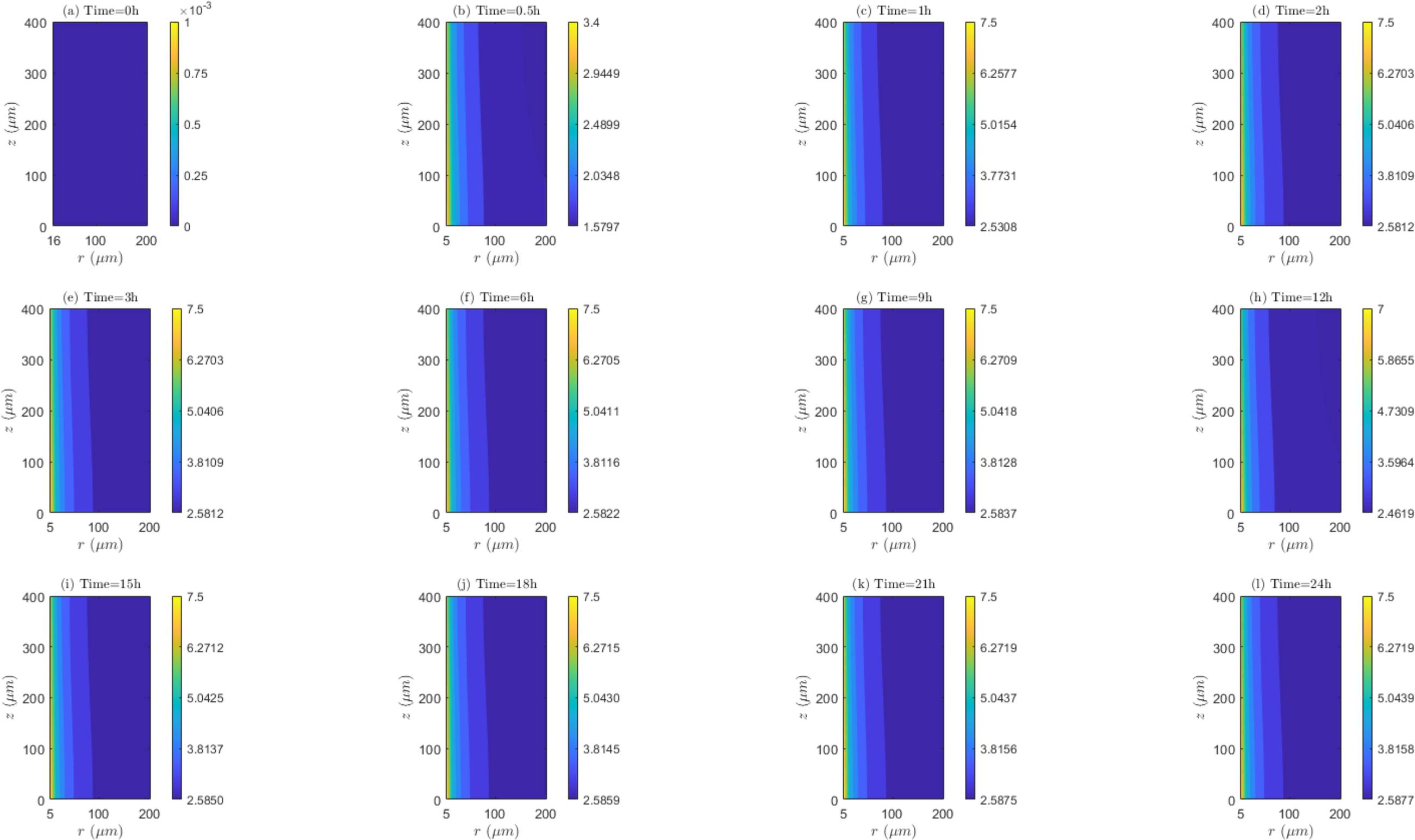

Supplement: S22 Fig — (TIF) [file pone.0315194.s022.tif]

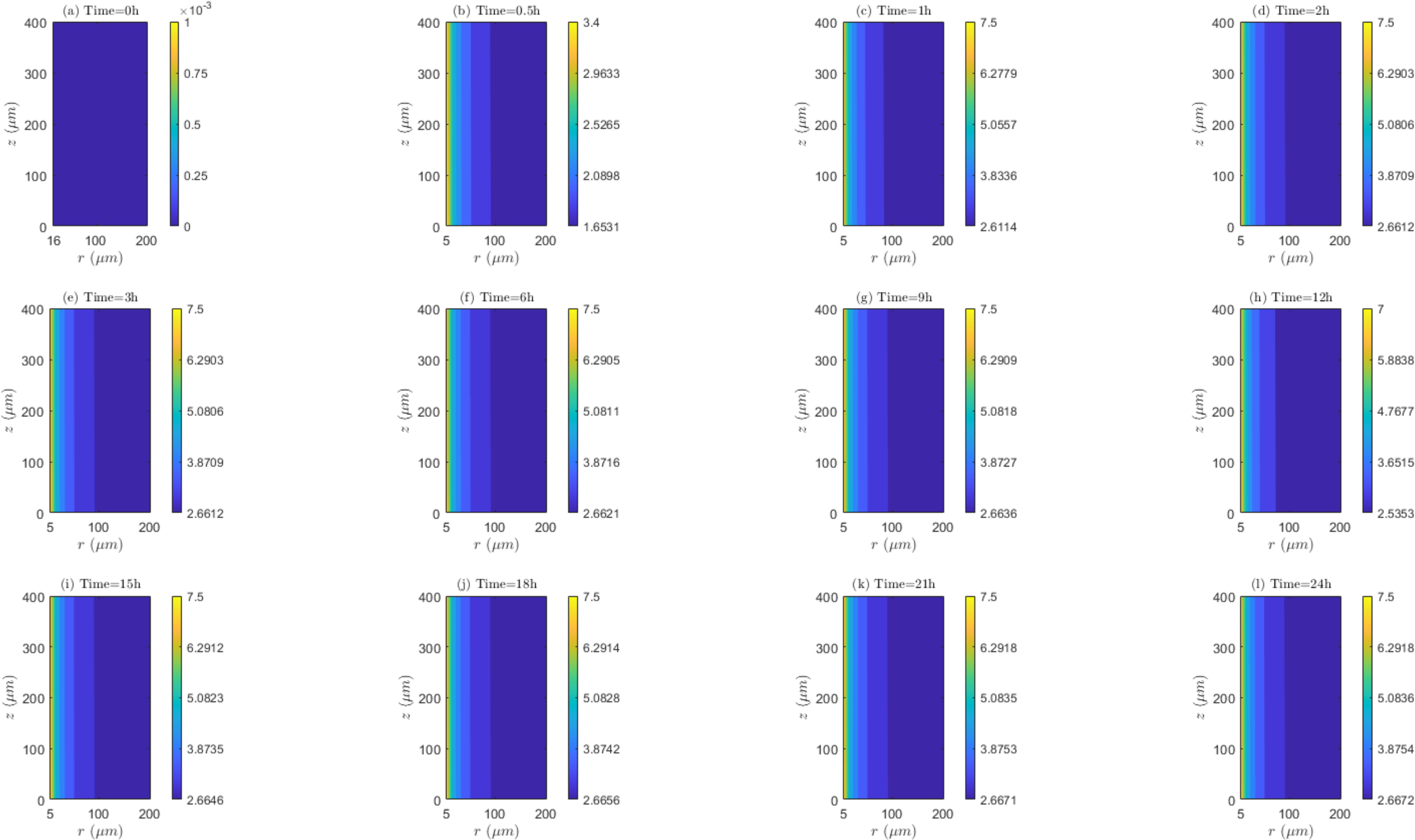

Supplement: S23 Fig — (TIF) [file pone.0315194.s023.tif]

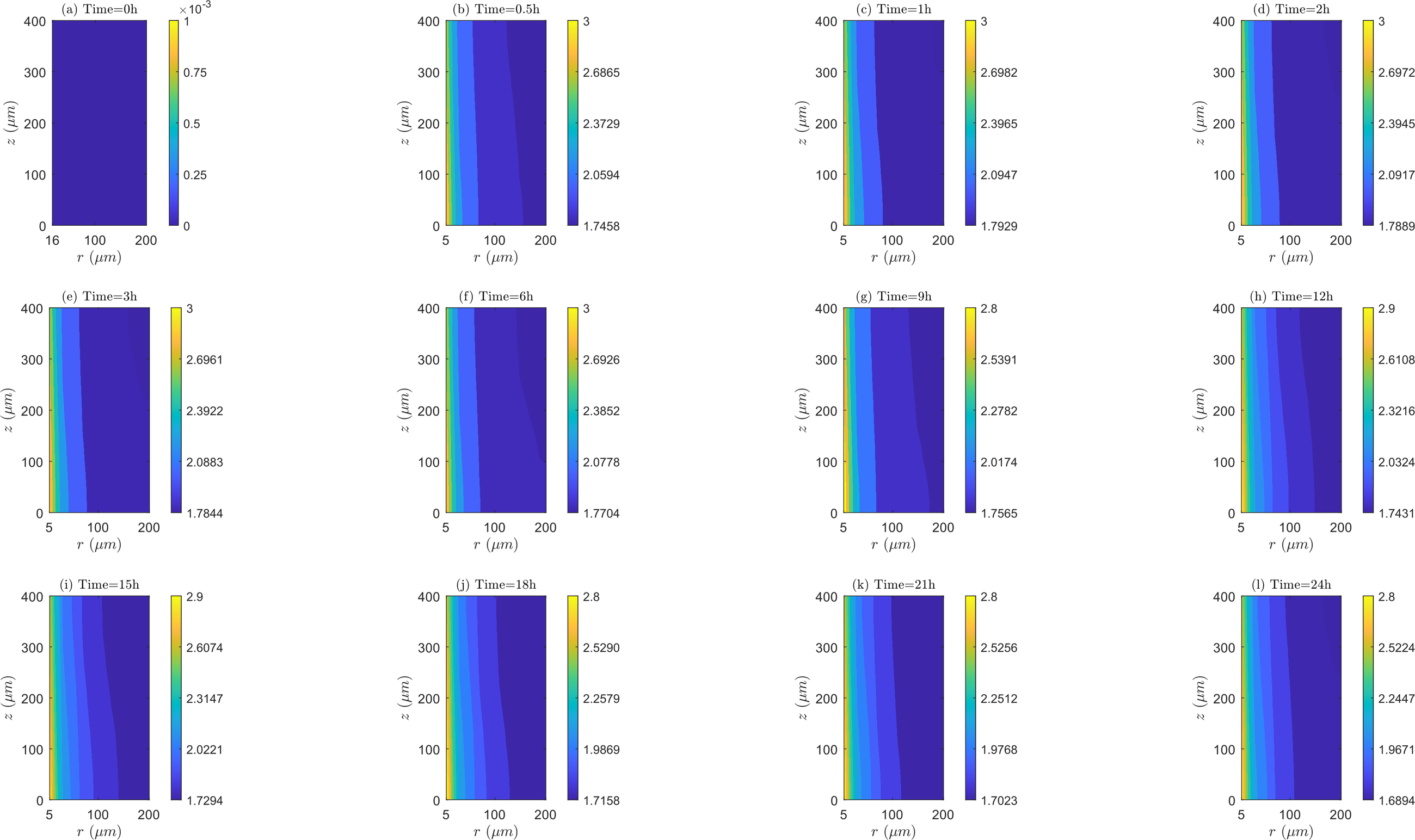

Supplement: S24 Fig — (TIF) [file pone.0315194.s024.tif]

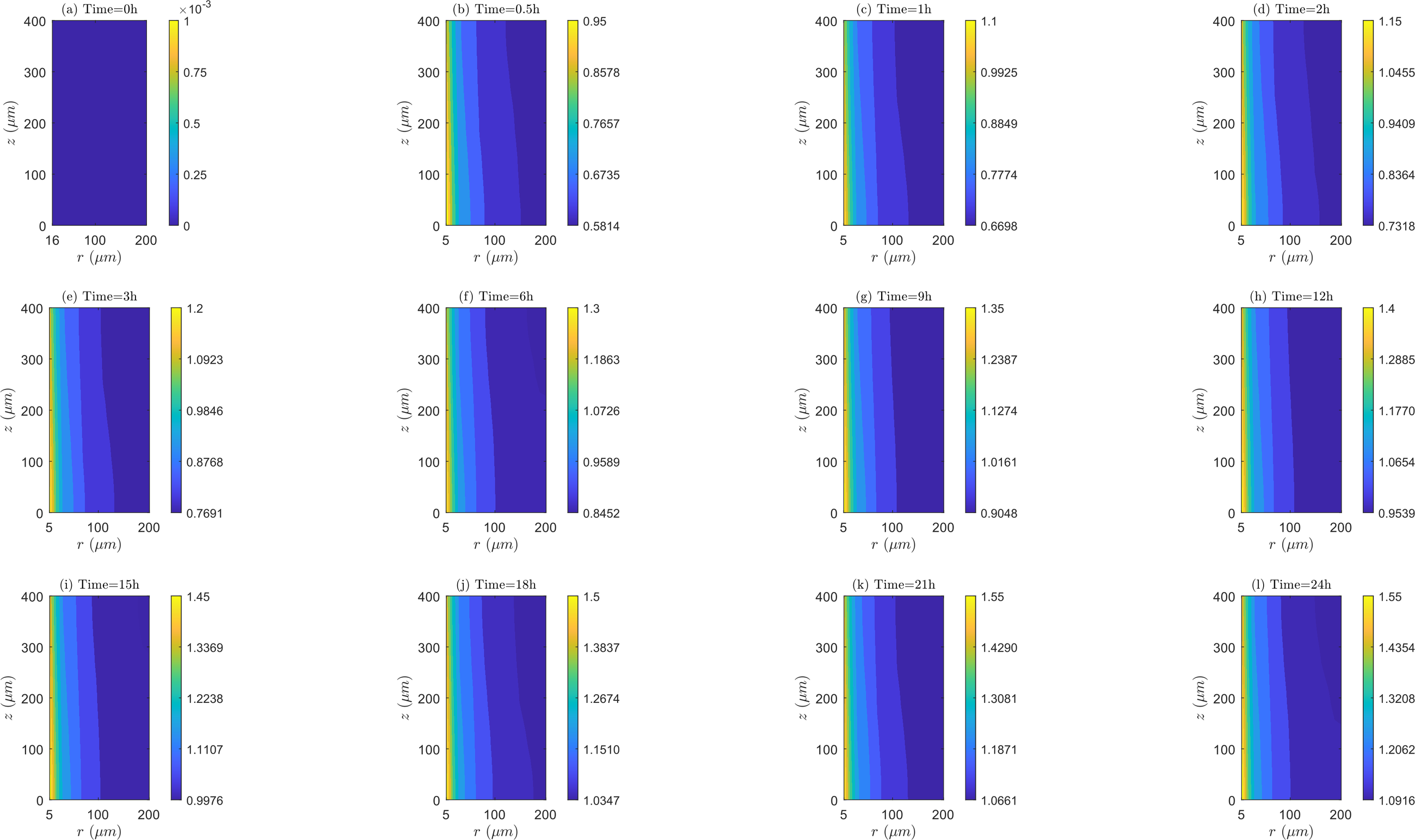

Supplement: S25 Fig — (TIF) [file pone.0315194.s025.tif]

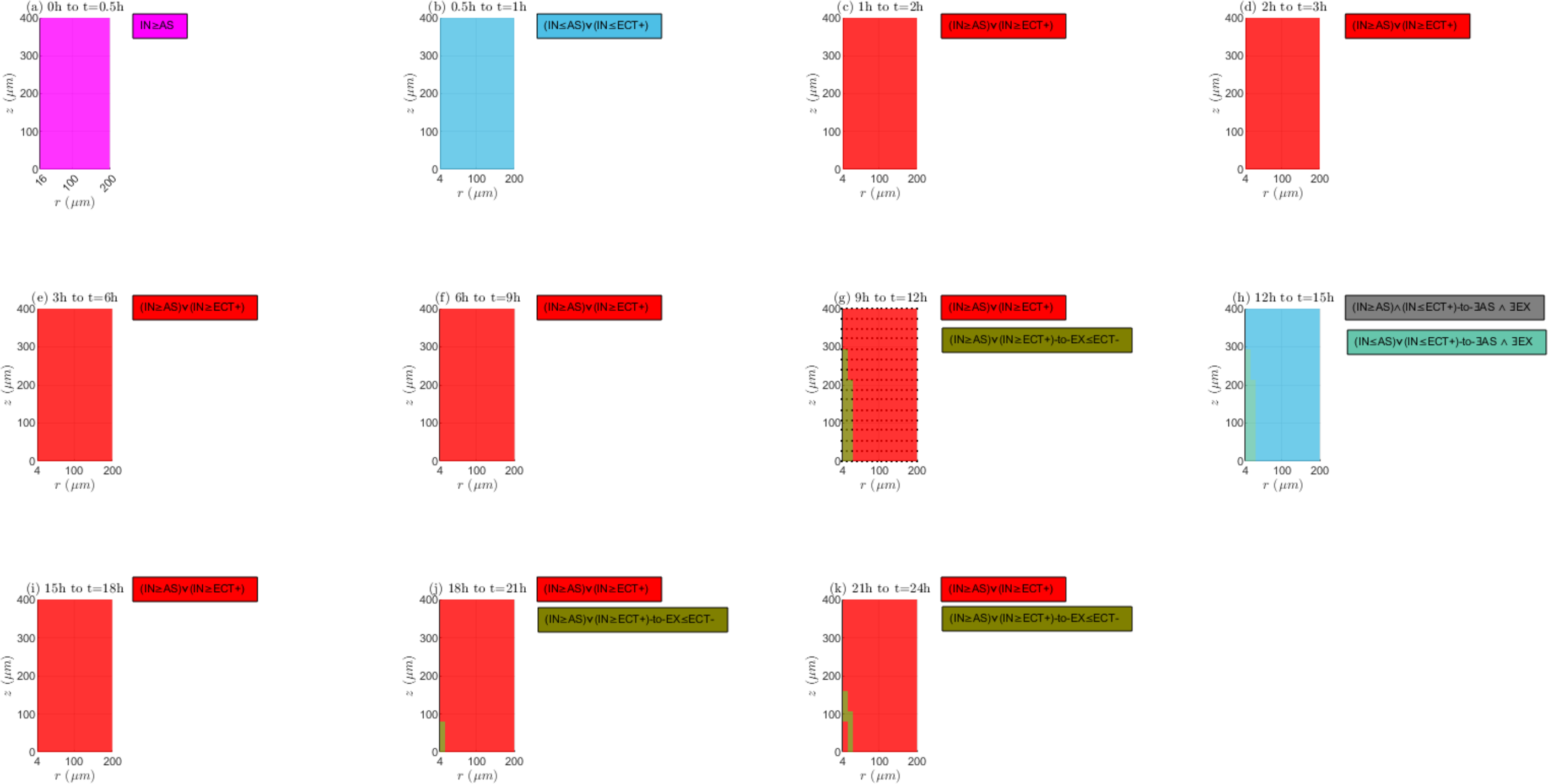

Supplement: S26 Fig — (TIF) [file pone.0315194.s026.tif]

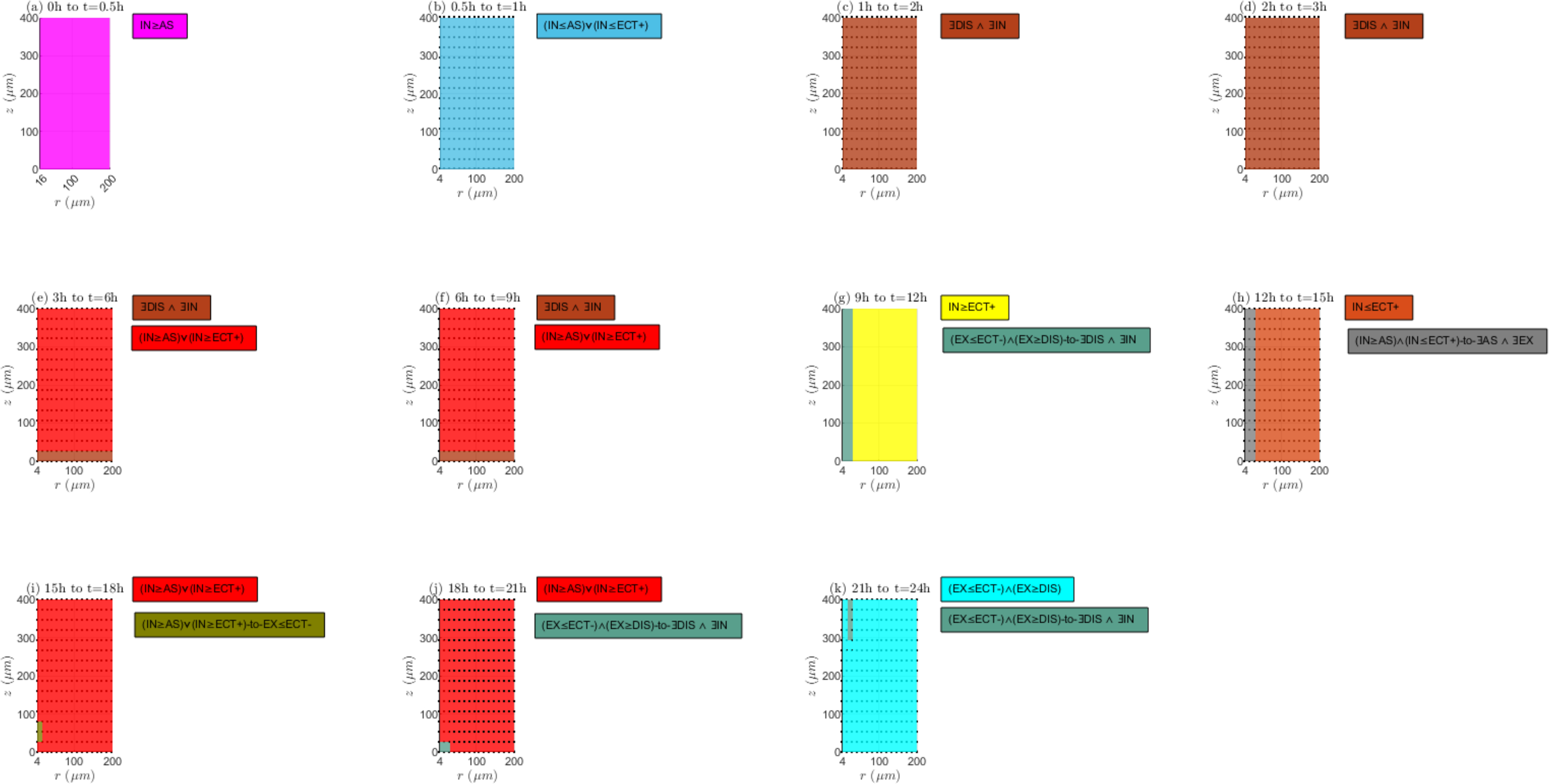

Supplement: S27 Fig — (TIF) [file pone.0315194.s027.tif]

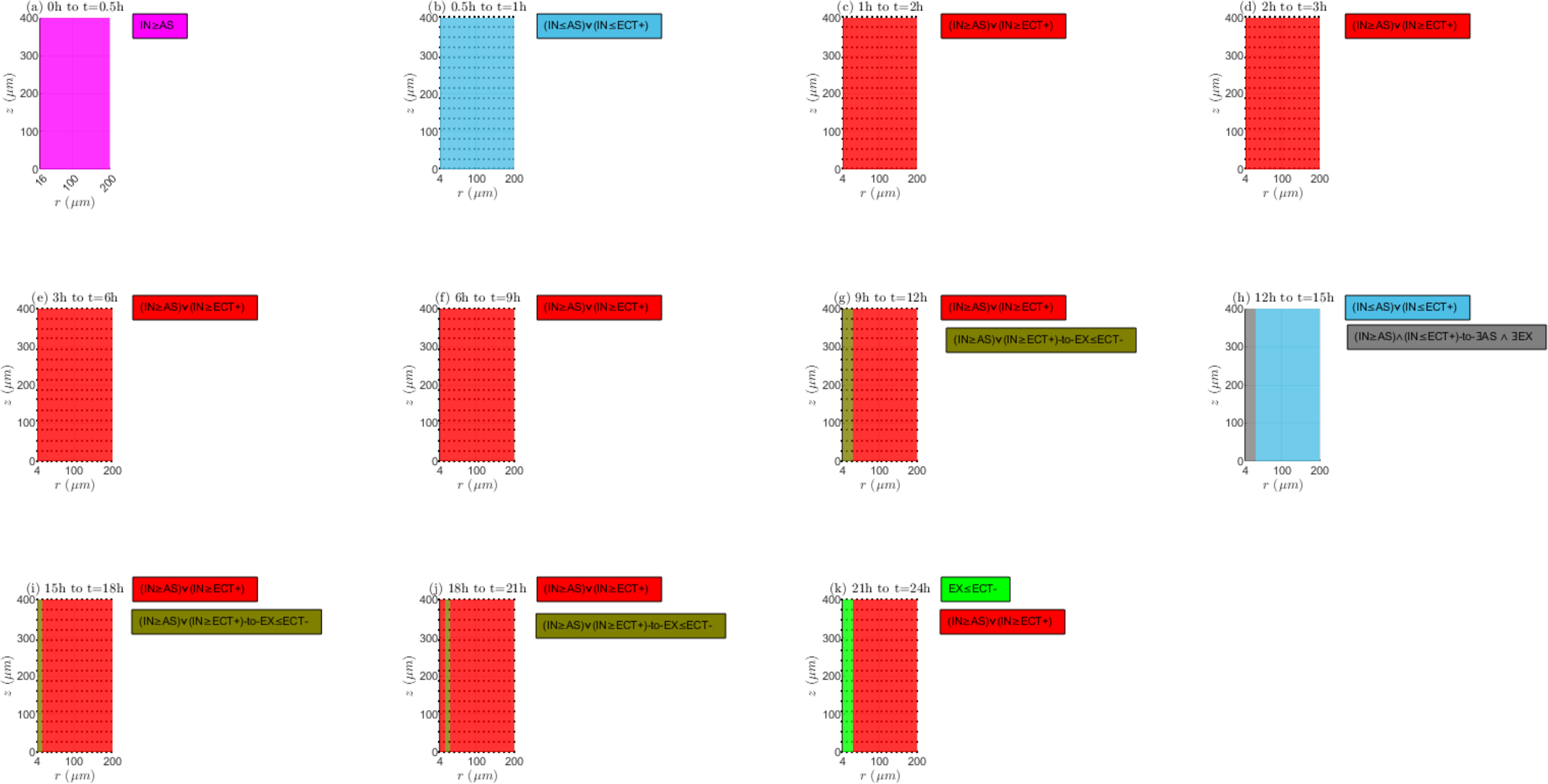

Supplement: S28 Fig — (TIF) [file pone.0315194.s028.tif]

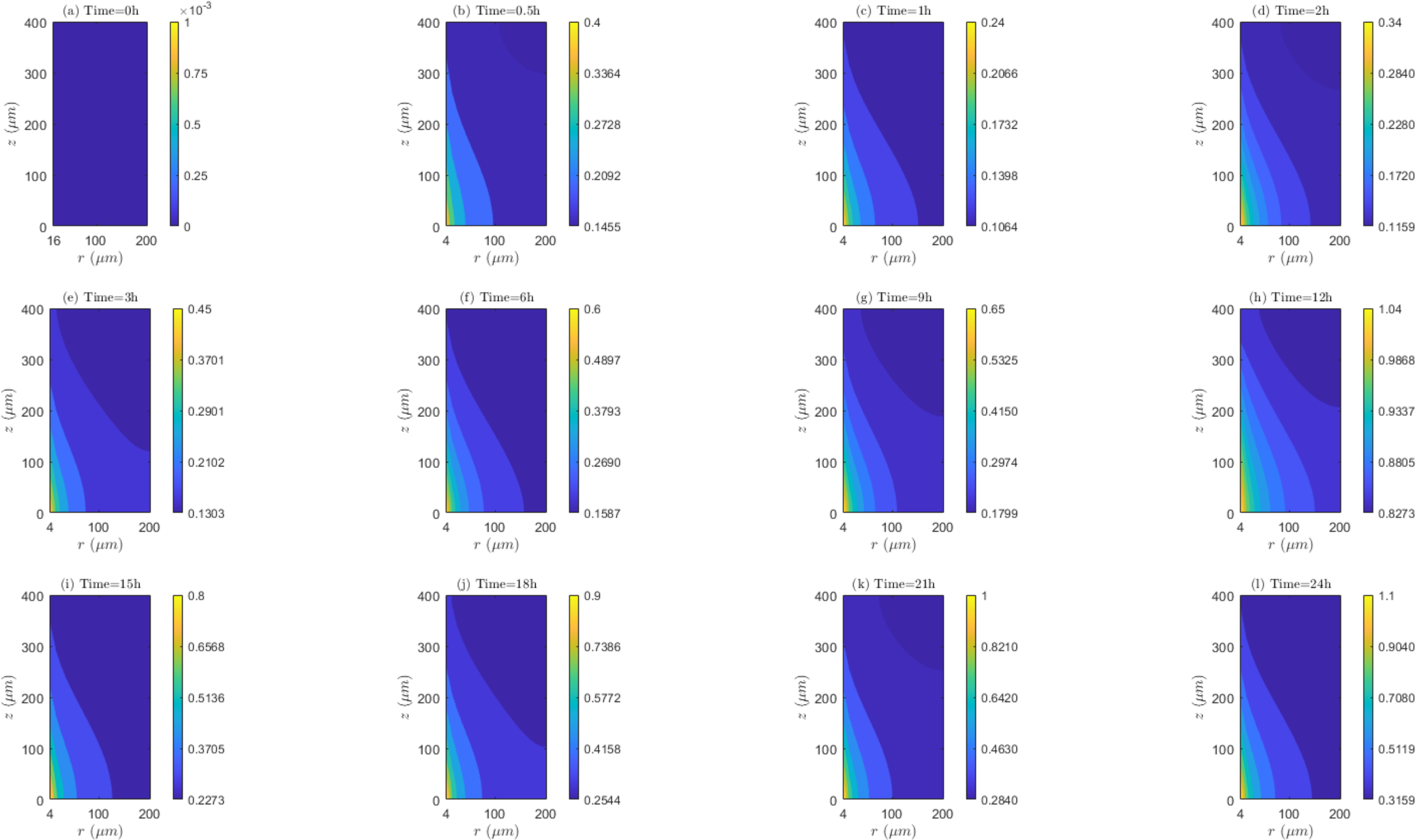

Supplement: S29 Fig — (TIF) [file pone.0315194.s029.tif]

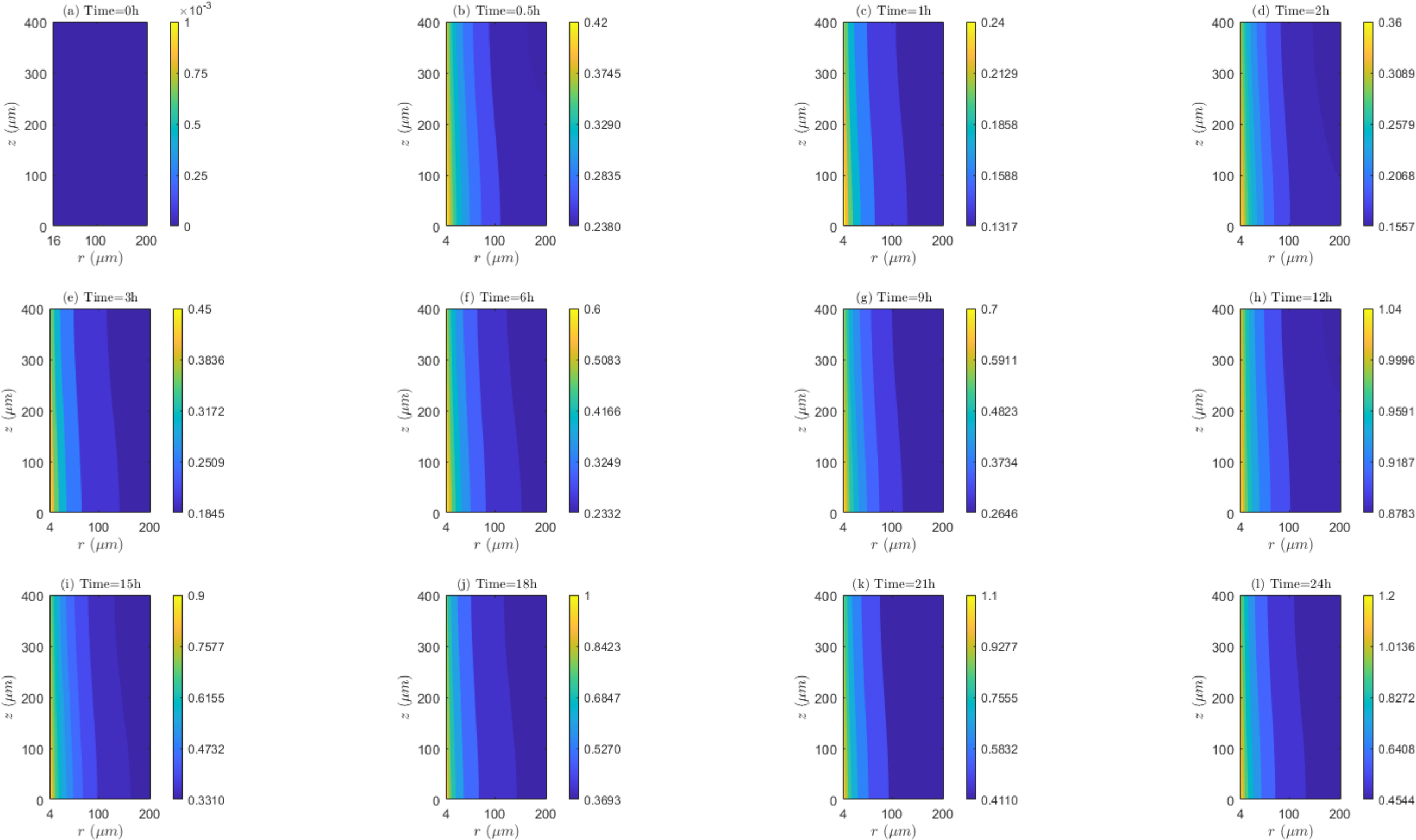

Supplement: S30 Fig — (TIF) [file pone.0315194.s030.tif]

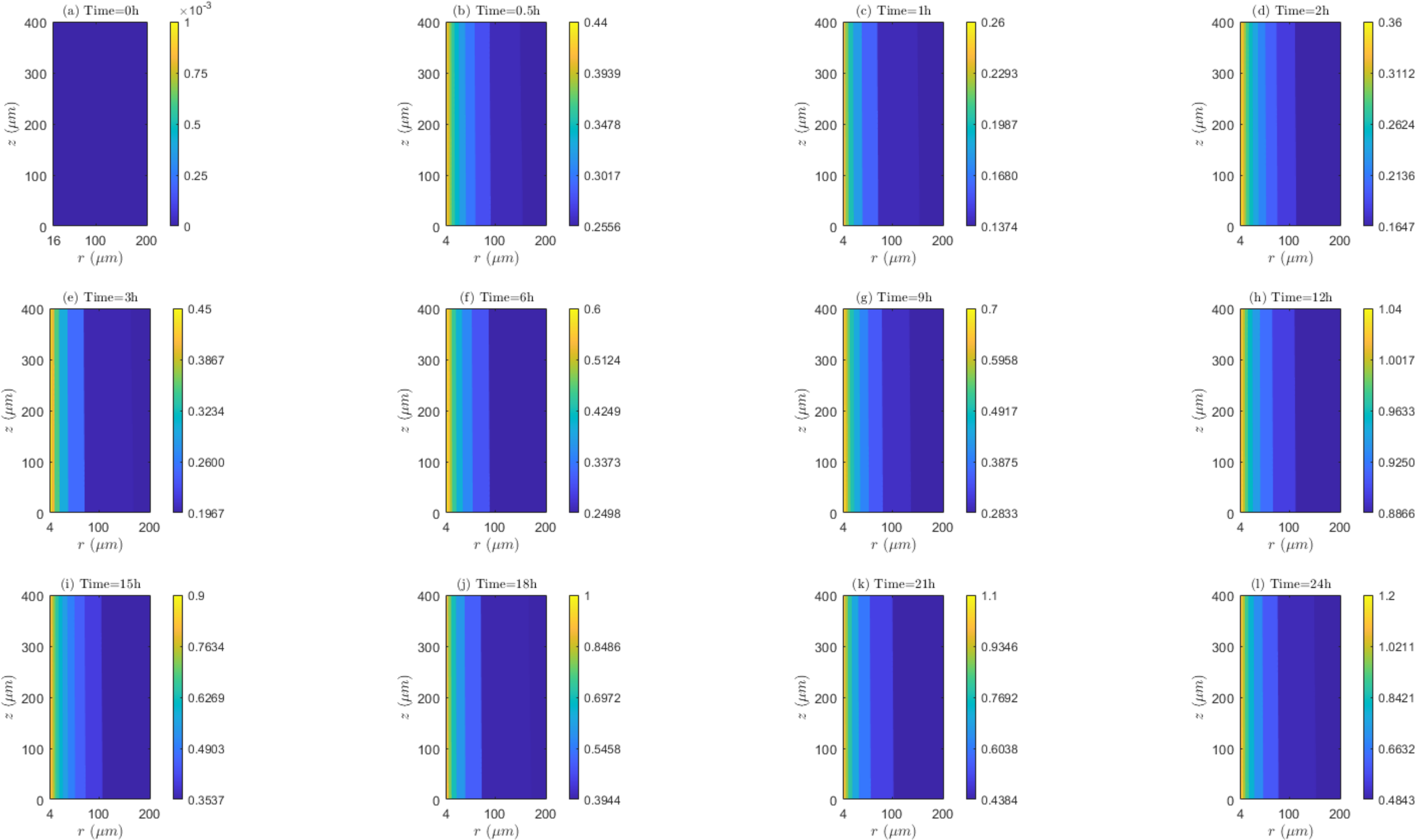

Supplement: S31 Fig — (TIF) [file pone.0315194.s031.tif]

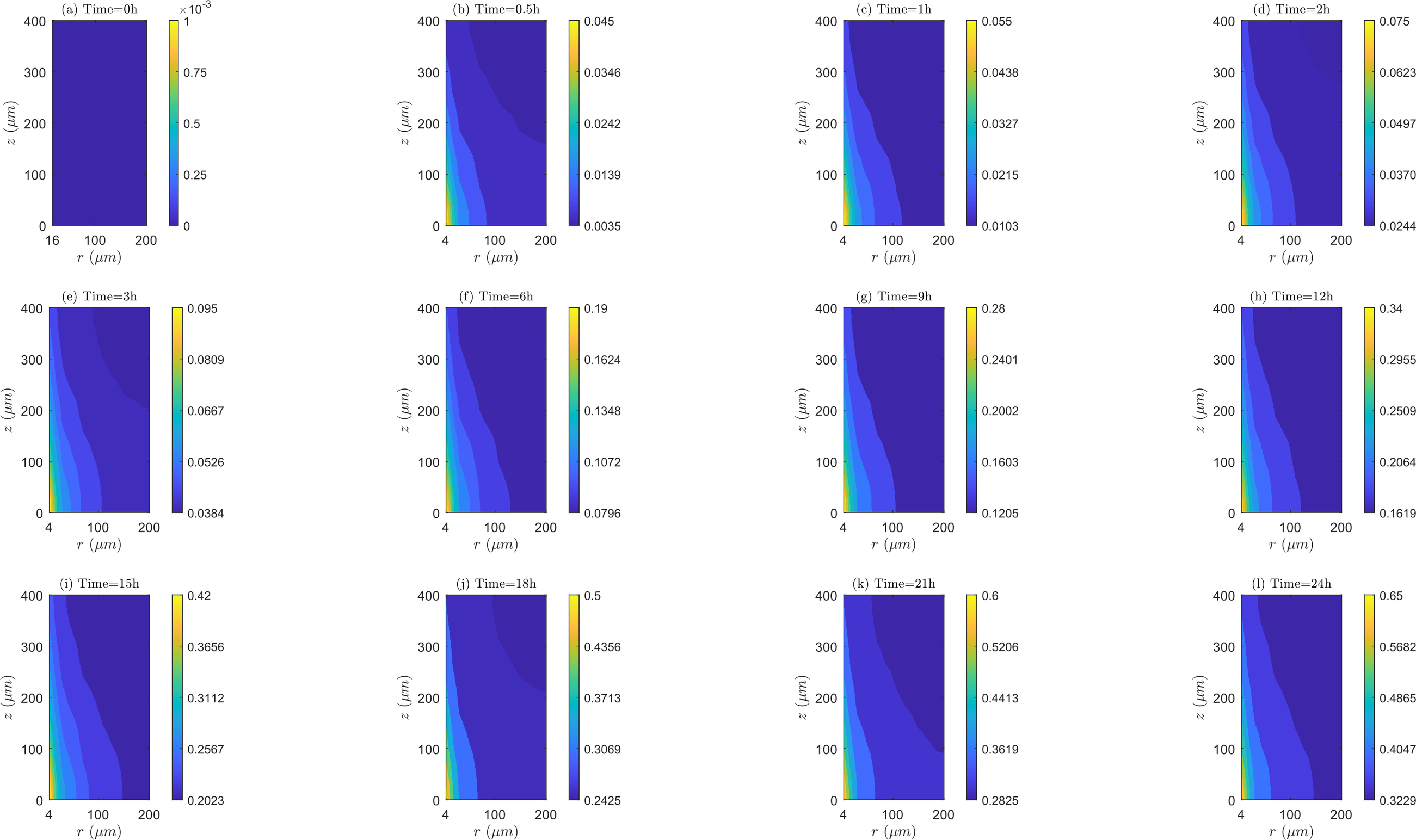

Supplement: S32 Fig — (TIF) [file pone.0315194.s032.tif]

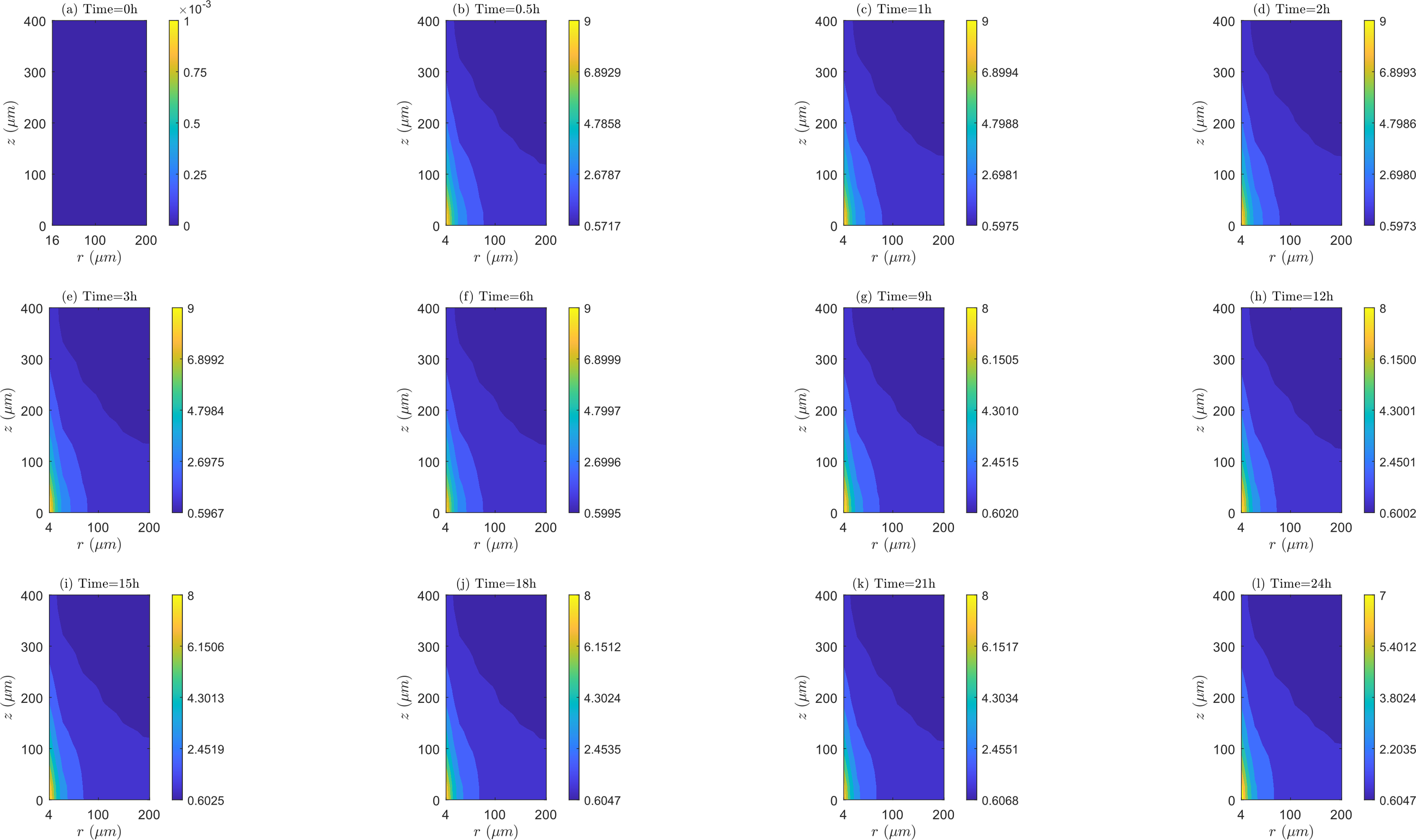

Supplement: S33 Fig — (TIF) [file pone.0315194.s033.tif]

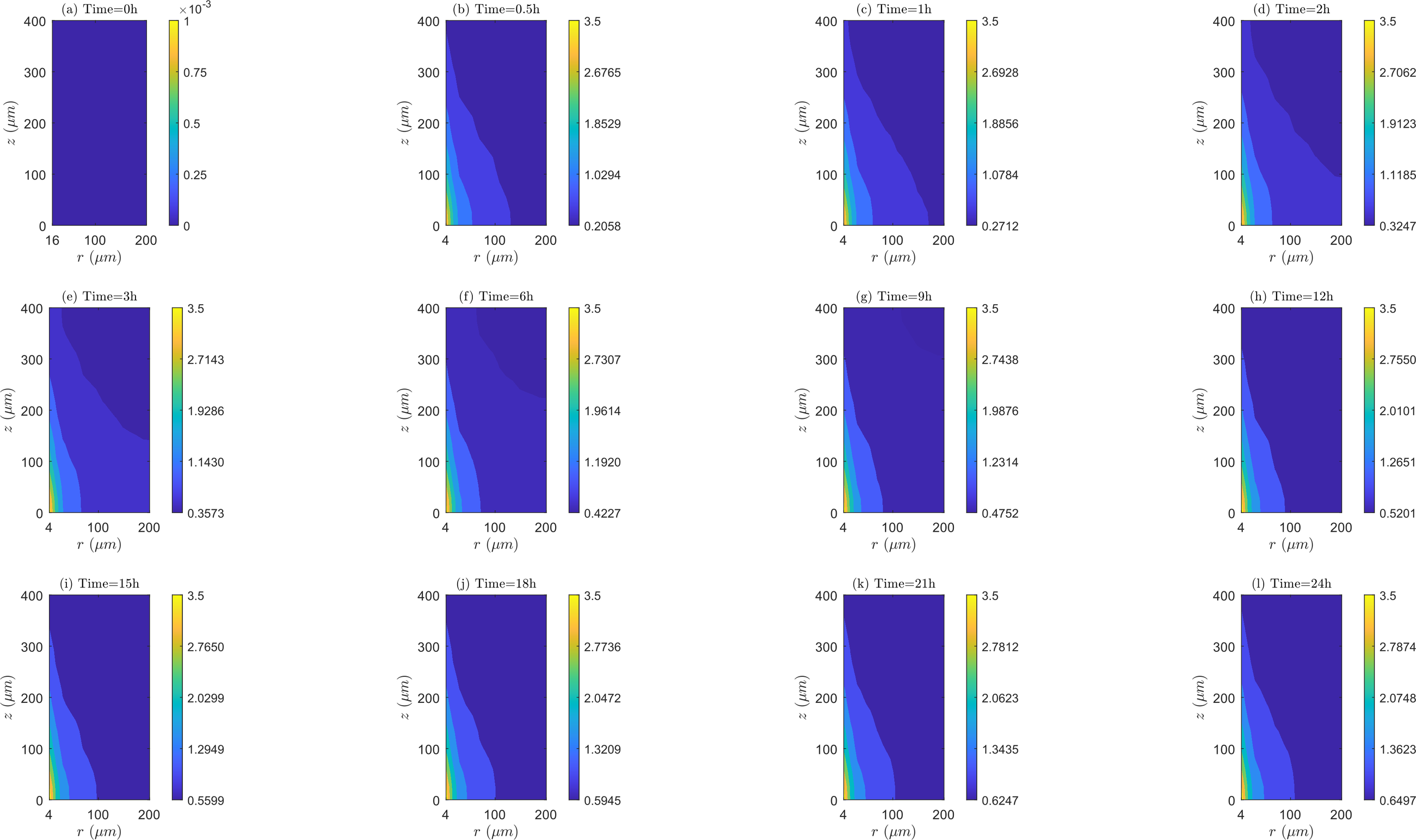

Supplement: S34 Fig — (TIF) [file pone.0315194.s034.tif]

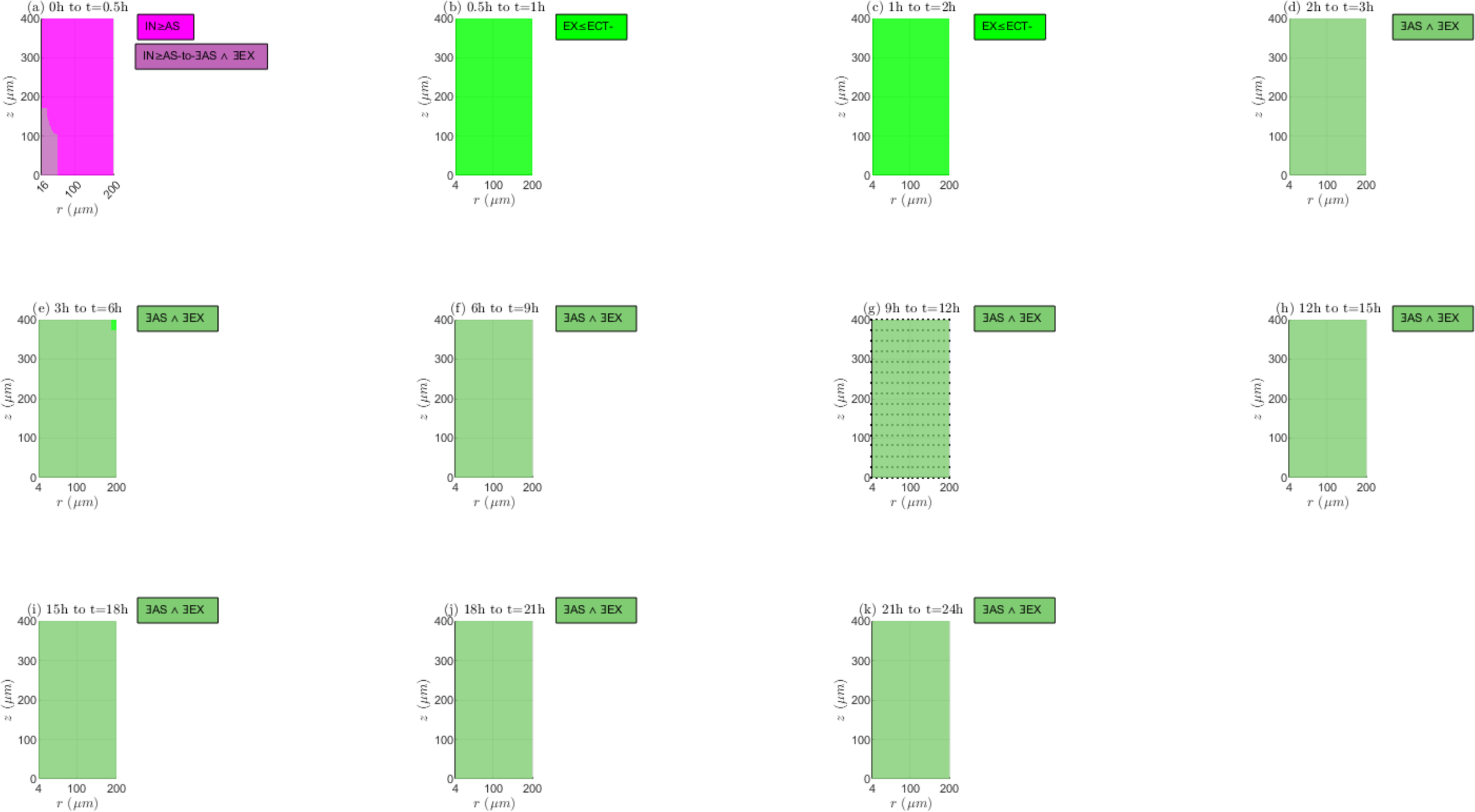

Supplement: S35 Fig — (TIF) [file pone.0315194.s035.tif]

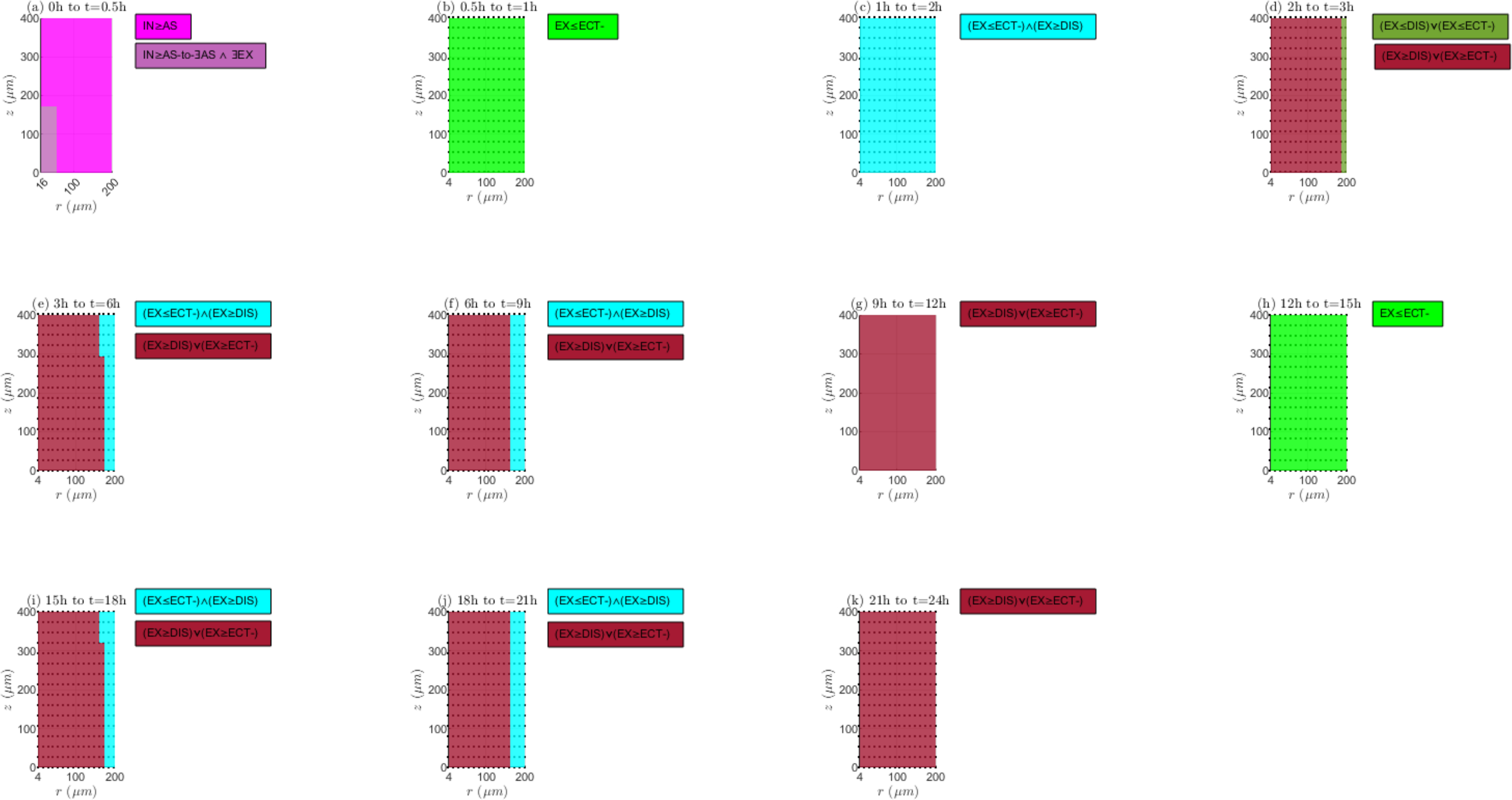

Supplement: S36 Fig — (TIF) [file pone.0315194.s036.tif]

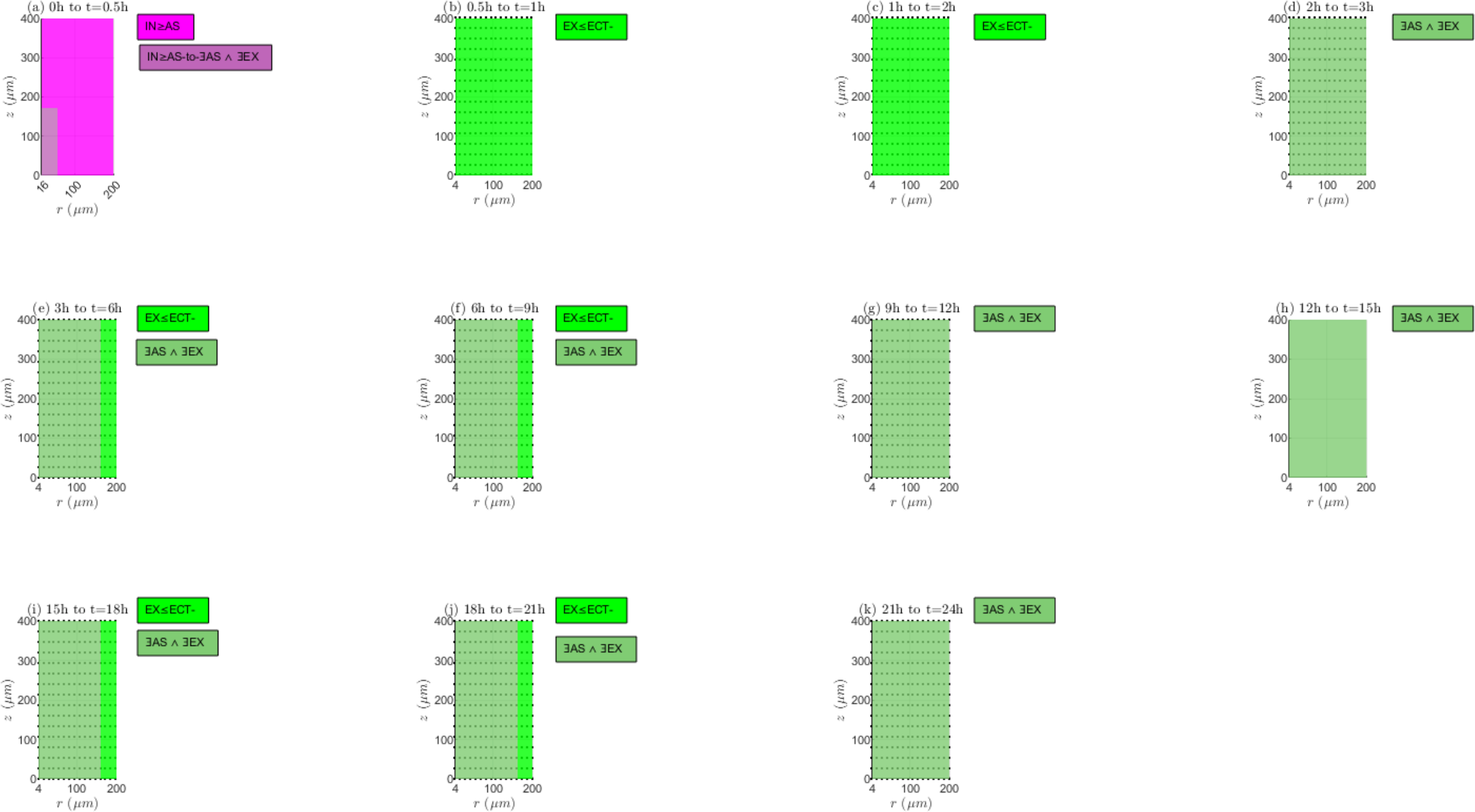

Supplement: S37 Fig — (TIF) [file pone.0315194.s037.tif]

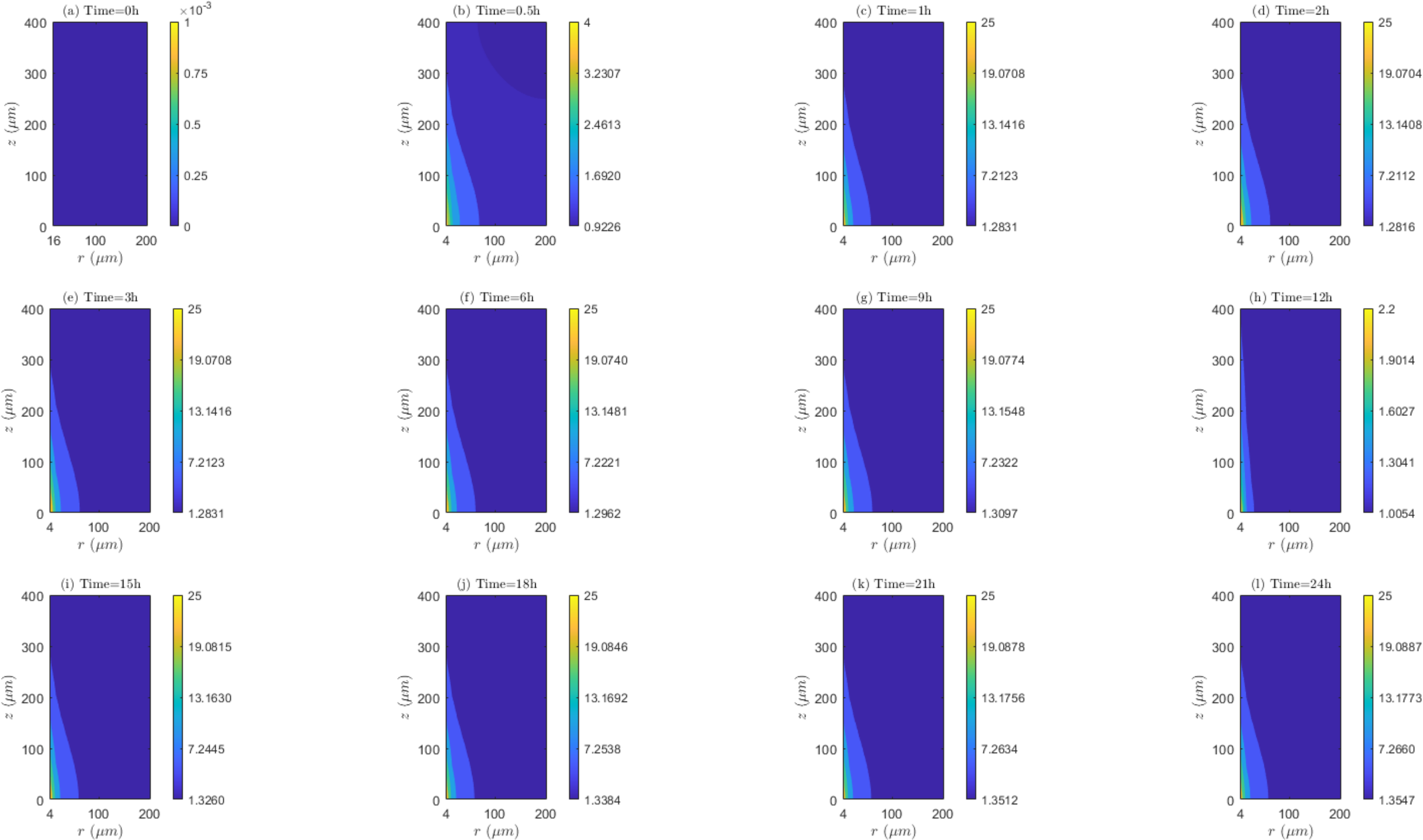

Supplement: S38 Fig — (TIF) [file pone.0315194.s038.tif]

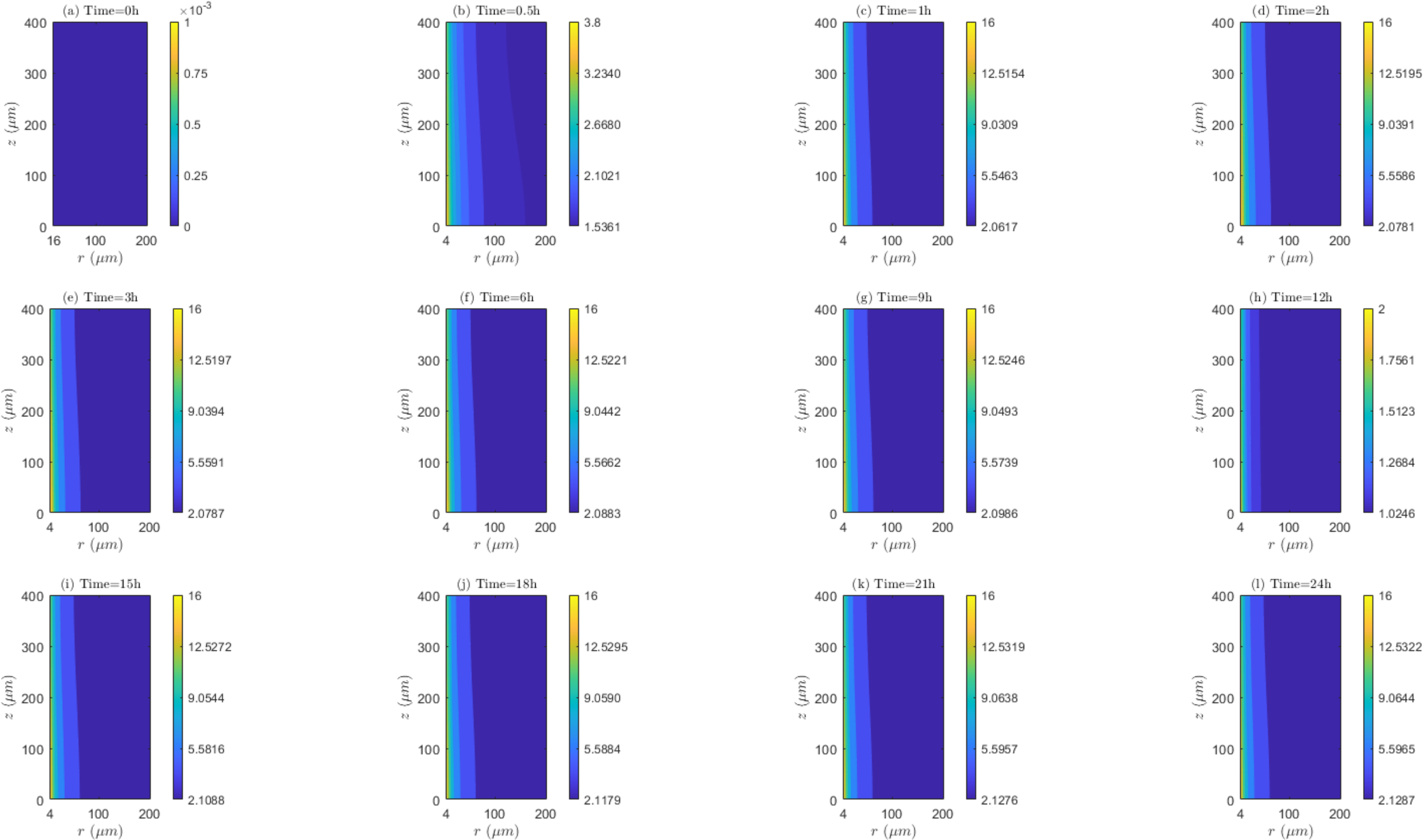

Supplement: S39 Fig — (TIF) [file pone.0315194.s039.tif]

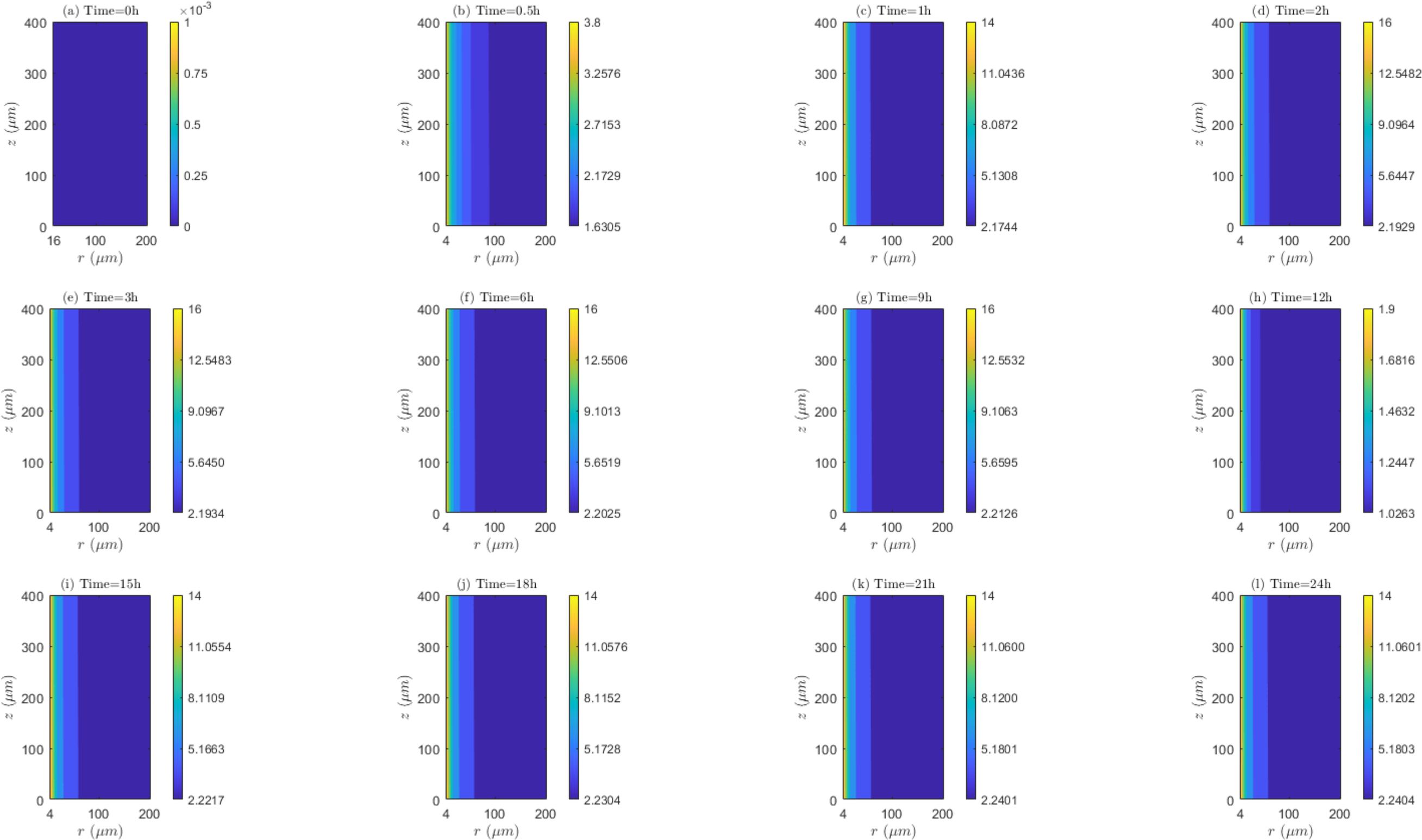

Supplement: S40 Fig — (TIF) [file pone.0315194.s040.tif]

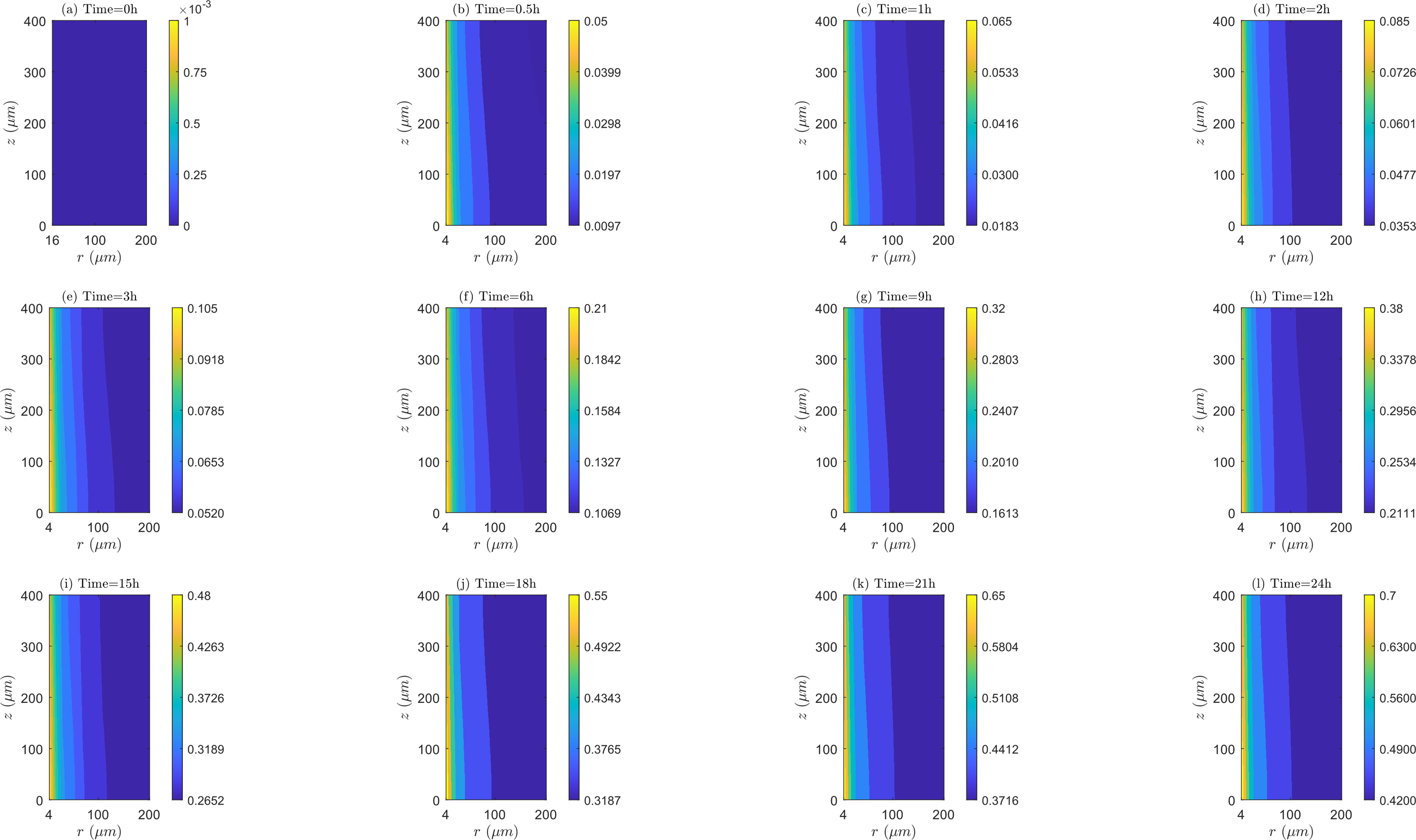

Supplement: S41 Fig — (TIF) [file pone.0315194.s041.tif]

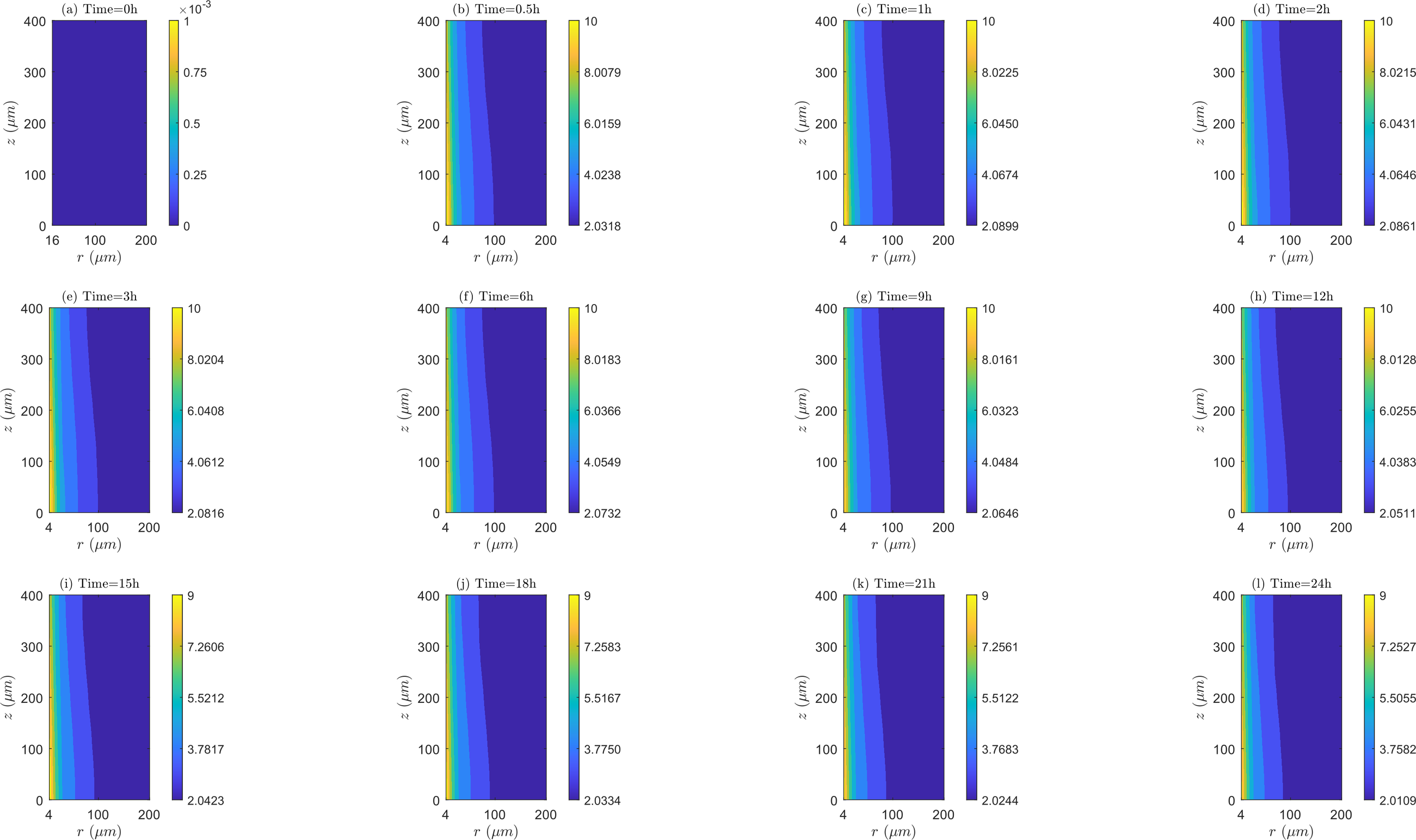

Supplement: S42 Fig — (TIF) [file pone.0315194.s042.tif]

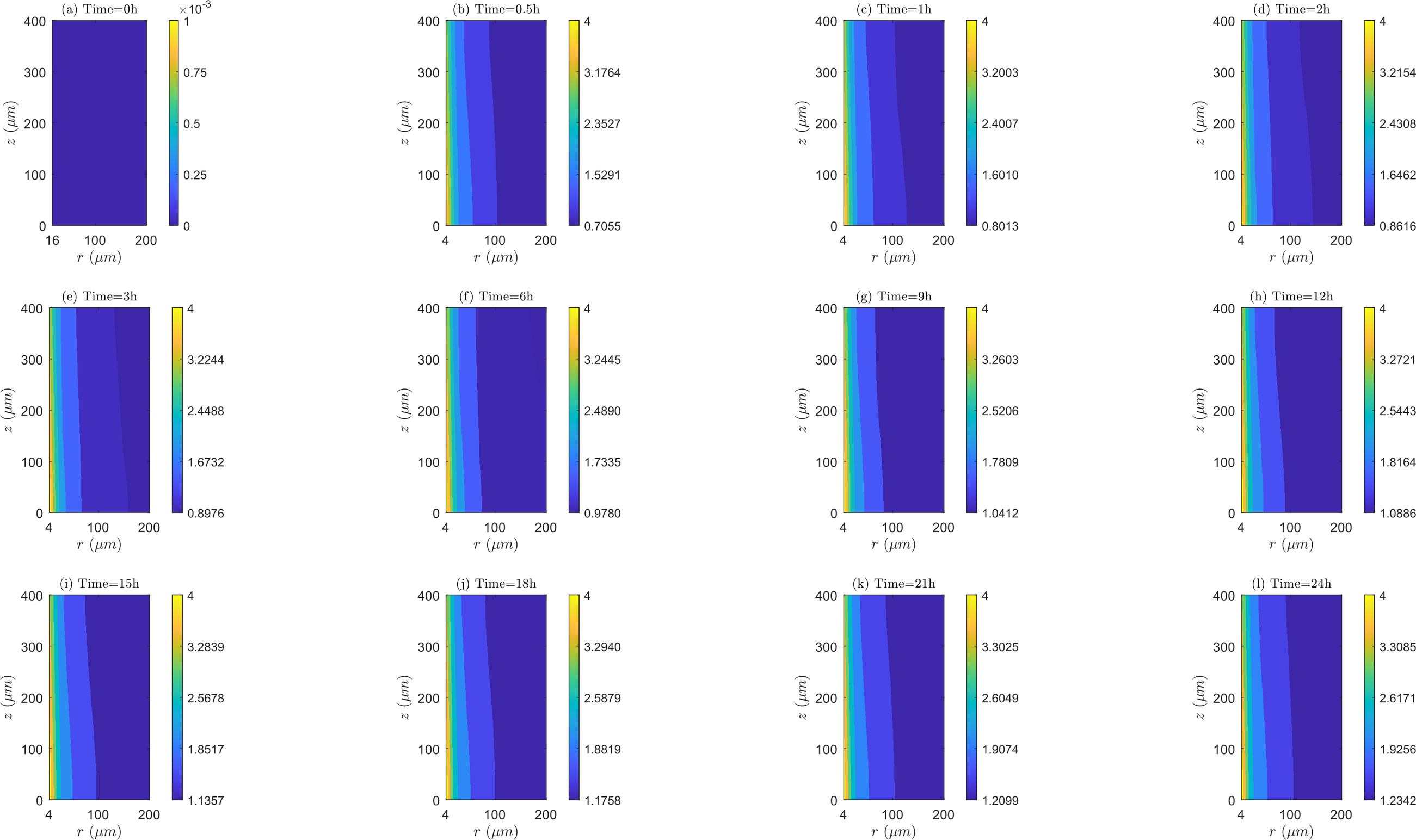

Supplement: S43 Fig — (TIF) [file pone.0315194.s043.tif]
