# Supplementary material for: In-silico tool based on Boolean networks and meshless simulations for prediction of reaction and transport mechanisms in the systemic administration of chemotherapeutic drugs
Source: PLoS One. 2025 Feb 7;20(2):e0315194. doi: 10.1371/journal.pone.0315194 (PMC11805580; doi:10.1371/journal.pone.0315194)
Supplement: S1 File — (ZIP) [file pone.0315194.s044.zip › Art 7 - SM - 06_11_2024 - Last.docx]

***SUPPLEMENTARY MATERIAL.***

1. ***Description of Transport and Reaction Mechanisms (***$\boldsymbol{RTMs}$***).***

***Transport along the blood vessels* (**$\boldsymbol{TBV}$**):** The administration of chemotherapeutic drugs is carried out systemically via the primary circulatory system, whereby drug passes to the tumor microvasculature. The primary mechanism of drug transportation along tumor vessels is convection, and the spatial and temporal changes in drug concentration within the vessels are influenced by diverse factors, including the structure of the microvascular network [1], [2], [3], shear stress on the vessel walls [4], initial dose [5], intravascular pressure [4], leakage flow rate across the vessel walls [6], [7], [8], protein binding [5], [9], vasoconstriction and subsequent recovery of vessel diameter [10], [11], [12], [13], [14], [15], vascular endothelial growth factor [16], [17], vessel tortuosity [18], blood hypoxia and acidosis [24], [25], [26], presence of anatomical and/or functional shunts [27], [28], bloodstream velocity ($\lambda$) [5], [17], [29], pharmacokinetic profile of the drug ($PK$) [12], among other factors. A parametric study considering the last two mentioned factors ($PK$ and $\lambda$) is conducted here for electrically-stimulated blood vessels. The convective transport within blood vessels is modelled here using an advection/convection equation, which is based on an established model for fluid flow through porous circular tubes, and incorporates a previously published model from [7] to address the vessel vasoconstriction and subsequent recovery.

***Transvascular transport (***$\boldsymbol{TVT}$***):*** Extravasation is the process by which drug moves from the blood vessel into the interstitial space of the surrounding tissue. Conversely, drainage refers to the movement of substances from the extracellular space back into the lymphatic vessels. Both convection and passive diffusion transport are observed in both situations, whereas transcytosis is typically disregarded in tumor tissues. When examining the convective and diffusive transport through the vessel wall individually, the Staverman-Kedem-Katchalsky equation, in conjunction with Starling's law for transvascular exchange, can be employed [30], [31]. These equations are in terms of the hydraulic conductivity, diffusional permeability, osmotic reflection coefficient of the blood and lymphatic vessel walls, differences in osmotic and hydrostatic pressure between plasma and interstitial space, intra-lymphatic pressure, among others. A more simplified approach consists of a Fickian diffusion equation that incorporates an effective vessel wall permeability and is valid when the transvascular transport is mainly driven by the species concentration gradients rather than by the pressure gradient across the vessel wall [12], [32], [33]. In the present study, the transvascular transport is denoted as $TVT+$ or $EV$ when it occurs from the vessel to the interstitial space (extravasation), and as $TVT-$ or $LD$ when it occurs in the opposite direction (lymphatic drainage). As indicated in previous experimental studies [4], [18], [34], the electric pulses application has been shown to result in the permeabilization of the endothelial cell membrane and the disruption of cell-to-cell junctions, facilitating this way the $TVT$, which in turns can lead to an improvement in the effectiveness of the therapy.

***Extracellular transport (***$\boldsymbol{ECT}$***):*** this relies on the transport mechanisms of convection and diffusion inside the extracellular space, where protein-drug interactions can also take place, resulting in the inhibition of drug transportation and influencing the process of drug absorption into cells. The outcome of these interactions depends on the specific characteristics of both the drug and the protein involved. Additionally, it should be noted that the extracellular porosity and tumor vascular network may exhibit significant non-homogeneity, particularly in cases where tumor and healthy tissues are significantly intermixed. In the context of macroscopic analysis, several equations may be considered to account for mass conservation, fluid flow momentum, and species transport in the interstitial space, with the incorporation of various terms to represent the source (vascular extravasation) and sink (lymphatic drainage) of mass, the resistance to flow within the tissue (Darcian resistance), protein association/dissociation, and influx/efflux effects, without explicitly representing the vasculature of the tumor [31], [35], [36], [37]. In contrast, the inclusion of the intricate vasculature network at a microscopic level poses a significant computational burden [38], [39], [40], [41], [42]. Nevertheless, the tumor cord approach offers a simplified and idealized depiction of the tumor vasculature, enabling the assessment of the drug penetration from a blood vessel to the less perfused regions within the tumor [7], [12], [13], [14], [32], [33]. In the original tumor cord tumor model, it is assumed that the dominant mode of drug transport in the extracellular space is diffusion [12], [32], [33]. A modified tumor cord model that incorporates a convective term to account for the volume change of the tissue when vasoconstriction occurs was used in Vélez et al. [7] and is taken up here again. In the current study, the term $ECT+$ is used to denote a positive net extracellular transport in the Representative Unitary Cell ($RUC$), indicating an increase in the extracellular concentration $(C_{1})$ between two time-instants. Conversely, $ECT-$ refers to a negative net extracellular transport, signifying a decrease in $C_{1}$ at a given $RUC$. Previous researches have examined the impact of electric pulse application on $ECT$, coinciding in the enhancement of the interstitial diffusion for both nanoparticles [34], chemotherapeutic drug [43], [44], [45] and DNA [46] due to the reduction of the interstitial barrier of the extracellular matrix ($ECM$). The balance between the electro-permeabilization of the extracellular space and cell membrane has been studied both *in vivo* [47], [48] and *in vitro* [49], [50], employing a combination of high voltage, short pulses, and low voltage, long pulses.

***Transmembrane transport (***$\boldsymbol{TMT}$***):*** this encompasses the processes of drug passage inward and outward the cell, which can occur concurrently and primarily involve four distinct mechanisms of transport: (1) passive diffusion that occurs due to concentration gradient differences, (2) transport facilitated by gated protein channels, (3) active transport by means of carrier proteins utilizing metabolic energy from $ATP$ or other sources, (4) endocytosis-exocytosis. In the tumor cord approach used here the transmembrane transport is incorporated by source/sink terms into the species transport equations for extracellular concentration ($C_{1}$) and free intracellular concentration ($C_{2}$), taking into account the effective permeability of the cell membrane ($K_{1}$) modified by a function known as the degree of reversible electroporation (${DOE}_{R}$) proposed by Boyd and Becker [15]. Given that cellular uptake and efflux of drug can occur simultaneously inside the same $RUC$, the net rate of transmembrane transport is considered here. In this context, the terms $TMT+$ or $INT$ denote a net internalization of drug, whereas $TMT-$ or $EX$ indicate a net externalization. The impact of electroporation ($EP$) on transmembrane transport has been investigated in many experimental studies [51], [52], [53], [54], [55] as well as in numerical simulations [7], [13], [14], [15], [56], [57], [58]. The primary factors contributing to the electro-permeabilization of the cell membrane, as described by Sachdev et al. [46], include the creation of hydrophilic pores within lipid bilayers, the alteration of cell membrane structure due to chemical modifications of lipid tails, and the denaturation of membrane proteins resulting in the generation of voltage gated ion-channels. In certain macromolecules, such as DNA, the process of translocation towards the interior of the cell may involve an initial aggregation on the cell surface [59], [60].

The cellular membrane integrity is a critical factor that demands careful control during the application of electric pulses because when irreversible electroporation ($IRE$) takes place, a portion of the cell membrane pores stay unsealed, resulting in the rupture of some regions and subsequently causing a chemical imbalance [61]. This can lead to side effects on the tissue such as hemorrhage, portal vein thrombosis, bile duct injury, and infection [62], [63]. The most of in-silico investigations focused on simulating transmembrane transport ($TMT$) at both macroscopic and microscopic scales have employed conventional numerical methods such as the Finite Element Method ($FEM$) and the Finite Volume Method ($FVM$). The Finite Volume Method ($FVM$) was utilized in [15], [56], [64] to examine the impact of voltage level, pulse spacing, electrodes configuration, and initial drug concentration on the efficacy and cell survival rate of electro-chemotherapeutic treatments. Šel et al. [57] employed $FEM$ to calculate the distribution of electric field and tissue conductivity during electroporation, and their findings were validated with in-vivo measurements on rabbit liver tissues. The aforementioned authors [57] also used $FEM$ to investigate the increase of cell membrane permeability in subcutaneous tumors in mice. Additionally, Shirakashi et al. [65] employed $FEM$ to examine the electro-permeabilization in mouse skin tissues, taking into account various electrode configurations and electric protocols implemented in in-vivo experiments. Furthermore, the use of commercial software has been also implemented in numerical simulations of cell membrane permeabilization [66]. The Global Method of Approximate Particular Solutions ($GMAPS$) was recently used to study the spatio-temporal behavior of extracellular and intracellular drug concentration in electroporated tissues. In their study, Vélez et al. [67] employed this method to investigate the impact of voltage level ($V$) and pulse spacing ($d_{pulses}$) on both the quantity and uniformity of the internalized drug. Additionally, Vélez et al. [58] examined the effects of several chemotherapeutic drugs, specifically cisplatin and doxorubicin, on the $TMT$ mechanism. Puc et al. [68] conducted a study to evaluate the impact of voltage level, pulse duration and elapsed time on the transmembrane transport of medications using a two-compartment model, obtaining mathematical expressions for the effective mass transfer coefficient between the intracellular and extracellular spaces.

***Intracellular transport (***$\boldsymbol{ICT}$***):*** this refers to the process by which macromolecules traverse the cytoplasm to reach the nuclear envelope. The cytoplasm is a highly concentrated fluid that primarily consists of a network of cytoskeleton, diverse cellular organelles, and abundant proteins. In this context, the convective and diffusive transports are typically disregarded [46]. Within the intracellular environment, the processes of drug association ($AS$) and dissociation $(DIS)$ with proteins play a significant role in determining the free intracellular ($C_{2}$) and bound intracellular concentrations ($C_{3}$). In the tumor cord model employed in this study, the aforementioned reaction mechanisms are incorporated into the species transport equations as source/sink factors, depending on the drug association ($K_{2}$) and dissociation rates ($K_{-2}$). The present study does not take into account the impact of electric pulse application on $K_{2}$ and $K_{-2}$.

**References.**

1. Winkler F, Kozin S V., Tong RT, Chae SS, Booth MF, Garkavtsev I, et al. Kinetics of vascular normalization by VEGFR2 blockade governs brain tumor response to radiation. Cancer Cell. 2004;6(6).

2. Jain RK. Normalization of tumor vasculature: An emerging concept in antiangiogenic therapy. Vol. 307, Science. 2005.

3. Secomb TW, Hsu R, Dewhirst MW, Klitzman B, Gross JF. Analysis of oxygen transport to tumor tissue by microvascular networks. Int J Radiat Oncol Biol Phys. 1993;25(3).

4. Pries AR. Structural adaptation and stability of microvascular networks: Theory and simulations. Am J Physiol Heart Circ Physiol. 1998;275(2 44-2).

5. Hubbard ME, Jove M, Loadman PM, Phillips RM, Twelves CJ, Smye SW. Drug delivery in a tumour cord model: a computational simulation. R Soc Open Sci [Internet]. 2017 May 24;4(5):170014. Available from: https://royalsocietypublishing.org/doi/10.1098/rsos.170014

6. Vélez Salazar FM, Patiño Arcila ID. Influence of electric field, blood velocity, and pharmacokinetics on electrochemotherapy efficiency. Biophys J. 2023;122(16).

7. Salazar FMV, Arcila IDP. In silico study about the influence of electroporation parameters on the cellular internalization, spatial uniformity, and cytotoxic effects of chemotherapeutic drugs using the Method of Fundamental Solutions. Med Biol Eng Comput. 2023;

8. Vélez Salazar FM, Patiño Arcila ID. Influence of electric pulse characteristics on the cellular internalization of chemotherapeutic drugs and cell survival fraction in electroporated and vasoconstricted cancer tissues using boundary element techniques. J Math Biol [Internet]. 2023 Aug 18;87(2):31. Available from: https://link.springer.com/10.1007/s00285-023-01963-z

9. Boyd B, Becker S. Macroscopic Modeling of In Vivo Drug Transport in Electroporated Tissue. J Biomech Eng [Internet]. 2016 Mar 1;138(3):1–12. Available from: https://asmedigitalcollection.asme.org/biomechanical/article/doi/10.1115/1.4032380/370470/Macroscopic-Modeling-of-In-Vivo-Drug-Transport-in

10. Brinton M, Mandel Y, Schachar I, Palanker D. Mechanisms of electrical vasoconstriction. J Neuroeng Rehabil. 2018;15(1):1–10.

11. Palanker D, Vankov A, Freyvert Y, Huie P. Pulsed electrical stimulation for control of vasculature: Temporary vasoconstriction and permanent thrombosis. Bioelectromagnetics. 2008;29(2):100–7.

12. Mandel Y, Manivanh R, Dalal R, Huie P, Wang J, Brinton M, et al. Vasoconstriction by Electrical Stimulation: New Approach to Control of Non-Compressible Hemorrhage. Sci Rep [Internet]. 2013 Dec 4;3(1):2111. Available from: http://www.nature.com/articles/srep02111

13. Corovic S, Markelc B, Dolinar M, Cemazar M, Jarm T. Modeling of microvascular permeability changes after electroporation. Schneditz D, editor. PLoS One [Internet]. 2015 Mar 20;10(3):e0121370. Available from: https://dx.plos.org/10.1371/journal.pone.0121370

14. Markelc B, Sersa G, Cemazar M. Differential Mechanisms Associated with Vascular Disrupting Action of Electrochemotherapy: Intravital Microscopy on the Level of Single Normal and Tumor Blood Vessels. PLoS One. 2013;8(3):1–11.

15. Serša G, Čemažar M, Markelc B. Blood Flow Modifying and Vascular-Disrupting Effects of Electroporation and Electrochemotherapy. In: Handbook of Electroporation [Internet]. Cham: Springer International Publishing; 2017 [cited 2019 Jul 9]. p. 691–705. Available from: http://link.springer.com/10.1007/978-3-319-32886-7_165

16. Buchanan CF, Verbridge SS, Vlachos PP, Rylander MN. Flow shear stress regulates endothelial barrier function and expression of angiogenic factors in a 3D microfluidic tumor vascular model. Cell Adh Migr. 2014;8(5).

17. Dewhirst MW, Ashcraft KA. Implications of increase in vascular permeability in tumors by VEGF: A commentary on the pioneering work of Harold Dvorak. Vol. 76, Cancer Research. 2016.

18. Sevick EM, Jain RK. Geometric Resistance to Blood Flow in Solid Tumors Perfused ex Vivo:Effects of Tumor Size and Perfusion Pressure. Cancer Res. 1989;49(13).

19. Dewhirst MW, Ong ET, Madwed D, Klitzman B, Secomb T, Brizel D, et al. Effects of the calcium channel blocker flunarizine on the hemodynamics and oxygenation of tumor microvasculature. Radiat Res. 1992;132(1).

20. Kavanagh BD, Coffey BE, Needham D, Hochmuth RM, Dewhirst MW. The effect of flunarizine on erythrocyte suspension viscosity under conditions of extreme hypoxia, low ph, and lactate treatment. Br J Cancer. 1993;67(4).

21. Dewhirst MW, Ong ET, Braun RD, Smith B, Klitzman B, Evans SM, et al. Quantification of longitudinal tissue pO2 gradients in window chamber tumours: Impact on tumour hypoxia. Br J Cancer. 1999;79(11–12).

22. Sorg BS, Hardee ME, Agarwal N, Moeller BJ, Dewhirst MW. Spectral imaging facilitates visualization and measurements of unstable and abnormal microvascular oxygen transport in tumors. J Biomed Opt. 2008;13(1).

23. Yeh C, Hu S, Liang J, Li L, Soetikno B, Lu ZH, et al. Optical-resolution photoacoustic microscopy of the metabolic rate of oxygen in a mouse renal tumor model. In: Photons Plus Ultrasound: Imaging and Sensing 2015. 2015.

24. Bellard E, Markelc B, Pelofy S, Le Guerroué F, Sersa G, Teissié J, et al. Intravital microscopy at the single vessel level brings new insights of vascular modification mechanisms induced by electropermeabilization. Journal of Controlled Release [Internet]. 2012;163(3):396–403. Available from: http://dx.doi.org/10.1016/j.jconrel.2012.09.010

25. Lv J, Cao J feng, Cai Y, Zhou Y, Long Q, Yao W, et al. Numerical Simulation of Solid Tumor Blood Perfusion and Drug Delivery during the “Vascular Normalization Window” with Antiangiogenic Therapy. J Appl Math [Internet]. 2011;2011:1–8. Available from: http://www.hindawi.com/journals/jam/2011/190371/

26. Jain RK. Transport of molecules across tumor vasculature. CANCER AND METASTASIS REVIEW. 1987;6(4).

27. Zhan W, Gedroyc W, Xu X. Mathematical Modelling of Drug Transport and Uptake in a Realistic Model of Solid Tumour. Protein Pept Lett. 2014 Oct 3;21(11):1146–56.

28. Eikenberry S. A tumor cord model for Doxorubicin delivery and dose optimization in solid tumors. Theor Biol Med Model [Internet]. 2009 Dec 9;6(1):16. Available from: https://tbiomed.biomedcentral.com/articles/10.1186/1742-4682-6-16

29. Groh CM, Hubbard ME, Jones PF, Loadman PM, Periasamy N, Sleeman BD, et al. Mathematical and computational models of drug transport in tumours. J R Soc Interface [Internet]. 2014 May 6;11(94):20131173. Available from: https://royalsocietypublishing.org/doi/10.1098/rsif.2013.1173

30. Markelc B, Bellard E, Sersa G, Jesenko T, Pelofy S, Teissié J, et al. Increased permeability of blood vessels after reversible electroporation is facilitated by alterations in endothelial cell-to-cell junctions. Journal of Controlled Release [Internet]. 2018 Apr 28;276(9):30–41. Available from: http://www.ncbi.nlm.nih.gov/pubmed/29476881

31. Kodama H, Shamay Y, Kimura Y, Shah J, Solomon SB, Heller D, et al. Electroporation-induced changes in tumor vasculature and microenvironment can promote the delivery and increase the efficacy of sorafenib nanoparticles. Bioelectrochemistry. 2019;130.

32. Goodman TT, Chen J, Matveev K, Pun SH. Spatio-temporal modeling of nanoparticle delivery to multicellular tumor spheroids. Biotechnol Bioeng. 2008;101(2).

33. Tzafriri AR, Lerner EI, Flashner-Barak M, Hinchcliffe M, Ratner E, Parnas H. Mathematical modeling and optimization of drug delivery from intratumorally injected microspheres. Clinical Cancer Research. 2005;11(2 I).

34. Arifin DY, Lee KYT, Wang CH. Chemotherapeutic drug transport to brain tumor. Journal of Controlled Release. 2009;137(3).

35. Cattaneo L, Zunino P. A computational model of drug delivery through microcirculation to compare different tumor treatments. Int J Numer Method Biomed Eng. 2014;30(11).

36. Secomb T w. A Green’s function method for simulation of time-dependent solute transport and reaction in realistic microvascular geometries. Mathematical Medicine and Biology. 2016;33(4).

37. Stamatelos SK, Kim E, Pathak AP, Popel AS. A bioimage informatics based reconstruction of breast tumor microvasculature with computational blood flow predictions. Microvasc Res. 2014;91.

38. Sefidgar M, Soltani M, Raahemifar K, Sadeghi M, Bazmara H, Bazargan M, et al. Numerical modeling of drug delivery in a dynamic solid tumor microvasculature. Microvasc Res. 2015;99.

39. Stéphanou A, McDougall SR, Anderson ARA, Chaplain MAJ. Mathematical modelling of flow in 2D and 3D vascular networks: Applications to anti-angiogenic and chemotherapeutic drug strategies. Math Comput Model. 2005;41(10).

40. Gibot L, Wasungu L, Teissié J, Rols MP. Antitumor drug delivery in multicellular spheroids by electropermeabilization. Journal of Controlled Release. 2013;167(2).

41. Zhang Z, Li W, Procissi D, Tyler P, Omary RA, Larson AC. Rapid dramatic alterations to the tumor microstructure in pancreatic cancer following irreversible electroporation ablation. Nanomedicine. 2014;9(8).

42. Figini M, Wang X, Lyu T, Su Z, Wang B, Sun C, et al. Diffusion MRI biomarkers predict the outcome of irreversible electroporation in a pancreatic tumor mouse model. Am J Cancer Res. 2018;8(8).

43. Sachdev S, Potočnik T, Rems L, Miklavčič D. Revisiting the role of pulsed electric fields in overcoming the barriers to in vivo gene electrotransfer. Vol. 144, Bioelectrochemistry. 2022.

44. Satkauskas S, Bureau MF, Puc M, Mahfoudi A, Scherman D, Miklavcic D, et al. Mechanisms of in vivo DNA electrotransfer: Respective contribution of cell electropermeabilization and DNA electrophoresis. Molecular Therapy. 2002;5(2).

45. Bureau MF, Gehl J, Deleuze V, Mir LM, Scherman D. Importance of association between permeabilization and electrophoretic forces for intramuscular DNA electrotransfer. Biochim Biophys Acta Gen Subj. 2000;1474(3).

46. Sukharev SI, Klenchin VA, Serov SM, Chernomordik L V., Chizmadzhev YuA. Electroporation and electrophoretic DNA transfer into cells. The effect of DNA interaction with electropores. Biophys J. 1992;63(5).

47. Pavšelj N, Préat V. DNA electrotransfer into the skin using a combination of one high- and one low-voltage pulse. Journal of Controlled Release. 2005;106(3).

48. Batista Napotnik T, Miklavčič D. In vitro electroporation detection methods – An overview. Bioelectrochemistry. 2018 Apr;120:166–82.

49. Kotnik T, Rems L, Tarek M, Miklavčič D. Membrane Electroporation and Electropermeabilization: Mechanisms and Models. Annu Rev Biophys. 2019 May 6;48(1):63–91.

50. Rems L, Miklavčič D. Tutorial: Electroporation of cells in complex materials and tissue. J Appl Phys. 2016 May 28;119(20):201101.

51. Sweeney DC, Douglas TA, Davalos R V. Characterization of Cell Membrane Permeability In Vitro Part II: Computational Model of Electroporation-Mediated Membrane Transport*. Technol Cancer Res Treat. 2018 Jan 19;17:153303381879249.

52. Sweeney DC, Weaver JC, Davalos R V. Characterization of Cell Membrane Permeability In Vitro Part I: Transport Behavior Induced by Single-Pulse Electric Fields*. Technol Cancer Res Treat. 2018 Jan 20;17:153303381879249.

53. Argus F, Boyd B, Becker SM. Electroporation of tissue and cells: A three-equation model of drug delivery. Comput Biol Med [Internet]. 2017 May;84(1):226–34. Available from: https://linkinghub.elsevier.com/retrieve/pii/S0010482517300872

54. Šel Davorka, Cukjati D, Batiuskaite D, Slivnik T, Mir LM, Miklavčič D. Sequential Finite Element Model of Tissue Electropermeabilization. IEEE Trans Biomed Eng [Internet]. 2005 May;52(5):816–27. Available from: http://ieeexplore.ieee.org/document/1420703/

55. Vélez Salazar FM, Patiño Arcila ID, Ruiz Villa CA, Hernández-Blanquisett A. In-silico study about the influence of electroporation parameters on the chemotherapeutic drug transport in cancer tissues using the meshless method of approximate particular solutions. Computers & Mathematics with Applications [Internet]. 2022 Nov 1 [cited 2022 Sep 6];125:116–35. Available from: https://linkinghub.elsevier.com/retrieve/pii/S0898122122003546

56. Faurie C, Rebersek M, Golzio M, Kanduser M, Escoffre JM, Pavlin M, et al. Electro-mediated gene transfer and expression are controlled by the life-time of DNA/membrane complex formation. Journal of Gene Medicine. 2010;12(1).

57. Escoffre JM, Portet T, Favard C, Teissié J, Dean DS, Rols MP. Electromediated formation of DNA complexes with cell membranes and its consequences for gene delivery. Biochim Biophys Acta Biomembr. 2011;1808(6).

58. Kramar P, Miklavcic D, Lebar AM. A System for the Determination of Planar Lipid Bilayer Breakdown Voltage and Its Applications. IEEE Trans Nanobioscience [Internet]. 2009 Jun;8(2):132–8. Available from: http://ieeexplore.ieee.org/document/4957102/

59. Kingham TP, Karkar AM, D’Angelica MI, Allen PJ, Dematteo RP, Getrajdman GI, et al. Ablation of perivascular hepatic malignant tumors with irreversible electroporation. J Am Coll Surg. 2012;215(3).

60. Philips P, Hays D, Martin RCG. Irreversible electroporation ablation (IRE) of unresectable soft tissue tumors: Learning curve evaluation in the first 150 patients treated. PLoS One. 2013;8(11).

61. Boyd B, Becker S. Modeling of In Vivo Tissue Electroporation and Cellular Uptake Enhancement. IFAC-PapersOnLine [Internet]. 2015 Sep 1;48(20):255–60. Available from: https://linkinghub.elsevier.com/retrieve/pii/S240589631502039X

62. Shirakashi R, Sukhorukov VL, Tanasawa I, Zimmermann U. Measurement of the permeability and resealing time constant of the electroporated mammalian cell membranes. Int J Heat Mass Transf [Internet]. 2004 Oct;47(21):4517–24. Available from: https://linkinghub.elsevier.com/retrieve/pii/S0017931004001401

63. Pavšelj N, Préat V, Miklavčič D. A Numerical Model of Skin Electropermeabilization Based on In Vivo Experiments. Ann Biomed Eng [Internet]. 2007 Nov 14;35(12):2138–44. Available from: http://link.springer.com/10.1007/s10439-007-9378-7

64. Vélez Salazar FM, Patiño Arcila ID, Ruiz Villa CA. Simulation of the influence of voltage level and pulse spacing on the efficiency, aggressiveness and uniformity of the electroporation process in tissues using meshless techniques. Int J Numer Method Biomed Eng [Internet]. 2020 Mar 21;36(3):e3304. Available from: https://onlinelibrary.wiley.com/doi/abs/10.1002/cnm.3304

65. Puc M, Kotnik T, Mir LM, Miklavčič D. Quantitative model of small molecules uptake after in vitro cell electropermeabilization. Bioelectrochemistry [Internet]. 2003 Aug;60(1–2):1–10. Available from: https://linkinghub.elsevier.com/retrieve/pii/S1567539403000215

1. ***Additional Figures***

**S1 Fig. Spatio-temporal evolution of the presence and interaction of reaction and transport mechanisms -** $\boldsymbol{E=0}\boldsymbol{kV/m}$**,** $\boldsymbol{\lambda}_{\boldsymbol{inl}}\boldsymbol{=0.01}\boldsymbol{m/s}$ **and** $\boldsymbol{TPK}$**.**

**S2 Fig. Spatio-temporal evolution of** ${\boldsymbol{C}_{\boldsymbol{2}}}/{\boldsymbol{C}_{\boldsymbol{1}}}$ **ratios that account for internalization and externalization rates -** $\boldsymbol{E=0}\boldsymbol{kV/m}$**,** $\boldsymbol{\lambda}_{\boldsymbol{inl}}\boldsymbol{=0.001}\boldsymbol{m/s}$ **and** $\boldsymbol{TPK}$**.**

**S3 Fig. Spatio-temporal evolution of** ${\boldsymbol{C}_{\boldsymbol{2}}}/{\boldsymbol{C}_{\boldsymbol{1}}}$ **ratios that account for internalization and externalization rates -** $\boldsymbol{E=0}\boldsymbol{kV/m}$**,** $\boldsymbol{\lambda}_{\boldsymbol{inl}}\boldsymbol{=0.01}\boldsymbol{m/s}$ **and** $\boldsymbol{TPK}$**.**

**S4 Fig. Spatio-temporal evolution of bound intracellular concentration** $\boldsymbol{C}_{\mathbf{3}}$ **that account for association and dissociation -** $\boldsymbol{E=0}\boldsymbol{kV/m}$**,** $\boldsymbol{\lambda}_{\boldsymbol{inl}}\boldsymbol{=0.001}\boldsymbol{m/s}$ **and** $\boldsymbol{TPK}$**.**

**S5 Fig. Spatio-temporal evolution of bound intracellular concentration** $\boldsymbol{C}_{\mathbf{3}}$ **that account for association and dissociation -** $\boldsymbol{E=0}\boldsymbol{kV/m}$**,** $\boldsymbol{\lambda}_{\boldsymbol{inl}}\boldsymbol{=0.01}\boldsymbol{m/s}$ **and** $\boldsymbol{TPK}$**.**

**S6-Fig. Spatio-temporal evolution of the presence and interaction of reaction and transport mechanisms –** $\boldsymbol{E=0}\boldsymbol{kV/m}$**,** $\boldsymbol{\lambda}_{\boldsymbol{inl}}\boldsymbol{=0.001}\boldsymbol{m/s}$ **and** $\boldsymbol{MPK}$**.**

**S7 Fig. Spatio-temporal evolution of the presence and interaction of reaction and transport mechanisms –** $\boldsymbol{E=0}\boldsymbol{kV/m}$**,** $\boldsymbol{\lambda}_{\boldsymbol{inl}}\boldsymbol{=0.01}\boldsymbol{m/s}$ **and** $\boldsymbol{MPK}$**.**

**S8 Fig. Spatio-temporal evolution of** ${\boldsymbol{C}_{\boldsymbol{2}}}/{\boldsymbol{C}_{\boldsymbol{1}}}$ **ratios that account for internalization and externalization rates -** $\boldsymbol{E=0}\boldsymbol{kV/m}$**,** $\boldsymbol{\lambda}_{\boldsymbol{inl}}\boldsymbol{=0.001}\boldsymbol{m/s}$ **and** $\boldsymbol{MPK}$**.**

**S9 Fig. Spatio-temporal evolution of** ${\boldsymbol{C}_{\boldsymbol{2}}}/{\boldsymbol{C}_{\boldsymbol{1}}}$ **ratios that account for internalization and externalization rates -** $\boldsymbol{E=0}\boldsymbol{kV/m}$**,** $\boldsymbol{\lambda}_{\boldsymbol{inl}}\boldsymbol{=0.01}\boldsymbol{m/s}$ **and** $\boldsymbol{MPK}$**.**

**S10 Fig. Spatio-temporal evolution of bound intracellular concentration** $\boldsymbol{C}_{\mathbf{3}}$ **that account for association and dissociation -** $\boldsymbol{E=0}\boldsymbol{kV/m}$**,** $\boldsymbol{\lambda}_{\boldsymbol{inl}}\boldsymbol{=0.001}\boldsymbol{m/s}$ **and** $\boldsymbol{MPK}$**.**

**S11 Fig. Spatio-temporal evolution of bound intracellular concentration** $\boldsymbol{C}_{\mathbf{3}}$ **that account for association and dissociation -** $\boldsymbol{E=0}\boldsymbol{kV/m}$**,** $\boldsymbol{\lambda}_{\boldsymbol{inl}}\boldsymbol{=0.01}\boldsymbol{m/s}$ **and** $\boldsymbol{MPK}$**.**

**S12 Fig. Spatio-temporal evolution of the presence and interaction of reaction and transport mechanisms -** $\boldsymbol{E=46}\boldsymbol{kV/m}$**,** $\boldsymbol{\lambda}_{\boldsymbol{inl}}\boldsymbol{=0.0001}\boldsymbol{m/s}$ **and** $\boldsymbol{TPK}$**.**

**S13 Fig. Spatio-temporal evolution of the presence and interaction of reaction and transport mechanisms -** $\boldsymbol{E=46}\boldsymbol{kV/m}$**,** $\boldsymbol{\lambda}_{\boldsymbol{inl}}\boldsymbol{=0.001}\boldsymbol{m/s}$ **and** $\boldsymbol{TPK}$**.**

**S14 Fig. Spatio-temporal evolution of the presence and interaction of reaction and transport mechanisms -** $\boldsymbol{E=46}\boldsymbol{kV/m}$**,** $\boldsymbol{\lambda}_{\boldsymbol{inl}}\boldsymbol{=0.01}\boldsymbol{m/s}$ **and** $\boldsymbol{TPK}$**.**

**S15 Fig. Spatio-temporal evolution of** ${\boldsymbol{C}_{\boldsymbol{2}}}/{\boldsymbol{C}_{\boldsymbol{1}}}$ **ratios that account for internalization and externalization rates -** $\boldsymbol{E=46}\boldsymbol{kV/m}$**,** $\boldsymbol{\lambda}_{\boldsymbol{inl}}\boldsymbol{=0.001}\boldsymbol{m/s}$ **and** $\boldsymbol{TPK}$**.**

**S16 Fig. Spatio-temporal evolution of** ${\boldsymbol{C}_{\boldsymbol{2}}}/{\boldsymbol{C}_{\boldsymbol{1}}}$ **ratios that account for internalization and externalization rates -** $\boldsymbol{E=46}\boldsymbol{kV/m}$**,** $\boldsymbol{\lambda}_{\boldsymbol{inl}}\boldsymbol{=0.01}\boldsymbol{m/s}$ **and** $\boldsymbol{TPK}$**.**

**S17 Fig. Spatio-temporal evolution of bound intracellular concentration** $\boldsymbol{C}_{\mathbf{3}}$ **that account for association and dissociation -** $\boldsymbol{E=46}\boldsymbol{kV/m}$**,** $\boldsymbol{\lambda}_{\boldsymbol{inl}}\boldsymbol{=0.001}\boldsymbol{m/s}$ **and** $\boldsymbol{TPK}$**.**

**Fig 18.** **Spatio-temporal evolution of bound intracellular concentration** $\boldsymbol{C}_{\mathbf{3}}$ **that account for association and dissociation -** $\boldsymbol{E=46}\boldsymbol{kV/m}$**,** $\boldsymbol{\lambda}_{\boldsymbol{inl}}\boldsymbol{=0.01}\boldsymbol{m/s}$ **and** $\boldsymbol{TPK}$**.**

**S19 Fig. Spatio-temporal evolution of the presence and interaction of reaction and transport mechanisms -** $\boldsymbol{E=46}\boldsymbol{kV/m}$**,** $\boldsymbol{\lambda}_{\boldsymbol{inl}}\boldsymbol{=0.0001}\boldsymbol{m/s}$ **and** $\boldsymbol{MPK}$**.**

**S20 Fig. Spatio-temporal evolution of the presence and interaction of reaction and transport mechanisms -** $\boldsymbol{E=46}\boldsymbol{kV/m}$**,** $\boldsymbol{\lambda}_{\boldsymbol{inl}}\boldsymbol{=0.001}\boldsymbol{m/s}$ **and** $\boldsymbol{MPK}$**.**

**S21 Fig. Spatio-temporal evolution of the presence and interaction of reaction and transport mechanisms -** $\boldsymbol{E=46}\boldsymbol{kV/m}$**,** $\boldsymbol{\lambda}_{\boldsymbol{inl}}\boldsymbol{=0.01}\boldsymbol{m/s}$ **and** $\boldsymbol{MPK}$**.**

**S22 Fig. Spatio-temporal evolution of** ${\boldsymbol{C}_{\boldsymbol{2}}}/{\boldsymbol{C}_{\boldsymbol{1}}}$ **ratios that account for internalization and externalization rates -** $\boldsymbol{E=46}\boldsymbol{kV/m}$**,** $\boldsymbol{\lambda}_{\boldsymbol{inl}}\boldsymbol{=0.001}\boldsymbol{m/s}$ **and** $\boldsymbol{MPK.}$

**S23 Fig.** **Spatio-temporal evolution of** ${\boldsymbol{C}_{\boldsymbol{2}}}/{\boldsymbol{C}_{\boldsymbol{1}}}$ **ratios that account for internalization and externalization rates -** $\boldsymbol{E=46}\boldsymbol{kV/m}$**,** $\boldsymbol{\lambda}_{\boldsymbol{inl}}\boldsymbol{=0.01}\boldsymbol{m/s}$ **and** $\boldsymbol{MPK.}$

**S24 Fig. Spatio-temporal evolution of bound intracellular concentration** $\boldsymbol{C}_{\mathbf{3}}$ **that account for association and dissociation -** $\boldsymbol{E=46}\boldsymbol{kV/m}$**,** $\boldsymbol{\lambda}_{\boldsymbol{inl}}\boldsymbol{=0.001}\boldsymbol{m/s}$ **and** $\boldsymbol{MPK.}$

**S25 Fig. Spatio-temporal evolution of bound intracellular concentration** $\boldsymbol{C}_{\mathbf{3}}$ **that account for association and dissociation -** $\boldsymbol{E=46}\boldsymbol{kV/m}$**,** $\boldsymbol{\lambda}_{\boldsymbol{inl}}\boldsymbol{=0.01}\boldsymbol{m/s}$ **and** $\boldsymbol{MPK.}$

**S26 Fig. Spatio-temporal evolution of the presence and interaction of reaction and transport mechanisms -** $\boldsymbol{E=70}\boldsymbol{kV/m}$**,** $\boldsymbol{\lambda}_{\boldsymbol{inl}}\boldsymbol{=0.0001}\boldsymbol{m/s}$ **and** $\boldsymbol{TPK.}$

**S27 Fig. Spatio-temporal evolution of the presence and interaction of reaction and transport mechanisms -** $\boldsymbol{E=70}\boldsymbol{kV/m}$**,** $\boldsymbol{\lambda}_{\boldsymbol{inl}}\boldsymbol{=0.001}\boldsymbol{m/s}$ **and** $\boldsymbol{TPK.}$

**S28 Fig. Spatio-temporal evolution of the presence and interaction of reaction and transport mechanisms -** $\boldsymbol{E=70}\boldsymbol{kV/m}$**,** $\boldsymbol{\lambda}_{\boldsymbol{inl}}\boldsymbol{=0.01}\boldsymbol{m/s}$ **and** $\boldsymbol{TPK.}$

**S29 Fig. Spatio-temporal evolution of** ${\boldsymbol{C}_{\boldsymbol{2}}}/{\boldsymbol{C}_{\boldsymbol{1}}}$ **ratios that account for internalization and externalization rates -** $\boldsymbol{E=70}\boldsymbol{kV/m}$**,** $\boldsymbol{\lambda}_{\boldsymbol{inl}}\boldsymbol{=0.0001}\boldsymbol{m/s}$ **and** $\boldsymbol{TPK.}$

**S30 Fig. Spatio-temporal evolution of** ${\boldsymbol{C}_{\boldsymbol{2}}}/{\boldsymbol{C}_{\boldsymbol{1}}}$ **ratios that account for internalization and externalization rates -** $\boldsymbol{E=70}\boldsymbol{kV/m}$**,** $\boldsymbol{\lambda}_{\boldsymbol{inl}}\boldsymbol{=0.001}\boldsymbol{m/s}$ **and** $\boldsymbol{TPK.}$

**S31 Fig. Spatio-temporal evolution of** ${\boldsymbol{C}_{\boldsymbol{2}}}/{\boldsymbol{C}_{\boldsymbol{1}}}$ **ratios that account for internalization and externalization rates -** $\boldsymbol{E=70}\boldsymbol{kV/m}$**,** $\boldsymbol{\lambda}_{\boldsymbol{inl}}\boldsymbol{=0.01}\boldsymbol{m/s}$ **and** $\boldsymbol{TPK.}$

**S32 Fig. Spatio-temporal evolution of bound intracellular concentration** $\boldsymbol{C}_{\mathbf{3}}$ **that account for association and dissociation -** $\boldsymbol{E=70}\boldsymbol{kV/m}$**,** $\boldsymbol{\lambda}_{\boldsymbol{inl}}\boldsymbol{=0.0001}\boldsymbol{m/s}$ **and** $\boldsymbol{TPK.}$

**S33 Fig. Spatio-temporal evolution of bound intracellular concentration** $\boldsymbol{C}_{\mathbf{3}}$ **that account for association and dissociation -** $\boldsymbol{E=70}\boldsymbol{kV/m}$**,** $\boldsymbol{\lambda}_{\boldsymbol{inl}}\boldsymbol{=0.001}\boldsymbol{m/s}$ **and** $\boldsymbol{TPK.}$

**S34 Fig. Spatio-temporal evolution of bound intracellular concentration** $\boldsymbol{C}_{\mathbf{3}}$ **that account for association and dissociation -** $\boldsymbol{E=70}\boldsymbol{kV/m}$**,** $\boldsymbol{\lambda}_{\boldsymbol{inl}}\boldsymbol{=0.01}\boldsymbol{m/s}$ **and** $\boldsymbol{TPK.}$

**S35 Fig. Spatio-temporal evolution of the presence and interaction of reaction and transport mechanisms -** $\boldsymbol{E=70}\boldsymbol{kV/m}$**,** $\boldsymbol{\lambda}_{\boldsymbol{inl}}\boldsymbol{=0.0001}\boldsymbol{m/s}$ **and** $\boldsymbol{MPK.}$

**S36 Fig. Spatio-temporal evolution of the presence and interaction of reaction and transport mechanisms -** $\boldsymbol{E=70}\boldsymbol{kV/m}$**,** $\boldsymbol{\lambda}_{\boldsymbol{inl}}\boldsymbol{=0.001}\boldsymbol{m/s}$ **and** $\boldsymbol{MPK.}$

**S37 Fig. Spatio-temporal evolution of the presence and interaction of reaction and transport mechanisms -** $\boldsymbol{E=70}\boldsymbol{kV/m}$**,** $\boldsymbol{\lambda}_{\boldsymbol{inl}}\boldsymbol{=0.01}\boldsymbol{m/s}$ **and** $\boldsymbol{MPK.}$

**S38 Fig.** **Spatio-temporal evolution of** ${\boldsymbol{C}_{\boldsymbol{2}}}/{\boldsymbol{C}_{\boldsymbol{1}}}$ **ratios that account for internalization and externalization rates -** $\boldsymbol{E=70}\boldsymbol{kV/m}$**,** $\boldsymbol{\lambda}_{\boldsymbol{inl}}\boldsymbol{=0.0001}\boldsymbol{m/s}$ **and** $\boldsymbol{MPK.}$

**S39 Fig. Spatio-temporal evolution of** ${\boldsymbol{C}_{\boldsymbol{2}}}/{\boldsymbol{C}_{\boldsymbol{1}}}$ **ratios that account for internalization and externalization rates -** $\boldsymbol{E=70}\boldsymbol{kV/m}$**,** $\boldsymbol{\lambda}_{\boldsymbol{inl}}\boldsymbol{=0.001}\boldsymbol{m/s}$ **and** $\boldsymbol{MPK.}$

**S40 Fig. Spatio-temporal evolution of** ${\boldsymbol{C}_{\boldsymbol{2}}}/{\boldsymbol{C}_{\boldsymbol{1}}}$ **ratios that account for internalization and externalization rates -** $\boldsymbol{E=70}\boldsymbol{kV/m}$**,** $\boldsymbol{\lambda}_{\boldsymbol{inl}}\boldsymbol{=0.01}\boldsymbol{m/s}$ **and** $\boldsymbol{MPK.}$

**S41 Fig. Spatio-temporal evolution of bound intracellular concentration** $\boldsymbol{C}_{\mathbf{3}}$ **that account for association and dissociation -** $\boldsymbol{E=70}\boldsymbol{kV/m}$**,** $\boldsymbol{\lambda}_{\boldsymbol{inl}}\boldsymbol{=0.0001}\boldsymbol{m/s}$ **and** $\boldsymbol{MPK.}$

**S42 Fig. Spatio-temporal evolution of bound intracellular concentration** $\boldsymbol{C}_{\mathbf{3}}$ **that account for association and dissociation -** $\boldsymbol{E=70}\boldsymbol{kV/m}$**,** $\boldsymbol{\lambda}_{\boldsymbol{inl}}\boldsymbol{=0.001}\boldsymbol{m/s}$ **and** $\boldsymbol{MPK.}$

**S43 Fig. Spatio-temporal evolution of bound intracellular concentration** $\boldsymbol{C}_{\mathbf{3}}$ **that account for association and dissociation -** $\boldsymbol{E=70}\boldsymbol{kV/m}$**,** $\boldsymbol{\lambda}_{\boldsymbol{inl}}\boldsymbol{=0.01}\boldsymbol{m/s}$ **and** $\boldsymbol{MPK.}$

1. ***Algorithms of the Boolean model.***

- ***Algorithm 1: Loading of concentration fields and invocation of main functions.***

clear all

close all

% Load of concentration field

% Concentration_field is a data file with information of extracellular, free intracellular and bound intracellular concentrations at several points and time instants, with the following outputs:

TIME_TOTAL_ALL: Time instants of evaluation of the concentration fields.

POINTSCOLLOCATION_TOTAL_ALL: Matrix with information of indexing, global coordinates, and normal vectors (when applied) of boundary and interior points of the tissue domain at several time instants.

C1_TOTAL_ALL: Matrix with information of extracellular concentration in the interior and boundary points of the domain.

C2_TOTAL_ALL: Matrix with information of free intracellular concentration in the interior and boundary points of the domain.

C3_TOTAL_ALL: Matrix with information of bound intracellular concentration in the interior and boundary points of the domain.

E: Electric field magnitude

dpulses: Pulse spacing

Npulses: Number of continuous pulses

Time_elec: Duration of each electroporation protocol

Nelec: Number of electroporation protocols

id_profile: Parameters of pharmacokinetic profile

lambda: Bloodstream velocity

load('Concentration_field.mat');

% FUNCTION FOR GENERATION OF CONTOURS OF CONCENTRATION RATIOS

[Time,DataX,DataY,Concentration_Ratio,t1,t2]=Data_subplots_generator_Contour_Rex_int_time_7(POINTSCOLLOCATION_TOTAL_ALL,C1_TOTAL_ALL,C2_TOTAL_ALL,C3_TOTAL_ALL,E,dpulses,Npulses,Time_elec,Nelec,id_profile,lambda);

% FUNCTION FOR GENERATION OF CONTOURS OF TRANSPORT AND REACTION MECHANISMS

[INDEX,mechanism]=contour_transport_mechanisms_7(E,dpulses, ,Npulses,Time_elec,Nelec,id_profile,lambda,Time,DataX,DataY,Concentration_Ratio,t1,t2,TIME_TOTAL_ALL,POINTSCOLLOCATION_TOTAL_ALL,C3_TOTAL_ALL,C1_TOTAL_ALL,C2_TOTAL_ALL);

- ***Algorithm 2 (Invoked by Algorithm 1): Generation of contours of concentration ratios.***

function

[Time,DataX,DataY,Concentration_Ratio,t1,t2]=Data_subplots_generator_Contour_Rex_int_time_7(POINTSCOLLOCATION_TOTAL_ALL,C1_TOTAL_ALL,C2_TOTAL_ALL,C3_TOTAL_ALL,E,dpulses,Npulses,Time_elec,Nelec,id_profile,lambda)

close all

% DISTRIBUTION OF PLOT

number_rows=3;

number_columns=4;

% Variables for plot identification

Letters=["(a) Time=","(b) Time=","(c) Time=","(d) Time=","(e) Time=","(f) Time=","(g) Time=","(h) Time=","(i) Time=","(j) Time=","(k) Time=","(l) Time=","(m) Time=","(n) Time=","(o) Time=","(p) Time=","(q) Time=","(r) Time="];

% Creation of initial contours

for i=2:3

[COORDR,COORDZ]=meshgrid(16:1:200,0:1:400);

Rext_int_contour=zeros(size(COORDR));

figure

contourf(COORDR,COORDZ,Rext_int_contour,[0,0.0001],'LineStyle','none');

set(gca,'FontSize',14)

axis equal

colormap parula

cp=colorbar;

clim([0,1e-3]);

cp.Label.String = ['$C_',num2str(i),'/C_1$'];%'$C_2/C_1$';

cp.Label.Interpreter = 'latex';

cp.Label.FontSize = 18;

title({'Time=0h'},'FontSize',18,'interpreter','latex')

xlabel('$r \ (\mu m)$','FontSize',20,'interpreter','latex')

ylabel('$z \ (\mu m)$','FontSize',20,'interpreter','latex')

name_ref=['Fig_ref_',num2str(i),'.fig'];

savefig(name_ref);

close

end

% Creation of initial contours for bound intracellular concentration

[COORDR,COORDZ]=meshgrid(16:(200-16)/100:200,0:(400-0)/100:400);

C3_contour=zeros(size(COORDR));

figure

contourf(COORDR,COORDZ,C3_contour,[0,0.0001],'LineStyle','none');

set(gca,'FontSize',14)

axis equal

colormap parula

cp=colorbar

clim([0,1e-3]);

cp.Label.String = ['$C_3 (\mu M)$'];%'$C_2/C_1$';

cp.Label.Interpreter = 'latex'

cp.Label.FontSize = 18;

title({['Time=0h']},'FontSize',18,'interpreter','latex')

xlabel('$r \ (\mu m)$','FontSize',20,'interpreter','latex')

ylabel('$z \ (\mu m)$','FontSize',20,'interpreter','latex')

name_ref=['Fig_ref_',num2str(4),'.fig'];

savefig(name_ref);

close

%--------------------------------------------------------------------------

% Generation of data and contour plots

for l=2:4 % Type of contour

for i=1:length(E) % Cycle for Electric field magnitude

for j=1:length(lambda) % Cycle for the blood velocity (lambda)

for id=1:length(id_profile) % Cycle for the pharmakocinetic

profile (id_profile)

s=1;

fnew=figure;

for k=1:length(Time) % Cycle for the time

% Generation of contour plot

if s==1

name_ref=['Fig_ref_',num2str(l),'.fig'];

fig1=openfig(name_ref);

else

if or(l==2,l==3)

Name_identifier=['Contour C',num2str(l),'overC1_',num2str(Time(k)),'_',num2str(E(i)),'_10_0.16667_6_5_24_',num2str(lambda(j)),'_',num2str(id_profile(id))];

fig1 = openfig([Name_identifier,'.fig'],'reuse');

else

% Obtention of collocation points and concentration POINTSCOLLOCATION(1:length(POINTSCOLLOCATION_TOTAL_ALL),2:3)=POINTSCOLLOCATION_TOTAL_ALL(i,1,1,1,1,j,id,Time(k)/0.5,1:length(POINTSCOLLOCATION_TOTAL_ALL),1:2);

C3(1:length(C3_TOTAL_ALL))=C3_TOTAL_ALL(i,1,1,1,1,j,id,1:length(C3_TOTAL_ALL),Time(k)/0.5);

ymin=min(find(C3==0));

if isempty(ymin)==0

POINTSCOLLOCATION=POINTSCOLLOCATION(1:ymin-1,1:3);

C3=C3(1:ymin-1);

end

% Calculation of concentration field.

limitinfr=min(POINTSCOLLOCATION(:,2));

[COORDR,COORDZ]=meshgrid(limitinfr:(200-limitinfr)/15:200,0:(400-0)/15:400);

[C3_field]=gen_contours(COORDR,COORDZ,POINTSCOLLOCATION,C3,1);

[C3_field]=correction_contour_plots(COORDR,COORDZ,C3_field);

% Graphic of concentration

figure

contourf(COORDR,COORDZ,C3_field,'LineStyle','none');

fig1=gcf;

set(gca,'FontSize',14)

axis equal

colormap parula

cp=colorbar

cp.Label.String = '$C_3 (\mu M)$';

cp.Label.Interpreter = 'latex'

cp.Label.FontSize = 18;

title({['Time=',num2str(Time(k)),'h']},'FontSize',18,'interpreter','latex')

xlabel('$r \ (\mu m)$','FontSize',20,'interpreter','latex')

ylabel('$z \ (\mu m)$','FontSize',20,'interpreter','latex')

clear C3 POINTSCOLLOCATION

end

end

pause(5);

h=gca;

h1=findobj(gca);

% Extraction of data of contours

[t1(i,j,k),t2(i,j,k)]=size(h1(2).XData);

DataX(i,j,k,1:t1(i,j,k),1:t2(i,j,k))=h1(2).XData;

DataY(i,j,k,1:t1(i,j,k),1:t2(i,j,k))=h1(2).YData;

limit_inf_x=min(min(h1(2).XData));

Concentration_Ratio(l,i,j,id,k,1:t1(i,j,k),1:t2(i,j,k))=h1(2).ZData;

% Fitting of curves previous to subplot

% Creation of subplot

h_copy = copyobj(h,fnew);

hsub=subplot(number_rows,number_columns,s,h_copy);

close(fig1);

% Change of FontSize

hsub.FontSize=10;

hsub.Title.String=strcat(Letters(s),num2str(Time(k)),"h");

hsub.Title.Interpreter='latex';

%hsub.Title.Position(2)=-150;

hsub.Title.FontSize=10;

colorbar

% Fitting of XThick and YThick

hsub.XTick=[limit_inf_x,100,200];

xtickformat('%.0f');

%hsub.YTick=0:0.5e-8:3e-8;

h=colorbar;

t=get(h,'Limits');

set(h,'Ticks',linspace(t(1),t(2),5));

s=s+1;

end

if or(l==2,l==3)

savefig(['Contour C',num2str(l),'overC1_',num2str(E(i)),'_',num2str(lambda(j)),'_',num2str(id_profile(id)),'.fig']);

else

savefig(['Contour C3_',num2str(E(i)),'_',num2str(lambda(j)),'_',num2str(id_profile(id)),'.fig']);

end

end

end

end

end

%--------------------------------------------------------------------------

- ***Algorithms 3 and 4 (Invoked by Algorithm 2): Generation of interpolated data for contours of concentration ratios***

function [Var_field] = gen_contours(COORDR,COORDZ,POINTSCOLLOCATION,Var,negative_correction)

% This function generates data for the contour of the field variable

[t1,t2]=size(COORDR);

for i=1:t1

for j=1:t2

[y1,y2]=sort(sqrt((POINTSCOLLOCATION(:,2)-COORDR(i,j)).^2+(POINTSCOLLOCATION(:,3)-COORDZ(i,j)).^2),'ascend');

if or(or(and(i==t1,j==1),and(i==t1,j==t2)),or(and(i==1,j==1),and(i==1,j==t2)))

syms r z y

M=[r-POINTSCOLLOCATION(y2(1),2),z-POINTSCOLLOCATION(y2(1),3),y-Var(y2(1));POINTSCOLLOCATION(y2(2),2)-POINTSCOLLOCATION(y2(1),2),POINTSCOLLOCATION(y2(2),3)-POINTSCOLLOCATION(y2(1),3),Var(y2(2))-Var(y2(1));...

POINTSCOLLOCATION(y2(3),2)-POINTSCOLLOCATION(y2(1),2),POINTSCOLLOCATION(y2(3),3)-POINTSCOLLOCATION(y2(1),3),Var(y2(3))-Var(y2(1))];

eqn=subs(subs(det(M),r,COORDR(i,j)),z,COORDZ(i,j))==0;

Var_field(i,j)=double(solve(eqn));

else

Var_field(i,j)=(Var(y2(1)));

end

% Correction of small negative values

if negative_correction==1

if Var_field(i,j)<0

Var_field(i,j)=0;

end

end

end

end

%--------------------------------------------------------------------------

function [Var_field]=correction_contour_plots(COORDR,COORDZ,Var_field)

% This function corrects contour plots using linear RBF interpolation

% Size of field

[t1,t2]=size(COORDR);

% Detection of Infinite and Undetermined values in Var_field

[yinf1,yinf2]=find(isinf(Var_field)==1);

[ynan1,ynan2]=find(isnan(Var_field)==1);

y1=[yinf1,ynan1];

y2=[yinf2,ynan2];

% Cycle for calculation of correction points

for i=1:length(y1)

% Vector of problematic positions

posrow=[];

poscol=[];

% Point 1

if y2(i)>1

posrow=[posrow,y1(i)];

poscol=[poscol,y2(i)-1];

end

% Point 2

if y2(i)<t2

posrow=[posrow,y1(i)];

poscol=[poscol,y2(i)+1];

end

if y1(i)>1

% Point 3

posrow=[posrow,y1(i)-1];

poscol=[poscol,y2(i)];

if y2(i)>1

% Point 4

posrow=[posrow,y1(i)-1];

poscol=[poscol,y2(i)-1];

end

if y2(i)<t2

% Point 5

posrow=[posrow,y1(i)-1];

poscol=[poscol,y2(i)+1];

end

end

if y1(i)<t2

% Point 6

posrow=[posrow,y1(i)+1];

poscol=[poscol,y2(i)];

if y2(i)>1

% Point 7

posrow=[posrow,y1(i)+1];

poscol=[poscol,y2(i)-1];

end

if y2(i)<t2

% Point 8

posrow=[posrow,y1(i)+1];

poscol=[poscol,y2(i)+1];

end

end

% Cycle for filtering of data

for j=1:length(posrow)

if or(isnan(Var_field(posrow(j),poscol(j)))==1,isinf(Var_field(posrow(j),poscol(j)))==1)

posrow(j)=0;

poscol(j)=0;

end

end

posrow(find(posrow==0))=[];

poscol(find(poscol==0))=[];

% Cycle for calculation of RBF interpolation

for j=1:length(posrow)

for k=j+1:length(posrow)

R(j,k)=sqrt((COORDR(posrow(j),poscol(j))-COORDR(posrow(k),poscol(k))).^2+...

(COORDZ(posrow(j),poscol(j))-COORDZ(posrow(k),poscol(k))).^2);

end

V(j,1)=Var_field(posrow(j),poscol(j));

R(j+1:length(posrow),j)=R(j,j+1:length(posrow))';

A(1,j)=sqrt((COORDR(posrow(j),poscol(j))-COORDR(y1(i),y2(i))).^2+...

(COORDZ(posrow(j),poscol(j))-COORDZ(y1(i),y2(i))).^2);

end

Var_field(y1(i),y2(i))=A*(inv(R)*V);

clear R V A

end

%--------------------------------------------------------------------------

- ***Algorithms 5 (Invoked by Algorithm 1): Generation of contours of reaction and transport mechanisms at several time instants.***

function [INDEX,mechanism]=contour_transport_mechanisms_7(E,dpulses, ,Npulses,Time_elec,Nelec,id_profile,lambda,Time,DataX,DataY,Concentration_Ratio,t1,t2,TIME_TOTAL_ALL,POINTSCOLLOCATION_TOTAL_ALL,C3_TOTAL_ALL,C1_TOTAL_ALL,C2_TOTAL_ALL)

close all

% CONTOURS OF TRANSPORT MECHANISM

Letters=["(a) ","(b) ","(c) ","(d) ","(e) ","(f) ","(g) ","(h) ","(i) ","(j) ","(k) ","(l) ","(m) ","(n) ","(o) ","(p) ","(q) ","(r) "];

for i=1:length(E) % Cycle for Electric field magnitude

for j=1:length(lambda) % Cycle for the pulse spacing (dpulses)

for id=1:length(id_profile)

s=1;

fnew=figure;

incre_x=0;

incre_y=0;

for k=1:length(Time)-1 % Cycle for the time

TimeAux(1:48)=TIME_TOTAL_ALL(i,1,1,1,1,j,id,1:48)/3600;

[y1to]=find(Time(k)==TimeAux);

if isempty(y1to)==1

y1to=1;

PointsCollocationto(1:length(POINTSCOLLOCATION_TOTAL_ALL),1:2)=POINTSCOLLOCATION_TOTAL_ALL(i,1,1,1,1,j,id,y1to,1:length(POINTSCOLLOCATION_TOTAL_ALL),1:2);

Concentration1to(1:length(C1_TOTAL_ALL))=0;

Concentration3to(1:length(C3_TOTAL_ALL))=0;

else

PointsCollocationto(1:length(POINTSCOLLOCATION_TOTAL_ALL),1:2)=POINTSCOLLOCATION_TOTAL_ALL(i,1,1,1,1,j,id,y1to,1:length(POINTSCOLLOCATION_TOTAL_ALL),1:2);

Concentration1to(1:length(C1_TOTAL_ALL))=C1_TOTAL_ALL(i,1,1,1,1,j,id,1:length(C1_TOTAL_ALL),y1to);

Concentration3to(1:length(C3_TOTAL_ALL))=C3_TOTAL_ALL(i,1,1,1,1,j,id,1:length(C3_TOTAL_ALL),y1to);

end

% For time step tf

[y1tf]=find(Time(k+1)==TimeAux);

if isempty(y1tf)==1

y1tf=1; PointsCollocationtf(1:length(POINTSCOLLOCATION_TOTAL_ALL),1:2)=POINTSCOLLOCATION_TOTAL_ALL(i,1,1,1,1,j,id,y1tf,1:length(POINTSCOLLOCATION_TOTAL_ALL),1:2);

Concentration1tf(1:length(C1_TOTAL_ALL))=0;

Concentration3tf(1:length(C3_TOTAL_ALL))=0;

else

PointsCollocationtf(1:length(POINTSCOLLOCATION_TOTAL_ALL),1:2)=POINTSCOLLOCATION_TOTAL_ALL(i,1,1,1,1,j,id,y1tf,1:length(POINTSCOLLOCATION_TOTAL_ALL),1:2);

Concentration1tf(1:length(C1_TOTAL_ALL))=C1_TOTAL_ALL(i,1,1,1,1,j,id,1:length(C1_TOTAL_ALL),y1tf);

Concentration3tf(1:length(C3_TOTAL_ALL))=C3_TOTAL_ALL(i,1,1,1,1,j,id,1:length(C3_TOTAL_ALL),y1tf);

end

% Obtention of contour of concentration ratios

X_Data(1:t1(i,j,k),1:t2(i,j,k))=DataX(i,j,k,1:t1(i,j,k),1:t2(i,j,k));

Y_Data(1:t1(i,j,k),1:t2(i,j,k))=DataY(i,j,k,1:t1(i,j,k),1:t2(i,j,k));

C2_over_C1_to(1:t1(i,j,k),1:t2(i,j,k))=Concentration_Ratio(2,i,j,id,k,1:t1(i,j,k),1:t2(i,j,k));

C2_over_C1_tf(1:t1(i,j,k),1:t2(i,j,k))=Concentration_Ratio(2,i,j,id,k+1,1:t1(i,j,k),1:t2(i,j,k));

C3_over_C1_to(1:t1(i,j,k),1:t2(i,j,k))=Concentration_Ratio(3,i,j,id,k,1:t1(i,j,k),1:t2(i,j,k));

C3_over_C1_tf(1:t1(i,j,k),1:t2(i,j,k))=Concentration_Ratio(3,i,j,id,k+1,1:t1(i,j,k),1:t2(i,j,k));

% Plot of contour of mechanism

[INDEX,Index_Diff,mechanism,colorin,CHANGE_EXTRA]=generation_contour_mechanisms_7_mod(X_Data,Y_Data,C2_over_C1_to,C2_over_C1_tf,C3_over_C1_to,C3_over_C1_tf,PointsCollocationto,Concentration3to,PointsCollocationtf,Concentration3tf,Concentration1to,Concentration1tf);

% Plot of contour of mechanism

hsub(s)=subplot(3,4,s);

surf(X_Data,Y_Data,INDEX,'EdgeColor','none','FaceAlpha',0.8)

hold on

[p1,p2]=find(CHANGE_EXTRA==1);

plot(X_Data(p1,p2),Y_Data(p1,p2),'.k','MarkerSize',4);

view([0 90]);

axis equal

axis([min(min(X_Data)) max(max(X_Data)) min(min(Y_Data)) max(max(Y_Data))]);

colormap(hsub(s),colorin);

for m=1:length(Index_Diff)

annotation('textbox', [0.238+incre_x 0.863-incre_y 0.85 0.05], 'String',mechanism(m),'FitBoxToText','on','BackgroundColor',colorin(m,1:3),'FontSize',8)

end

hsub(s).XTick=[min(min(X_Data)), 100, 200]

xtickformat('%.0f');

set(gca,'FontSize',8)

title(strcat(Letters(s),num2str(Time(k)),"h to t=",num2str(Time(k+1)),"h"),'FontSize',10,'interpreter','latex')

xlabel('$r \ (\mu m)$','FontSize',10,'interpreter','latex')

ylabel('$z \ (\mu m)$','FontSize',10,'interpreter','latex')

s=s+1;

hold on

incre_x=incre_x+0.207;

if mod(k,4)==0

incre_y=incre_y+0.29;

incre_x=0;

end

clear TimeAux PointsCollocationto Concentration3to PointsCollocationtf Concentration3tf X_Data Y_Data C2_over_C1_to C2_over_C1_tf C3_over_C1_to C3_over_C1_tf

end

savefig(['Contour Transport Mechanisms',num2str(E(i)),'_',num2str(lambda(j)),'_',num2str(id_profile(id)),'.fig'

end

end

end

%------------------------------------------------------------------------

- ***Algorithms 6 (Invoked by Algorithm 5): Application of Boolean model.***

function [INDEX,Index_Diff,mechanism,colorin,CHANGE_EXTRA]=generation_contour_mechanisms_7_mod(X_Data,Y_Data,C2_over_C1_to,C2_over_C1_tf,C3_over_C1_to,C3_over_C1_tf,PointsCollocationto,Concentration3to,PointsCollocationtf,Concentration3tf,Concentration1to,Concentration1tf)

% Generation of initial logic vector and matrices

[t1,t2]=size(X_Data);

COMPARISON(1)="(IN≥AS)⋁(IN≥ECT+)";

COMPARISON(2)="EX≤ECT-";

COMPARISON(3)="∃AS ∧ ∃IN";

COMPARISON(4)="(EX≤ECT-)∧(EX≥DIS)";

COMPARISON(5)="IN≥AS";

COMPARISON(6)="IN≥ECT+";

COMPARISON(7)="(IN≥AS)↔(IN≤ECT+)";

COMPARISON(8)="IN≤ECT+";

COMPARISON(9)="(EX≤DIS)↔(EX≥ECT-)";

COMPARISON(10)="(IN≥AS)∧(IN≤ECT+)";

COMPARISON(11)="(EX≤DIS)⋁(EX≤ECT-)";

COMPARISON(12)="(IN≤AS)⋁(IN≤ECT+)";

COMPARISON(13)="(EX≥DIS)⋁(EX≥ECT-)";

COMPARISON(14)="∃AS ∧ ∄EX ∧ ∄IN";

COMPARISON(15)="∃DIS ∧ ∃IN";

COMPARISON(16)="∃DIS ∧ ∄EX ∧ ∄IN";

COMPARISON(17)="∃AS ∧ ∃EX";

% Assignment of colors for each transport and reaction mechanism

colores=[1 0 0; 0 1 0; 0 0 1; 0 1 1;1 0 1; 1 1 0;0 0.45 0.75;0.85 0.3 0.1;...

0.9 0.7 0.12; 0.50 0.20 0.55; 0.45 0.65 0.2; 0.30 0.75 0.9; 0.65 0.1 0.2;0.35 0.9 0.8; 0.70 0.25 0.1; 0.55 0.35 0.8; 0.50 0.80 0.45];

% Creation od CHANGE_EXTRA

CHANGE_EXTRA=zeros(t1,t2);

for i=1:t1

for j=1:t2

% Determination whether there is Drug Association or Dissociation between time instants

[y1to,y2to]=min(sqrt((X_Data(i,j)-PointsCollocationto(:,1)).^2+(Y_Data(i,j)-PointsCollocationto(:,2)).^2));

[y1tf,y2tf]=min(sqrt((X_Data(i,j)-PointsCollocationtf(:,1)).^2+(Y_Data(i,j)-PointsCollocationtf(:,2)).^2));

if Concentration3to(y2to)<=Concentration3tf(y2tf)

AS=1; % There is Association

else

AS=0; % There is Dissociation

end

% Determination of change of extracellular concentration

if Concentration1to(y2to)>=Concentration1tf(y2tf) % Extracellular concentration increases

CHANGE_EXTRA(i,j)=1;

end

%------------------------------------------------------------------

% Determination whether there is Internalization or Externalization

% between time instants

if and(C2_over_C1_to(i,j)<1,C2_over_C1_tf(i,j)<1)

INTER_EXTER=1; % There is internalization at both time instants

else if and(C2_over_C1_to(i,j)>1,C2_over_C1_tf(i,j)>1)

INTER_EXTER=2; % There is externalization at both time instants

else if and(C2_over_C1_to(i,j)<1,C2_over_C1_tf(i,j)>1)

INTER_EXTER=3; % Internalization at to and Externalization at tf

else if and(C2_over_C1_to(i,j)>1,C2_over_C1_tf(i,j)<1)

INTER_EXTER=4; % Externalization at to and Internalization at tf

else if and(C2_over_C1_to(i,j)==1,C2_over_C1_tf(i,j)==1)

INTER_EXTER=5; % Neither internalization, nor externalization is present at both time

end

end

end

end

end

%------------------------------------------------------------------

% Determination of the transport mechanisms

if C2_over_C1_to(i,j)<=C2_over_C1_tf(i,j)

if C3_over_C1_to(i,j)<=C3_over_C1_tf(i,j)

if (C3_over_C1_to(i,j)/C2_over_C1_to(i,j))<=(C3_over_C1_tf(i,j)/C2_over_C1_tf(i,j))

%------------------------------------------------------

if AS==1 % There is association

if INTER_EXTER==1

INDEX(i,j)=1; % Internalization at both times

else if INTER_EXTER==2

INDEX(i,j)=2; % Externalization at both times

else if INTER_EXTER==5

INDEX(i,j)=14; % Neither externalization, nor internalization

else

INDEX(i,j)=120;

end

end

end

else % There is Dissociation

if INTER_EXTER==1

INDEX(i,j)=15;

else if INTER_EXTER==2

INDEX(i,j)=4; % Externalization at both times

else if INTER_EXTER==5

INDEX(i,j)=16;

else

INDEX(i,j)=4150;

end

end

end

end

%------------------------------------------------------

else

if AS==1 % There is Association

if INTER_EXTER==1

INDEX(i,j)=5; % There is internalization

else if INTER_EXTER==2

INDEX(i,j)=17;

else if INTER_EXTER==5

INDEX(i,j)=14;

else

INDEX(i,j)=5170;

end

end

end

else % There is Dissociation

if INTER_EXTER==1

INDEX(i,j)=6; % Internalization at both times

else if INTER_EXTER==2

INDEX(i,j)=2; % Externalization at both times

else if INTER_EXTER==5

INDEX(i,j)=16; % Neither internalization, nor externalization

else

INDEX(i,j)=620; % Internalization+Externalization

end

end

end

end

%------------------------------------------------------

end

else

if (C3_over_C1_to(i,j)/C2_over_C1_to(i,j))<=(C3_over_C1_tf(i,j)/C2_over_C1_tf(i,j))

%------------------------------------------------------

if AS==1 % There is Association

if INTER_EXTER==1

INDEX(i,j)=7; % Internalization at both time instants

else if INTER_EXTER==2

INDEX(i,j)=2; % Externalization at both time instants

else if INTER_EXTER==5

INDEX(i,j)=14; % Neither externalization, nor internalization

else

INDEX(i,j)=720; % Internalization+Externalization

end

end

end

else % There is Dissociation

if INTER_EXTER==1

INDEX(i,j)=8; % Internalization at both time instants

else if INTER_EXTER==2

INDEX(i,j)=9; % Externalization at both time instants

else if INTER_EXTER==5

INDEX(i,j)=16;

else

INDEX(i,j)=890; % Internalization + Externalization

end

end

end

end

%------------------------------------------------------

else

if AS==1 % There is Association

if INTER_EXTER==1

INDEX(i,j)=10; % Internalization at both time instants

else if INTER_EXTER==2

INDEX(i,j)=17;

else if INTER_EXTER==5

INDEX(i,j)=14;

else

INDEX(i,j)=10170;

end

end

end

else % There is Dissociation

if INTER_EXTER==1

INDEX(i,j)=15; % Internalization at both time instants

else if INTER_EXTER==2

INDEX(i,j)=11; % Externalization at both time instants

else if INTER_EXTER==5

INDEX(i,j)=16; % Neither internalization, nor externalization

else

INDEX(i,j)=15110; % Internalization+Externalization

end

end

end

end

%------------------------------------------------------

end

end

else

if C3_over_C1_to(i,j)<=C3_over_C1_tf(i,j)

if (C3_over_C1_to(i,j)/C2_over_C1_to(i,j))<=(C3_over_C1_tf(i,j)/C2_over_C1_tf(i,j))

if AS==1 % There is Association

if INTER_EXTER==1

INDEX(i,j)=12; % Internalization at both time instants

else if INTER_EXTER==2

INDEX(i,j)=17; % Externalization at both time instants

else if INTER_EXTER==5 % Neither externalization, nor internalization

INDEX(i,j)=14;

else

INDEX(i,j)=12170; % Internalization + Externalization

end

end

end

else % There is Dissociation

if INTER_EXTER==1

INDEX(i,j)=15;

else if INTER_EXTER==2

INDEX(i,j)=4; % Externalization at both time instants

else if INTER_EXTER==5

INDEX(i,j)=16;

else

INDEX(i,j)=4150;

end

end

end

end

else

if AS==1 % There is Association

if INTER_EXTER==1

INDEX(i,j)=7; % Internalization at both time instants

else if INTER_EXTER==2

INDEX(i,j)=2; % Externalization at both time instants

else if INTER_EXTER==5

INDEX(i,j)=14; % Neither internalization, nor externalization

else

INDEX(i,j)=720; % Internalization + Externalization

end

end

end

else % There is Dissociation

if INTER_EXTER==1

INDEX(i,j)=8; % Internalization at both time instants

else if INTER_EXTER==2

INDEX(i,j)=9; % Externalization at both time instants

else if INTER_EXTER==5

INDEX(i,j)=16;

else

INDEX(i,j)=890; % Internalization + Externalization

end

end

end

end

%------------------------------------------------------

end

else

if (C3_over_C1_to(i,j)/C2_over_C1_to(i,j))<=(C3_over_C1_tf(i,j)/C2_over_C1_tf(i,j))

if AS==1 % There is Association

if INTER_EXTER==1

INDEX(i,j)=12; % Internalization at both time instants

else if INTER_EXTER==2

INDEX(i,j)=17; % Externalization at both time instants

else if INTER_EXTER==5

INDEX(i,j)=14; % Neither internalization, nor externalization

else

INDEX(i,j)=12170; % Internalization + Externlization

end

end

end

else % There is dissociation

if INTER_EXTER==1

INDEX(i,j)=8; % Internalization at both time instants

else if INTER_EXTER==2

INDEX(i,j)=13; % Externalization at both time instants

else if INTER_EXTER==5

INDEX(i,j)=16;

else

INDEX(i,j)=8130; % Internalization + Externalization

end

end

end

end

%------------------------------------------------------

else

if AS==1 % There is Association

if INTER_EXTER==1

INDEX(i,j)=10; % Internalization at both time instants

else if INTER_EXTER==2

INDEX(i,j)=17;

else if INTER_EXTER==5

INDEX(i,j)=14;

else

INDEX(i,j)=10170;

end

end

end

else % There is Dissociation

if INTER_EXTER==1

INDEX(i,j)=8; % Internalization at both time instants

else if INTER_EXTER==2

INDEX(i,j)=13; % Externalization at both time instants

else if INTER_EXTER==5

INDEX(i,j)=16;

else

INDEX(i,j)=8130; % Internalization + Externalization

end

end

end

end

%-----------------------------------------------------

end

end

end

end

end

%-------------------------------------------------------------------------

% CORRECTION OF MATRIX OF INDEX

[y1,y2]=find(INDEX==0);

if isempty(y1)==0

stop=1;

end

% GENERATION OF CONTOUR PLOT

% Obtention of different indexes in the matrix

Index_Diff=unique(INDEX);

colorin=[];

mechanism=[];

for i=1:length(Index_Diff)

if Index_Diff(i)<100 % The index does not end at zero

colorin=[colorin;colores(Index_Diff(i),1:3)];

mechanism=[mechanism;COMPARISON(Index_Diff(i))];

else

a=num2str(Index_Diff(i)/10);

if length(a)==2

colorin=[colorin;(colores(str2num(a(1)),1:3)+colores(str2num(a(2)),1:3))/2];

mechanism=[mechanism;strcat(COMPARISON(str2num(a(1))),"-to-",COMPARISON(str2num(a(2))))];

else if length(a)==3

colorin=[colorin;(colores(str2num(a(1)),1:3)+colores(str2num(a(2:3)),1:3))/2];

mechanism=[mechanism;strcat(COMPARISON(str2num(a(1))),"-to-",COMPARISON(str2num(a(2:3))))];

else

colorin=[colorin;(colores(str2num(a(1:2)),1:3)+colores(str2num(a(3:4)),1:3))/2];

mechanism=[mechanism;strcat(COMPARISON(str2num(a(1:2))),"-to-",COMPARISON(str2num(a(3:4))))];

end

end

end

end

%--------------------------------------------------------------------------
