# Supplementary material for: In-silico tool based on Boolean networks and meshless simulations for prediction of reaction and transport mechanisms in the systemic administration of chemotherapeutic drugs
Source: PLoS One. 2025 Feb 7;20(2):e0315194. doi: 10.1371/journal.pone.0315194 (PMC11805580; doi:10.1371/journal.pone.0315194)
Supplement: Attachment — Submitted filename: Response letter PLOS ONE_19_09_2024.docx [file pone.0315194.s045.docx]

Dear

Baeckkyoung Sung, Ph.D.

Academic Editor

PLOS ONE

Ref: Response letter.

In this letter, the responses to Reviewer’s comments about the manuscript “*In-silico tool based on Boolean Networks and meshless simulations for prediction of reaction and transport mechanisms in the systemic administration of chemotherapeutic drugs”* submitted to the journal *“PLOS ONE”* are presented in detail, indicating the parts of the new manuscript where the corresponding corrections have been attended. We really acknowledge the thoughtful and comprehensive revision of the present manuscript since this allows improving its quality and clarity.

Sincerely,

Fabián Mauricio Vélez Salazar.

*Corresponding author.*

***REVIEWER 1.***

- **General comment.**

The authors present a work that models three variables (bloodstream velocity, pharmacokinetic dynamics and electroporation intensity) that affect the drug delivery to a human tissue. This work continues a previous one in which they build a fairly similar model. The manuscript is hard to read, unclear in its motivations, unclear in its most relevant result, unclear in its usefulness as a clinical tool and lacks proper methods, discussion and connections to state of the art results. The text and the figures can be much improved to increase the readability and relevance of the work. I cannot accept this work in its present form.

- **Comment 1.**

It is not clear if this work is exploratory or if it aims at presenting clinical interventions. The authors simulate different values of three variables: bloodstream velocity, pharmacokinetic dynamics and electroporation intensity. Only the electroporation is a variable that could be used in a clinical intervention. The blood stream is hardly useful for clinicians and the pharmacokinetic is a property of the drugs.

**Response:**

This is essentially an exploratory work, but all parameters were obtained from existing literature. Accordingly, the three levels of the electric field magnitude, $E=[0 kV/m, 46 kV/m, 70 kV/m]$, correspond to a non-electroporated tissue, reversible and irreversible thresholds of a liver tissue [1], [2]. The parameters of both pharmacokinetic profiles ($MPK$ and $TPK$) for doxorubicin were obtained from Groh et al. [3], whereas parameters of the bloodstream velocity ($\lambda_{inl}$) from Hubbard et al [4]. Additional to the electric field magnitude ($E$), the other two parameters considered here ($\lambda_{inl}$ and $PK$) have clinical relevance. Some lines were added in the new version of the manuscript about the importance of these two parameters (***See Introduction section of new manuscript, Page 4***).

The pharmacokinetic profiles stand for the time evolution of drug concentration ($C_{v}$) along the bloodstream considering processes of absorption, distribution, metabolism and elimination with little physiological details [5]. These profiles depend on the drug type, loading and maintenance doses, infusion time, perfusion rate, tissue affinity, drug lipophilicity or hydrophilicity, plasma protein binding, among other factors [5], [6]. Both mono-exponential ($MPK$) and tri-exponential profiles ($TPK$) has been considered for intravenous (IV) injection of doxorubicin in previous works [3], [4], [7], [8], [9], [10], [11], [12]. The $MPK$ is a one-compartment, bolus IV injection profile, where total drug dose is rapidly injected (order of seconds) and the time decay rate of $C_{v}$ is constant since the drug distribution and elimination rates are assumed to be uniform for all tissue groups [5], [6]. On the other hand, the $TPK$ is a multi-compartment, one-short IV injection profile, where the total drug injection does not occur immediately, but in short period of time (order of minutes), to then decrease tri-exponentially due to the consideration of different distribution and elimination rates according to the tissue group [5], [6]. In general, a mono-exponential decay is typically evidenced when the drug is prone to be distributed to only richly perfused tissues and organs, such as liver, kidney and brain [13]. The parameters of both pharmacokinetic profiles ($MPK$ and $TPK$) for doxorubicin were obtained from Groh et al. [3], where the total drug exposure, defined as the Area Under the Curve (AUC) of $C_{v} vs t$, namely, $AUC=\int_{0}^{\infty} C_{v}.dt$, which is a more useful target parameter at clinical practice, is the same for both profiles ($AUC\approx2.78 \mu Mh)$. This information about the pharmacokinetic profiles has been included in the Introduction section of new version of the manuscript ***(Page 4)***, as well as the time behavior of these profiles until $t=1h$ for comparison purposes (***Section 4, Page 19 and 20 of new version of the manuscript***).

The bloodstream velocity ($\lambda_{inl}$), which is the other parameter considered here, is directly related with the blood perfusion rate, which in turns plays a major role for the systemic administration of drugs. At clinical level, the low blood perfusion in some tumors due to the vessels compression and leakiness can lead to hypoperfusion and hypoxia. While hypoperfusion reduces the medication delivery, hypoxia can foster the tumor growth through immunosuppression, increase of metastatic potential and drug resistance, and generation of disordered angiogenesis [14]. Therefore, several therapeutic strategies have been developed to improve the blood perfusion and therapy outcomes, such as [14], [15]: vascular normalization with anti-angiogenic agents, stroma normalization with mechano-therapeutics, tumor microenvironment ($TME$) normalization with metronomic chemotherapy or nanomedicine, ultrasound sono-permeabilization, among others. These strategies are focused on the regularization of the bloodstream velocity in the vessels by reducing the endothelial gaps associated to the leakage flow rate, and/or alleviating the mechanical stresses exerted by the tumor microenvironment ($TME$) surrounding these vessels. Accordingly, the influence of the bloodstream velocity ($\lambda_{inl}$) on the reaction and transport mechanisms ($RTMs$) of chemotherapeutic drugs can have clinical importance. This information about the blood perfusion rate has been included in the Introduction section of new version of the manuscript ***(Page 4)***.

- **Comment 2.**

The authors seem to completely ignore Fick’s second law in their analysis. This causes sentences like “It is also noticed that the highest internalization rates () occur at the first hour of evaluation ( = 1ℎ), where the 2⁄1 values are the lowest in the whole time window.” That should be self-evident to anyone who knows about Fick’s second law.

**Response.**

The authors are not unaware of Fick's laws and understand that the tumor cord model defined by equations (1) and (3) considers a transmembrane transport dominated by these laws. In fact, based on the Method of Volume Averaging [16], the Authors demonstrated in previous research (Appendix of [17]) that the assumption of Fickian transport between the extracellular and intracellular spaces is valid as long as the adsorption term is linear and an effective cell membrane permeability independent of drug concentration and subsuming the effects of the predominant transmembrane transport mechanisms, are considered in the species conservation equations. Bearing in mind a fickian-dominated transmembrane transport, the Authors considered the $C_{2}/C_{1}$ ratio as the parameter to determine the presence of cellular internalization or externalization at each point in the tissue domain. As the Reviewer argues, the sentence *“It is also noticed that the highest internalization rates () occur at the first hour of evaluation* ($t=1h$)*, where the* $C_{2}/C_{1}$ *values are the lowest in the whole time window”* may sound redundant to someone who knows about Fick's laws, and this redundancy has been removed in the new version of the manuscript (***Page 22 of new version of manuscript***).

- **Comment 3.**

Two pharmacokinetic models are presented without any motivation and without presenting real cases of real drugs that follow any of these dynamics.

**Response:**

Additional information about $TPK$ and $MPK$ profiles used in the present work has been reported in ***Pages 4, 19 and 20*** of new version of the manuscript.

- **Comment 4.**

What is the motivation of the many Boolean rules? Their only use seems to be to analyse the numerous results of the simulation in colours as in a lookup table, but explaining their details takes many pages and non-useful charts and tables.

**Response.**

This work includes two kinds of results. Firstly, the extracellular ($C_{1}$), free intracellular ($C_{2}$) and bound intracellular concentrations ($C_{3}$), as well as the respective ratios ($C_{2}/C_{1}$, $C_{3}/C_{1}$ and $C_{3}/C_{2}$) are obtained from the solution of the species conservation equations in the electroporated and vasoconstricted tissues through an in-house $GMAPS$ code previously developed, calibrated and validated. The time evolution of these ratios determines the presence and interaction of the different reaction and transport mechanisms ($RTMs$) of the chemotherapeutic drug, for which a Boolean model is developed based on logical propositions, implications, and equivalent statements obtained from boolean algebra, as described in detail in *Section 3* of the manuscript. From this Boolean model, qualitative results are obtained that show the presence and interaction of the $RTMs$ at different tissue points and time instants during the chemo-therapeutic treatment. Therefore, the detailed description of the development of this Boolean model is essential to demonstrate where these results are obtained from and to ensure the reproducibility of them.

- **Comment 5.**

In the Discussion section, page 41, the authors claim that the Boolean rules “could be useful for establishing appropriate drug dosages and pharmacokinetic profiles”.
Where are the drug dosages results?
How do the authors propose that clinicians establish appropriate pharmacokinetic profiles?

**Response:**

The parameters of the two pharmacokinetic profiles (total dose, injection time, exponential decay rates, etc.) used in the present work ($TPK$ and $MPK$) were obtained from previous studies [3], [4]. These parameters allow generating a total drug exposure or Area Under Curve, defined by $AUC=\int_{0}^{\infty} C_{v}.dt$, equal to 2.78 μMh for both profiles. That is, the total drug exposure or Area Under the Curve (AUC) is a fixed value in the present work for both profiles ($MPK$ and $TPK$). However, some parameters of the $PK$ profiles can be fitted to reach a target $AUC$ or target exposures to extracellular drug $(C_{1exp}=\int_{0}^{T^{'}} C_{1}.dt)$, free intracellular drug $(C_{2exp}=\int_{0}^{T^{'}} C_{2}.dt)$ and/or bound intracellular drug $(C_{3exp}=\int_{0}^{T^{'}} C_{3}.dt)$ after a given time ($T^{'}$). For instance, by modifying the total drug dose ($D_{0}$) and time of infusion (τ’) in the equation (35), which predicts the time evolution of $C_{v}$ for $TPK$, different target values of $AUC$, $C_{1exp}$, $C_{2exp}$ and $C_{3exp}$, which are more common parameters in the clinical practice related with the cell-kill capacity of the therapy, can be obtained using the present model. Despite the $PK$ parameters of each profile are not modified here, it is worth mentioning that the present model could be used in future works for evaluating the effects of modifying these parameters on the drug concentration fields, drug exposures (which are more useful at clinical level), and the presence and interaction of the reaction and transport mechanisms ($RTMs$) of chemotherapeutic drugs. This information has been added in the ***Section 6 of new version of the manuscript (Page 39)***.

It is worth-mentioning that the present model is not intended to replace the Heuristic methods commonly used for dosing of anticancer drugs (weight-based dosing, body surface area dosing, fixed-dose prescribing, AUC-based dosing, among others), but to complement these methods. Heuristic methods have been defined based on clinical practice considering several aspects such as: type of drug, type and subtype of cancer, stage of cancer, previous health treatments, current patient’s medications and mainly, the patient’s characteristics (age, weight, height, health problems, among others). One of the main drawbacks of these methods is their poor relationship with the pharmacokinetic parameters of chemotherapeutic drugs (loading and maintenance dose, injection time, decay rates, among others), affecting the accuracy in the prediction of the drug exposure and drug clearance, and consequently, the individualization capacity of the therapy. Moreover, since some heuristic models are not able to correctly predict the drug clearance for some cases, the definition of the chemotherapy schedule can also carry some inaccuracies when defined by these methods. For instance, the Body Surface Area ($BSA$), which is still an extensively used variable for definition of the drug dose, has not shown an acceptable correlation with the $AUC$ and clearance of doxorubicin [18] or cisplatin [19], which are common drugs employed in the cancer treatment. As a complement, the present model is able to predict the spatio-temporal evolution of drug concentration in the vessels, extracellular and intracellular space, as well as the transport and reaction mechanisms of the chemotherapeutic drug in electroporated and vasoconstricted tissues. Accordingly, the present model could assist the initial evaluation of the effects of the chemotherapy doses and schedule on the drug distribution, concentration and exposure along the vessel and tissues during the whole treatment prior to the application of the therapy. The scaling to macroscopic domains with complex vascular and lymphatic network, the consideration of more specific reaction and transport mechanisms arising in several subdomains (like the endothelial wall), the inclusion of cell death models by electroporation and cytotoxic effects, among others, are challenges that represent opportunities of improvement of the present model. These lines have been included in the Conclusion section of new version of the manuscript (***Page 40 of the new manuscript***).

- **Comment 6.**

In the Discussion section the authors talk about protein content, while this is not included in their model. What they include is protein association to drug, which is not the same.

**Response.**

Certainly, protein content and protein association are two different concepts, but not disconnected. In the Discussion Section, the “protein content” is mentioned to contextualize that lower association between drug and proteins is expected for patients with hypoproteinemia due to the lower binding site concentration, $C_{0}$, and this could have implications in the chemotherapy efficiency since the free intracellular drug is able to escape from the cell towards the interstitial space.

- **Comment 7.**

The work is difficult to read. It has too many details, too many acronyms, too many repetitions of the variables and too many uninteresting results. The authors should consider to select 2-3 combinations of their variables, explain and discuss them and leaving the rest for the SuppMat.

**Response.**

Firstly, the use of acronyms in the text is essential to simplify the writing about the presence and interaction of the reaction and transport mechanisms ($RTMs$) along the tissue at each time instant. It is not convenient to write this as mere text since this would only unnecessarily increase the length and complexity of the manuscript. A nomenclature table was added with the meaning of these acronyms. On the other hand, the analysis cases are all interconnected, and leaving any of them as supplementary material may complicate the manuscript presentation and reading. In the new version of the manuscript, efforts were made to improve the writing of ***Section 5, Results and Discussion***.

- **Comment 8.**

The introduction is a paragraph of true introduction followed by a long-winded description of Figure 1. This description looks like a review and summarises methods used in other works, but it’s unclear how the present work uses them. This description should go to SuppMat and leave here one single summarised paragraph (same for Figure 2 and 3). Also, a lot of information is given that is completely unnecessary for the understanding of the paper (DNA ability to go through electroporated holes?)

**Response.**

Following the Reviewer’s recommendations, most of the information about the reaction and transport mechanisms ($RTMs$) in the Introduction section has been moved to the Supplementary Material. Only the basic explanation of these mechanisms and the models used to consider the effects of electric pulses application on them have been kept in the new version of the manuscript ***page 2 and 3***.

- **Comment 9.**

The paper lacks a methods section. The section “Data and Simulation campaign” is just a description of the values and $PK$ models used. There is no way that anyone can reproduce this work with only that information. The authors have not explained how they have simulated their equations (what solver or tool), what they have used to plot the results, how does the Boolean rules connect to the Concentration equations.

**Response.**

The mathematical models and numerical methods used to obtain the concentration fields ($C_{1}$, $C_{2}$ and $C_{3}$) of the electroporated and vasoconstricted tissues are summarized in Section 2 of the manuscript, while the Boolean model used to determine the presence and interaction of the reaction and transport mechanisms ($RTMs$) based on the spatio-temporal behavior of the ratios $C_{2}/C_{1}$, $C_{3}/C_{1}$ and $C_{3}/C_{2}$ is developed in Section 3. To provide a clearer description of the methods used in the present work and ensure the reproducibility of the results, Section 4 has been expanded in the new version of the manuscript (***Pages 17 and 18 of new version of manuscript***), aiming to establish a clearer connection with Sections 2 and 3. Additionally, a new figure has been created to show the workflow of the present research (***Figure 7 of new version of manuscript***).

- **Comment 10.**

The description of the results does not provide insights into the problem without any talk about synergies among variables under study and with many self-evident phrases such as:
“As can be observed, the axial distribution of 3 is not uniform for the three blood speeds, indicating that the association/dissociation rate is not the same for a set of points equidistant from the border” when the bloodstream has an inlet and outlet
“According to the numerical results, the for the One-short Tri-exponential () and Mono-exponential profile () can differ each other for a given combination of and .” Of course different $PK$ dynamics have different results
MPK model “leads to a higher possibility of increased the final cell death since drug resides inside the cells during more time.” This is evident when drugs act killing cells. In fact, authors should read about the most common drug resistance mechanisms.

**Response:**

As the reviewer points out, along the manuscript some phrases could be redundant to readers familiar with the biophysics of chemotherapeutic drug transport. But this may not be the case for other kind of readers. Some of these sentences have been rewritten, but others have not as they may be necessary to provide some clarity. For example, not all cell death models take into account the residence time of the drug in the intracellular space. In general, the modeling of cell death due to the cytotoxicity of the anticancer drugs has been conceived in different ways. In the particular case of doxorubicin, some authors [20] have proposed that cell death at a given time instant after the beginning of therapy depends solely on the cumulative exposure to the drug. This hypothesis has been refuted in other studies. For example, according to [21], the shape of the drug concentration vs. time curve also influences cell death, while some models only consider the total drug concentrations [22], [23]. On the other hand, other models take into account the influence of the internalized drug on cell death [22], according to which the residence time of the drug in the intracellular space can indeed increase the cell-kill capacity of the treatment.

On the other hand, the present model that not still account for common drug resistance mechanism. In future works, , it could be necessary to enhance the model by including the mechanisms of drug resistance of tumor cells, such as: (1) tumor heterogeneity due to mutations, deletions, chromosomal rearrangements, among others [24], [25], (2) inactivation of the medication by alteration of its molecular characteristics when binding to proteins [24], [25], (3) uncontrolled release of certain medications (Doxorubicin, Vinblastine, and Taxol) into the extracellular space due to certain substances like chloride transported by ATP-dependent carriers [24], [26], (4) changes in drug metabolism by enzymes [24], [27], (5) secondary mutations in cancer cells such as topoisomerase II [24], [28], (6) processes of DNA damage and repair, especially in platinum compounds and doxorubicin [24], [29].

- **Comment 11.**

The present “Results and discussion” section is a dry section of pure description of the figures. There is no discussion, there is no connection of these results with state of the art.

**Response.**

The computational development of the present manuscript comprises two stages: (1) the solution of the species conservation equations of an electroporated and vasoconstricted tumor cord domain by $GMAPS$ and (2) the development of a Boolean model to explore the interaction between the principal reaction and transport mechanisms ($RTMs$) of drugs in electrochemotherapy, which is the principal objective of the present research. Results of the first stage are quantitative. As mentioned in ***Page 8*** of the manuscript, an accuracy and convergence analysis of the $GMAPS$ code employed in this stage was carried out in a recent work of the Authors [17], where the simulations of [3], [4] were replicated using the developed *GMAPS* code, and the results were compared in terms of the $L^{2}$ relative error norm. Specifically, the following results were considered in [17] for the validation of the present $GMAPS$ code: radial profiles of extracellular and bound intracellular concentrations at several time instants, and surface response of exposure to bound intracellular drug in terms of the radial and axial coordinates. Satisfactory results were obtained in the referred work [17], confirming the reliability of the $GMAPS$ code.

The second stage of the present computational development gives qualitative results, focused on determining the presence and relative importance of the different reaction and transport mechanisms ($RTMs$) arising in the vessels, extracellular and intracellular space. Since we do not currently have experimental facilities to validate these results under the same simulation conditions, we sought recent literature to compare some qualitative results of the present work, finding some similar trends and behaviors for non-electroporated tissues ***(Section 5.1.3, Pages 27 and 28 of new manuscript)***, and electroporated tissues ***(Section 5.2.3, Pages 32 and 33 of new manuscript, and Section 5.3.3, Pages 35-36 of new manuscript)***.

- **Comment 12.**

The paper lacks a proper conclusions section. The present “Conclusions” section is a summary of the results and handwaved connections to clinical usefulness.

**Response.**

To improve the contextualization of the conclusions, a short summary of the results is initially included in this section. Afterwards, possible utility of the present model at the clinical context is discussed in this section ***(Page 39-41 of new manuscript)***.

- **Comment 13.**

The text is not clear in what is the added value of this work in respect to the state of the art. What is the gap in the state of the art that this work fills?

**Response.**

The following was added in the Introduction of new manuscript to identify the main contribution of this paper with respect to previous works ***(Page 4-5 of new manuscript)***:

*“As detailed in the Supplementary Material, the transport and reaction phenomena involved in the drug passage from the circulatory system to the intracellular space have been studied at both macroscopic and microscopic scales by experiments and numerical simulations. The cited in-silico studies have used mainly meshed techniques (Finite Element Methods, Finite Volume Method, Finite Difference Method, etc.). Particularly, this research group have developed several in-silico tools using meshless and mesh-boundary techniques to predict the extracellular (*$C_{1}$*), free intracellular (*$C_{2}$*) and bound intracellular concentration (*$C_{3}$*) of drug in electroporated and vasoconstricted tissues at both microscopic [17], [30], [31] and macroscopic scale [32], [33]. To the best of our knowledge, the evolution of the reaction and transport mechanisms (*$RTMs$*) considering the spatio-temporal behavior of these concentration fields (*$C_{1}$*_,_* $C_{2}$ *and* $C_{3}$*) has been tackled using merely descriptive analyses, but not a rigorous logical inference analysis. Accordingly, one of the main contributions of this work lies on the development of a Boolean model to accurately establish the presence, interaction and rates of the* $RTMs$ *in electroporated and vasoconstricted tissues applying logical inference laws for all possible combinations of the ratios* $C_{2}/C_{1}$*,* $C_{3}/C_{1}$ *and* $C_{3}/C_{2}$*, which in turns are retrieved from numerical simulations using a* $GMAPS$ *code previously developed, calibrated and validated [17]”.*

The main contribution of this work has been also declared in the Abstract of new Manuscript.

- **Comment 14.**

The text in unclear in respect to what are the precise contributions of this work and what has been inherited from the authors previous work (ref 7). The present work state at the end of the introduction “The principal purpose of the present research is to investigate the influence of the combined input parameters (, , ) on the interaction between the aforementioned reaction and transport mechanisms in the context of electro-chemotherapeutic treatments, aiming to propose potential clinical implications resulting from this combination.” How is this different from ref 7?

**Response.**

Previous work [17] is devoted to the development, validation and calibration of the *GMAPS* code used here. In that work, the researches of Groh [3] and Hubbard [4] were replicated for validation purposes, and a convergence analysis on the number of collocation points, time step size and relaxation factor was carried out for the model calibration. The $GMAPS$ code presented in [17] was used here to obtain the concentration fields ($C_{1}$*,* $C_{2}$ and $C_{3}$), and then compute the ratios $C_{2}/C_{1}$*,* $C_{3}/C_{1}$ and $C_{3}/C_{2}$*,* at several points and time instants. These ratios are the inputs of the Boolean model developed here that allows predicting the presence and interaction of the different reaction and transport mechanisms (extravasation, cellular internalization, cellular externalization, association, dissociation, positive and negative extracellular transport, etc.) involved in the drug passage from the circulatory system towards the cellular space. The main contribution of this work has been also declared in the Abstract of new Manuscript.

- **Comment 15.**

The authors do not need to cite 3 or 4 papers each time they present a methods. This could be cut down to 1 or 2. An example of this is page 14 when refs 70-74 are used to cite the approach used.

**Response.**

Following the Reviewer’s recommendations, number of references were reduced in the ***Section 2 of the new manuscript***.

- **Comment 16.**

Even many times these references are not even using the method they are being cited for. An example of this is page 16 when refs 76-79 are cited but none of them are using Boolean rules as the authors are using (to discretise a continuous behavior). Another one is refs 80-83, which of the three talks about the “design potential electro-chemotherapeutics protocols”.

**Response.**

These references are cited just for illustrating the usefulness of the Boolean modelling in the analysis of biological systems, not matter the approach they are using. These works are focused on generating synchronous, asynchronous and probabilistic networks, and their corresponding state graphs; as in the present work, they are based on using Boolean algebra rules. Boolean schemes to envisage the presence and interactions of $RTMs$ for all combinations of the concentration ratios ($C_{2}/C_{1}$*,* $C_{3}/C_{1}$ and $C_{3}/C_{2}$*,*) are generated here ***(Figures 4 and 5 of the manuscript)***. In the case of references 42-45 of the new version of the manuscript, they are cited to evidence the suitability of Boolean modeling in revealing the dynamic of several cancer therapies, which could be useful to “design potential electro-chemotherapeutics protocols”. However, this last part has been removed in the new version of the manuscript to avoid any confusion ***(Page 9 of new manuscript)***.

- **Comment 17.**

References are needed for these sentences:
“The protein content of cancer patients has a significant role in the side effects of the treatment.” “Patients with low protein levels, known as hypoproteinemia, are more susceptible to side effects and require lower drug doses.”

**Response.**

Several references were added for these sentences in the new version of the manuscript ***(Page 40 of new manuscript)***.

- **Comment 18.**

The figure footers need to be explanatory. The authors barely describe the figures. For instance in Figure 6 the acronyms should be explained there, not in the text.

**Response.**

Following the Reviewer’s recommendation, the figure captions were improved in the version of the manuscript.

- **Comment 19.**

In Figures 7 forward, the panels are the same patch of tissue at different times. The authors should remove the space in-between panels and present them as facets of a single figure. In Figures 7 forward, the authors should use a common legend for all the facets. This would help understand better the colours across time points (as in Fig 9).

**Response.**

Following the Reviewer’s recommendation, the quality, distribution and captions of all figures were improved in the new version of the manuscript. White spaces were considerably reduced, and legends sizes were increased in order to show more clearly the meaning of colors in the contour plots. However, it is inconvenient to unify the legend of all subplots for a given figure because the presence and interactions of reaction and transport mechanisms (RTMs) can change from one subplot to another, as well as the maximum and minimum values of the contour variables $C_{2}/C_{1}$ and $C_{3}$.

- **Comment 20.**

Use page number and line numbers. It helps reviewing papers.

**Response:**

Line numbers were added in new version of manuscript.

***REVIEWER 2.***

- **Comment 1.**

In the first section “when it occurs from the vessel to the interstitial space (extravasation), and as − or when it occurs in the opposite direction (lymphatic drainage”) lymphatic drainage was mentioned but based on the mathematical formulation it is not completely clear how was this implemented during the simulation. Also, Figure 1. Implies a localized drainage compared to the description of the model (Figure 2.), and the boundaries of the tumor cord (Figure 6.)

**Response.**

Lymphatic drainage is an important mechanism to return the drug back to the circulatory system by means of lymphatic vessels, which are usually more permeable than the capillary vessels. Some solid tumors do not have an effective lymphatic system for the drug clearance, especially in distal zones. Considering its importance, this transport mechanism is represented in Figure 1. However, the tumor cord approach, whose domain is represented in Figures 2 and 8 of new manuscript, is focused on studying the drug penetration from the capillary vessels towards the less irrigated zones of the tissue [3], [4], [34], and do not account explicitly for the lymphatic drainage. The present model can consider the return of drug from the interstitial space towards the circulatory system by reverse concentration gradients, namely, when $C_{1}$ exceeds $C_{v}$ at the tissue-vessel wall interface, however a different domain for the lymphatic vessels is not considered in the tumor cord domain. Further research can be focused on modifying the tumor cord model to incorporate explicitly the lymphatic vessels.

- **Comment 2.**

The diffusion back to the blood vessel, once the free plasma concentration decreases below the tumor tissue’s free drug concentration is not represented in either the Figures or the text. If it is present would it be possible to highlight it? If not present, adding this calculation to the Boolean model ( $C_{v}/C_{1}$ – increasing or decreasing ) would be highly relevant (especially regarding the optimization of the timing of the electroporating magnetic field in relation to drug accumulation in the cancer tissue).

**Response.**

To address this comment, a new section devoted to the analysis of the $C_{v}/C_{1}$ ratio has been added in the new version of the manuscript ***(Section 5.4, Pages 36 and 37 of new manuscript)***, showing the time evolution of this ratio until the first hour of the treatment.

- **Comment 3.**

Is the Tchemo referring simply to the length of the simulation evaluated or does it have further meaning? It is not described in the text.

**Response.**

$T_{chemo}$ is the simulation time of electro-chemotherapy (24 hours in this case). The meaning of the variable is shown in nomenclature table ***(Page 45)*** and has been included in text of new manuscript in ***Page 18***.

- **Comment 4.**

In section 1 it says: “The appropriate choice of parameters such as the voltage level, pulse form, spacing, length, and frequency, as well as number and duration of electroporation treatments, and electrodes location is crucial for achieving the primary objective of reversible electroporation (), namely, to augment the cell membrane permeability and facilitate the drug translocation towards the intracellular space while maintaining the cell viability [2], [3], [4].” But in Figure 6. The location magnetic field/poles are not represented.

**Response.**

When simulations are conducted at the macroscopic scale (tissue domain of order of millimeters), the electric field magnitude, $E$, shall be considered as variable and its distribution highly depends on the electrode positioning [1], [32], [33], [35]. On the other hand, for simulations at the microscopic scale (Representative Unitary Cell), as the ones considered in the present work, bearing in mind that the characteristic length of the tumor cord domain (order of micrometers in this case) is usually from one to three orders of magnitude smaller than such of the tissue domain, a constant electric field magnitude, $E$, can be supposed, and electrode positioning is not relevant in the simulations [3], [4], [17], [30], [31]. This explanation is added in ***Page 9 of new version of the manuscript*** for sake of clarity.

- **Comment 5.**

In section 6 :“Facilitating the interstitial transportation of drug ( +) to cancer cell receptors is advantageous for Antibody-Drug Conjugate () therapies, as the drug is only released upon internalization”. It is not clear how can you optimize the model for drugs where the drug entry to the cell is ensured by single-use proteins (e.g.: one surface protein allows one $ADC$ molecule to enter the cell and then its degraded).

**Response.**

The tumor cord model used in the present work does not account for the targeted transport of $ADC’s$ towards cell surface antigens. The $ADC$ therapy is only mentioned in the discussion of the manuscript to highlight the importance that the stimulation of the positive extracellular transport ($ECT+$) towards the cell receptors could have in the $ADC$ therapy. The modelling of the directional motion of monoclonal antibodies linked to cytotoxic drugs towards target antigens, with the subsequent cellular internalization and drug release, is a complex phenomenon that is out of the scope of the present research, but can be considered in future works.

- **Comment 6.**

It would be intriguing to see the doxorubicin content of the tumor cord in the different fluid compartments (intravasal / Extracellular / Intracellular free /intracellular protein bound ) since the color scales – rightfully so – are cannot be synchronised. IT would help with the contextualization of the drug distribution within the model in the different time points. Figures 9-10; Figures 12-17.

**Response.**

Please notice that we are using a continuum model instead of a compartmental model, and it is not possible to distinguish geometrically between the extracellular and intracellular space in the tumor cord domain. We preferred to report the contour plots of the ratio between the free intracellular and extracellular concentrations ($C_{2}/C_{1}$) and the bound intracellular concentration ($C_{3}$) because they account for the interaction between internalization, externalization, association and dissociation of drug following the logical rules of the Boolean model developed here. The drug extracellular concentration ($C_{1}$), free intracellular concentration ($C_{2}$) and intravasal concentration ($C_{v}$) alone do not allow to infer so much about the presence, interactions and rates of reactions and transport mechanisms ($RTMs$). Coherently with Comment 2 of this Reviewer, ratio $C_{v}/C_{1}$ is another important parameter since this account for drug extravasation and drug drainage from and towards the capillary vessels, respectively. Bearing this in mind, an analysis of this ratio for the first hour of treatment has been added in ***Section 5.4, Pages 36 and 37 of new manuscript***.

- **Comment 7.**

One typo was noticed in the abstract
a. “Using in-house computational tools, this works investigates” - » “Using in-house computational tools, this work investigates.

**Response.**

This is corrected in the new version of the manuscript.

***REVIEWER 3.***

This study investigates the effects of electroporation (both its absence and varying intensity), different bloodstream velocities, and two pharmacokinetic profiles on the reaction and transport mechanisms in electro-chemotherapeutic treatment. These effects are analyzed through numerical simulations of the tumor cord model using the GMAPS scheme. Subsequently, a Boolean model is employed to analyze the interactions between the reaction and transport mechanisms by examining the evolution of ratios among different concentrations: extracellular drug concentration, free intracellular drug concentration, and bound intracellular drug concentration. Some points should be considered before further consideration of the manuscript, as follows;

- **Comment 1.**

The abstract should not include background information. The authors should move the initial sentences of the abstract to the introduction. The abstract should concisely describe the purpose of the research, the novelty of the work, the key results, and the conclusion.

**Response.**

Following the Reviewer’s recommendation, the background information was eliminated from the abstract in the new version of manuscript. Moreover, the main contribution of the present work was explicitly declared as the development of a Boolean model to determine the presence, interaction and rates of the reaction and transport mechanisms $(RTMs)$ of drug in electro-chemotherapeutic treatments based on the comparison of the spatio-temporal evolution of the drug concentration ratios ($C_{2}/C_{1}$, $C_{3}/C_{1}$ and $C_{3}/C_{2}$).

- **Comment 2.**

The authors need to clearly articulate their study's contributions in comparison to previous research in both the abstract and the final paragraph of the introduction. They should emphasize any distinctions from various perspectives, including differences in the mathematical model, the incorporation of additional biological factors, the increased complexity of the study's physics, and the investigation of new subjects.

**Response.**

Following the recommendation of another Reviewer, the brief state of the art about the study of the reaction and transport mechanisms ($RTMs$) of chemotherapeutic drugs, originally included in the Introduction of the manuscript, was moved to the Supplementary material. In that Supplementary material, it can be evidenced that the $RTMs$ have been studied at both macroscopic and microscopic scales by experiments and numerical simulations. The cited in-silico studies have used mainly meshed techniques (Finite Element Methods, Finite Volume Method, Finite Difference Method, etc.). As mentioned in several parts of the manuscript, the present work makes use of previous computational developments of the Authors, where several in-silico tools, based on meshless and mesh-boundary techniques, were used to predict the extracellular ($C_{1}$), free intracellular ($C_{2}$) and bound intracellular concentrations ($C_{3}$) of drug in electroporated and vasoconstricted tissues at both microscopic [17], [30], [31] and macroscopic scale [32], [33]. To the best of our knowledge, the evolution of the reaction and transport mechanisms (RTMs) considering the spatio-temporal behavior of these concentration fields ($C_{1}$*_,_* $C_{2}$ and $C_{3}$) has been tackled using merely descriptive analysis, but not a rigorous logical inference analysis. Accordingly, one of the main contributions of this work lies on the development of a Boolean model to accurately establish the presence, interaction and rates of the RTMs applying logical laws for all possible combinations of the ratios $C_{2}/C_{1}$, $C_{3}/C_{1}$ and $C_{3}/C_{2}$, which in turns are retrieved from numerical simulations using a GMAPS code previously developed, calibrated and validated [17]. This is specified in ***Page 4 and 5 of new version of manuscript*** (final paragraph of the Introduction) and summarized in the Abstract.

- **Comment 3.**

The quality of the contours shown in Figures 7-17 and in the supplementary file needs improvement. Currently, the readability of these contours is suboptimal.

**Response.**

Following the Reviewer’s recommendation, the quality and distribution of all figures was improved in the new version of the manuscript. White spaces were considerably reduced, and legends size was increased in order to show more clearly the meaning of colors in the contour plots.

- **Comment 4.**

After introducing abbreviations at their first mention, it is recommended to consistently use them thereafter and avoid reintroducing the full terms. The authors should use the abbreviations for the terms once they have been introduced, if they intend to abbreviate.

**Response.**

This was revised and corrected in the new version of the manuscript.

- **Comment 5.**

It is recommended that the authors incorporate the latest research into the introduction. For example, the study found at <https://www.mdpi.com/2072-6694/15/22/5464> explores multi-scale modeling of drug delivery into solid tumors, addressing transport along blood vessels, transvascular transport, and extravascular transport. This research also examines the effects of the microvascular network and the changes in transport properties induced by vascular normalization from anti-angiogenic therapy on the convective and diffusive transport mechanisms of drug delivery.

**Response.**

This reference has been included in ***Page 3 of new version of the manuscript***.

- **Comment 6.**

It is recommended to include a flowchart of the current numerical study, which summarizes the application of the numerical method, the Boolean model, and the various scenarios examined (excluding electroporation, with two different electroporation values, different bloodstream velocities, and pharmacokinetic profiles). Providing such a flowchart will enhance the clarity and comprehensibility of the study for readers.

**Response.**

Following the Reviewer’s recommendation, a workflow of the present research has been included in ***Figure 7*** and referred on the ***Section 4*** of new version of the manuscript.

***REVIEWER 4.***

The paper "In-silico tool based on Boolean Networks and meshless simulations for prediction of
reaction and transport mechanisms in the systemic administration of chemotherapeutic
drugs." reports on systemic administration of chemotherapeutic drugs comprises some reaction and transport mechanisms ().

- **Comment 1.**

The software / computational tools have already been previously reported:
F. M. Vélez Salazar and I. D. Patiño Arcila, “Influence of electric field, blood velocity, and pharmacokinetics on electrochemotherapy efficiency,” Biophys J, vol. 122, no. 16, 2023, doi:10.1016/j.bpj.2023.07.004. The novelty in this manuscript is "Subsequently, a Boolean model is proposed here to analyze the interaction between based on the comparison of the spatio-temporal evolution of concentration ratios." So is then the Boolean model available for the interested reader?

**Response.**

The development of the Boolean model was presented in detail in **Section 3** of the manuscript for reproduction purposes. If manuscript is accepted, algorithm will be available on demand.

- **Comment 2.**

The model is presented and impressively complex. Now, chemotherapy is routinely administered without such a complex model, can you explain the reader, why he/she should then use the
complex model of yours instead of simpler heuristics (e.g. dosage per weight, per surface, combinations of such parameters etc. etc.)?

**Response.**

It is worth-mentioning that the present model is not intended to replace the Heuristic methods commonly used for dosing of anticancer drugs (weight-based dosing, body surface area dosing, fixed-dose prescribing, AUC-based dosing, among others), but to complement these methods. Heuristic methods have been defined based on clinical practice considering several aspects such as: type of drug, type and subtype of cancer, stage of cancer, previous health treatments, current patient’s medications and mainly, the patient’s characteristics (age, weight, height, health problems, among others). One of the main drawbacks of these methods is their poor relationship with the pharmacokinetic parameters of chemotherapeutic drugs (loading and maintenance dose, injection time, decay rates, among others), affecting the accuracy in the prediction of the drug exposure and drug clearance, and consequently, the individualization capacity of the therapy. Moreover, since some heuristic models are not able to correctly predict the drug clearance for some cases, the definition of the chemotherapy schedule can also carry some inaccuracies when defined by these methods. For instance, the Body Surface Area ($BSA$), which is still an extensively used variable for definition of the drug dose, has not shown an acceptable correlation with the AUC and clearance of doxorubicin [ref1] or cisplatin [ref2], which are common drugs employed in the cancer treatment. As a complement, the present model is able to predict the spatio-temporal evolution of drug concentration in the vessels, extracellular and intracellular space, as well as the transport and reaction mechanisms of the chemotherapeutic drug in electroporated and vasoconstricted tissues. Accordingly, the present model could assist the initial evaluation of the effects of the chemotherapy doses and schedule on the drug distribution, concentration and exposure along the vessel and tissues during the whole treatment prior to the application of the therapy. These lines have been included in the Conclusion section of new version of the manuscript (***Page 40 of the new manuscript***).

- **Comment 3.**

For the non-specialist reader there are no experimental data visible. Also the supplement seems to have only even more simulations. Can you please explain is your model validated by experimental data or this is not yet the case?

**Response.**

The computational development of the present manuscript comprises two stages: (1) the solution of the species conservation equations of an electroporated and vasoconstricted tumor cord domain by $GMAPS$ and (2) the development of a Boolean model to explore the presence and interaction of the principal reaction and transport mechanisms ($RTMs$) of drugs in electrochemotherapy, which is the principal objective of the present research. Results of the first stage are quantitative. As mentioned in ***Page 8*** of the manuscript, an accuracy and convergence analysis of the $GMAPS$ code employed in this stage was carried out in a recent work of this research group [17], where the simulations of [3], [4] were replicated using the developed $GMAPS$ code, and the results were compared in terms of the $L^{2}$ relative error norm. Specifically, the following results were considered in [17] for the validation of the $GMAPS$ code: radial profiles of extracellular and bound intracellular concentrations at several time instants, and surface response of exposure to bound intracellular drug in terms of the radial and axial coordinates. Satisfactory results were obtained in the referred work [17], confirming the reliability of the $GMAPS$ code used here.

The second stage of the present computational development gives qualitative results, focused on determining the presence and relative importance of the different reaction and transport mechanisms ($RTMs$) arising in the vessels, extracellular and intracellular space. Since we do not currently have experimental facilities to validate these results under the same simulation conditions, we sought recent literature to compare some qualitative results of the present work, finding some similar trends and behaviors for non-electroporated tissues ***(Section 5.1.3, Pages 27 and 28 of new manuscript)***, and electroporated tissues ***(Section 5.2.3, Pages 32 and 33 of new manuscript, and Section 5.3.3, Pages 35-36 of new manuscript)***.

- **Comment 4.**

Can you educate the reader of an actual, convincing use case of your model in the clinic?

**Response.**

As previously mentioned, the validation of the $GMAPS$ code employed here to obtain the concentration fields ($C_{1}$, $C_{2}$ and $C_{3}$) was presented in a previous work of this research group [17], obtaining satisfactory results. On the other hand, similar trends and behaviours for the qualitative results of the Boolean model have been reported in recent works, as discussed in ***Sections 5.1.3, 5.2.3 and 5.3.3 of new version of the manuscript***.

This is essentially an exploratory work, but all parameters were obtained from existing literature. Accordingly, the three levels of the electric field magnitude, $E=[0 kV/m, 46 kV/m, 70 kV/m]$, correspond to a non-electroporated tissue, reversible and irreversible thresholds of a liver tissue [1], [2]. The parameters of both pharmacokinetic profiles ($MPK$ and $TPK$) for doxorubicin were obtained from Groh et al. [3], whereas parameters of the bloodstream velocity ($\lambda_{inl}$) from Hubbard et al [4]. However, we did not find a well-characterized clinical case reporting all input parameters needed to feed the model, and the Authors do not currently have experimental facilities to carry out in-vitro or in-vivo experiments under the same conditions.

- **Comment 5.**

Or is the usage thought more for fundamental biophysics research (in this case, please explain for which biophysics question the model is useful).

**Response.**

At this initial stage, the Boolean model is thought to address a biophysical issue. As detailed in the Supplementary material of the new manuscript, the transport and reaction phenomena involved in the drug passage from the circulatory system to the intracellular space is a problem that has been studied at both macroscopic and microscopic scales by experiments and numerical simulations. Particularly, this research group have developed several in-silico tools to predict the extracellular ($C_{1}$), free intracellular ($C_{2}$) and bound intracellular concentrations ($C_{3}$) of drug in electroporated and vasoconstricted tissues at both microscopic [17], [30], [31] and macroscopic scales [32], [33]. However, to the best of our knowledge, the evolution of the reaction and transport mechanisms ($RTMs$) considering the spatio-temporal behavior of these concentration fields ($C_{1}$_,_ $C_{2}$ and $C_{3}$) has been tackled using merely descriptive analysis, but not a rigorous logical inference analysis. Accordingly, this problem is tackled here by developing a Boolean model to accurately establish the presence, interaction and rates of the $RTMs$ applying logical inference laws for all possible combinations of the ratios $C_{2}/C_{1}$, $C_{3}/C_{1}$ and $C_{2}/C_{3}$, which in turns are retrieved from numerical simulations using a $GMAPS$ code previously developed, calibrated and validated [17]. To better identify the contribution of the present work, this is specified in *Page 4-5* of new manuscript (last paragraph of Introduction).

**REFERENCES.**

[1]	B. Boyd and S. Becker, “Macroscopic Modeling of In Vivo Drug Transport in Electroporated Tissue,” *J Biomech Eng*, vol. 138, no. 3, pp. 1–12, Mar. 2016, doi: 10.1115/1.4032380.

[2]	Davorka. Šel, D. Cukjati, D. Batiuskaite, T. Slivnik, L. M. Mir, and D. Miklavčič, “Sequential Finite Element Model of Tissue Electropermeabilization,” *IEEE Trans Biomed Eng*, vol. 52, no. 5, pp. 816–827, May 2005, doi: 10.1109/TBME.2005.845212.

[3]	C. M. Groh *et al.*, “Mathematical and computational models of drug transport in tumours,” *J R Soc Interface*, vol. 11, no. 94, p. 20131173, May 2014, doi: 10.1098/rsif.2013.1173.

[4]	M. E. Hubbard, M. Jove, P. M. Loadman, R. M. Phillips, C. J. Twelves, and S. W. Smye, “Drug delivery in a tumour cord model: a computational simulation,” *R Soc Open Sci*, vol. 4, no. 5, p. 170014, May 2017, doi: 10.1098/rsos.170014.

[5]	T. A. Ahmed, “Pharmacokinetics of Drugs Following IV Bolus, IV Infusion, and Oral Administration,” in *Basic Pharmacokinetic Concepts and Some Clinical Applications*, 2015. doi: 10.5772/61573.

[6]	J. Fan and I. A. M. De Lannoy, “Pharmacokinetics,” 2014. doi: 10.1016/j.bcp.2013.09.007.

[7]	A. W. El-Kareh and T. W. Secomb, “A mathematical model for comparison of bolus injection, continuous infusion, and liposomal delivery of doxorubicin to tumor cells,” *Neoplasia*, vol. 2, no. 4, 2000, doi: 10.1038/sj.neo.7900096.

[8]	J. Robert, A. Illiadis, B. Hoerni, J. P. Cano, M. Durand, and C. Lagarde, “Pharmacokinetics of adriamycin in patients with breast cancer: Correlation between pharmacokinetic parameters and clinical short-term response,” *Eur J Cancer Clin Oncol*, vol. 18, no. 8, pp. 739–745, 1982, doi: 10.1016/0277-5379(82)90072-4.

[9]	M. T. McKenna *et al.*, “A Predictive Mathematical Modeling Approach for the Study of Doxorubicin Treatment in Triple Negative Breast Cancer,” *Sci Rep*, vol. 7, no. 1, 2017, doi: 10.1038/s41598-017-05902-z.

[10]	C. M. Camaggi, R. Comparsi, E. Strocchi, F. Testoni, B. Angelelli, and F. Pannuti, “Epirubicin and doxorubicin comparative metabolism and pharmacokinetics - A cross-over study,” *Cancer Chemother Pharmacol*, vol. 21, no. 3, 1988, doi: 10.1007/BF00262774.

[11]	C. Rossi *et al.*, “Doxorubicin distribution in human breast cancer.,” *Cancer Treat Rep*, vol. 71, no. 12, 1987.

[12]	I. Druzhkova *et al.*, “Effect of Collagen Matrix on Doxorubicin Distribution and Cancer Cells’ Response to Treatment in 3D Tumor Model,” *Cancers (Basel)*, vol. 14, no. 22, 2022, doi: 10.3390/cancers14225487.

[13]	S. Kok-Yong and L. Lawrence, “Drug Distribution and Drug Elimination,” in *Basic Pharmacokinetic Concepts and Some Clinical Applications*, 2015. doi: 10.5772/59929.

[14]	F. Mpekris, M. Panagi, A. Charalambous, C. Voutouri, and T. Stylianopoulos, “Modulating cancer mechanopathology to restore vascular function and enhance immunotherapy,” Jul. 16, 2024, *Cell Press*. doi: 10.1016/j.xcrm.2024.101626.

[15]	T. Stylianopoulos and R. K. Jain, “Combining two strategies to improve perfusion and drug delivery in solid tumors,” *Proc Natl Acad Sci U S A*, vol. 110, no. 46, 2013, doi: 10.1073/pnas.1318415110.

[16]	S. Whitaker, *The Method of Volume Averaging*, vol. 13. in Theory and Applications of Transport in Porous Media, vol. 13. Dordrecht: Kluwer Academics Publisher, 1999. doi: 10.1007/978-94-017-3389-2.

[17]	F. M. Vélez Salazar and I. D. Patiño Arcila, “Influence of electric field, blood velocity, and pharmacokinetics on electrochemotherapy efficiency,” *Biophys J*, vol. 122, no. 16, 2023, doi: 10.1016/j.bpj.2023.07.004.

[18]	G. Freyer *et al.*, “Population pharmacokinetics of doxorubicin, etoposide and ifosfamide in small cell lung cancer patients: Results of a multicentre study,” *Br J Clin Pharmacol*, vol. 50, no. 4, pp. 315–324, 2000, doi: 10.1046/j.1365-2125.2000.00269.x.

[19]	F. De Jongh *et al.*, “Body-Surface Area–Based Dosing Does Not Increase Accuracy of Predicting Cisplatin Exposure,” *Clin Oncol*, 2001, [Online]. Available: https://pubmed.ncbi.nlm.nih.gov/11533095/

[20]	S. Ozawa, Y. Sugiyama, Y. Mitsuhashi, T. Kobayashi, and M. Inaba, “Cell killing action of cell cycle phase-non-specific antitumor agents is dependent on concentration--time product,” *Cancer Chemother Pharmacol*, vol. 21, no. 3, pp. 185–190, May 1988, doi: 10.1007/BF00262767.

[21]	J. Lankelma, R. Fernández Luque, H. Dekker, and H. M. Pinedo, “Simulation model of doxorubicin activity in islets of human breast cancer cells,” *Biochim Biophys Acta Gen Subj*, vol. 1622, no. 3, pp. 169–178, 2003, doi: 10.1016/S0304-4165(03)00139-9.

[22]	A. W. El-Kareh and T. W. Secomb, “Two-mechanism peak concentration model for cellular pharmacodynamics of doxorubicin,” *Neoplasia*, vol. 7, no. 7, pp. 705–713, 2005, doi: 10.1593/neo.05118.

[23]	N. J. Millenbaugh, M. G. Wientjes, and J. L. S. Au, “A pharmacodynamic analysis method to determine the relative importance of drug concentration and treatment time on effect,” *Cancer Chemother Pharmacol*, vol. 45, no. 4, pp. 265–272, 2000, doi: 10.1007/s002800050039.

[24]	B. Mansoori, A. Mohammadi, S. Davudian, S. Shirjang, and B. Baradaran, “The Different Mechanisms of Cancer Drug Resistance: A Brief Review,” *Adv Pharm Bull*, vol. 7, no. 3, pp. 339–348, 2017, doi: 10.15171/APB.2017.041.

[25]	B. J. Druker *et al.*, “Activity of a specific inhibitor of the BCR-ABL tyrosine kinase in the blast crisis of chronic myeloid leukemia and acute lymphoblastic leukemia with the Philadelphia chromosome,” *N Engl J Med*, vol. 344, no. 14, pp. 1038–1042, Apr. 2001, doi: 10.1056/NEJM200104053441402.

[26]	R. C. Cumming, J. Lightfoot, K. Beard, H. Youssoufian, P. J. O’brien, and M. Buchwald, “Fanconi anemia group C protein prevents apoptosis in hematopoietic cells through redox regulation of GSTP1,” *Nat Med*, vol. 7, no. 7, pp. 814–820, 2001, doi: 10.1038/89937.

[27]	R. S. Perkins *et al.*, “WNT5B drives osteosarcoma stemness, chemoresistance and metastasis,” *Clin Transl Med*, vol. 14, no. 5, May 2024, doi: 10.1002/ctm2.1670.

[28]	G. Housman *et al.*, “Drug Resistance in Cancer: An Overview,” *Cancers 2014, Vol. 6, Pages 1769-1792*, vol. 6, no. 3, pp. 1769–1792, Sep. 2014, doi: 10.3390/CANCERS6031769.

[29]	P. Borst, R. Evers, M. Kool, and J. Wijnholds, “A family of drug transporters: the multidrug resistance-associated proteins,” *J Natl Cancer Inst*, vol. 92, no. 16, pp. 1295–1302, Aug. 2000, doi: 10.1093/JNCI/92.16.1295.

[30]	F. M. V. Salazar and I. D. P. Arcila, “In silico study about the influence of electroporation parameters on the cellular internalization, spatial uniformity, and cytotoxic effects of chemotherapeutic drugs using the Method of Fundamental Solutions,” *Med Biol Eng Comput*, 2023, doi: 10.1007/s11517-023-02964-2.

[31]	F. M. Vélez Salazar and I. D. Patiño Arcila, “Influence of electric pulse characteristics on the cellular internalization of chemotherapeutic drugs and cell survival fraction in electroporated and vasoconstricted cancer tissues using boundary element techniques,” *J Math Biol*, vol. 87, no. 2, p. 31, Aug. 2023, doi: 10.1007/s00285-023-01963-z.

[32]	F. M. Vélez Salazar, I. D. Patiño Arcila, and C. A. Ruiz Villa, “Simulation of the influence of voltage level and pulse spacing on the efficiency, aggressiveness and uniformity of the electroporation process in tissues using meshless techniques,” *Int J Numer Method Biomed Eng*, vol. 36, no. 3, p. e3304, Mar. 2020, doi: 10.1002/cnm.3304.

[33]	F. M. Vélez Salazar, I. D. Patiño Arcila, C. A. Ruiz Villa, and A. Hernández-Blanquisett, “In-silico study about the influence of electroporation parameters on the chemotherapeutic drug transport in cancer tissues using the meshless method of approximate particular solutions,” *Computers & Mathematics with Applications*, vol. 125, pp. 116–135, Nov. 2022, doi: 10.1016/j.camwa.2022.08.034.

[34]	S. Eikenberry, “A tumor cord model for Doxorubicin delivery and dose optimization in solid tumors,” *Theor Biol Med Model*, vol. 6, no. 1, p. 16, Dec. 2009, doi: 10.1186/1742-4682-6-16.

[35]	F. Argus, B. Boyd, and S. M. Becker, “Electroporation of tissue and cells: A three-equation model of drug delivery,” *Comput Biol Med*, vol. 84, no. 1, pp. 226–234, May 2017, doi: 10.1016/j.compbiomed.2017.04.001.
