# Supplementary material for: In-silico tool based on Boolean networks and meshless simulations for prediction of reaction and transport mechanisms in the systemic administration of chemotherapeutic drugs
Source: PLoS One. 2025 Feb 7;20(2):e0315194. doi: 10.1371/journal.pone.0315194 (PMC11805580; doi:10.1371/journal.pone.0315194)
Supplement: Attachment — Submitted filename: Response letter PLOS ONE - 06_11_2024 - Last.docx [file pone.0315194.s046.docx]

Dear

Baeckkyoung Sung, Ph.D.

Academic Editor

PLOS ONE

Ref: Response letter.

In this letter, the responses to comments of reviewer 4 about the manuscript “*In-silico tool based on Boolean Networks and meshless simulations for prediction of reaction and transport mechanisms in the systemic administration of chemotherapeutic drugs”* submitted to the journal *“PLOS ONE”* are presented in detail, indicating the parts of the new manuscript where the corresponding corrections have been attended. We really acknowledge the thoughtful and comprehensive revision of the present manuscript since this allows improving its quality and clarity.

Sincerely,

Fabián Mauricio Vélez Salazar.

*Corresponding author.*

***REVIEWER 4.***

- **Comment 1:**

Model availability, your response is "If manuscript is accepted, algorithm
will be available on demand." --> This is not sufficient for progress and FAIR data policy in science, the model must become available for download as a condition for acceptance.

**Response:**

The development of the Boolean model is included into the section 3 of the original manuscript. This model was implemented in MatLab^TM^. Following the reviewer’s recommendation, the algorithms of the Boolean model have been disclosed in the supplementary material in the new version of the manuscript.

- **Comment 2:**

Fine, complement to existing heuristics, nice pharmacological example given and in the manuscript revisions.

**Response:** Comment OK.

- **Comment 3:**

Experimental data are lacking -- still the case, but data from literature were collected, so not optimal but ok.

**Response:** Comment OK.

- **Comment 4:**

Not well answered, if the practical use case was published before (ref 17) and is lacking here. Rather add your nice pharmacological model of chemotherapeutic concentrations and explain how you can protect the patient better from side-effects with your model

**Response:**

A summary of the pharmacological model was added in Section 2 of the original version of the manuscript. Specifically, the algorithm for calculating the spatiotemporal evolution of the extracellular, free intracellular and bound intracellular concentrations (C_1_, C_2_, C_3_) was presented in Figure 3. From the time integration of these concentration fields, the chemotherapeutic drug exposure, commonly referred to as AUC (Area Under the Curve), can be estimated; the AUC parameter is directly linked to the cell-kill capacity (efficacy) and cytotoxicity of the therapy.

To address the Reviewer’s comment, the following lines were included in the Section 6 of the new manuscript ***(Pages 40-41)***:

*“Generally speaking, most anticancer agents have a very narrow therapeutic window, which means that a small change in dosage can lead to unexpected side effects in the patient. This is one of the main risks of some heuristic methods widely used in clinical practice, such as BSA, which, not being closely correlated with AUC, may generate different cytotoxic processes in patients with similar characteristics (weight, age, height, health problems, etc.), such as systemic DNA degeneration and inflammation of healthy tissues, in turns leading to less severe side effects (nausea, vomiting, dysgeusia, hair loss), life-threatening effects (myelosuppression, febrile neutropenia, severe oral mucositis, sepsis, among others) or late effects at cardiac, pulmonary, neurological, renal or hepatic level. The model for the concentration fields developed and validated in previous works [1], [2], [3], which was briefly presented in Section 2 of the manuscript and was used to generate the inputs of the Boolean model, allows estimating the exposure to the chemotherapeutic drug considering the parameters of the electroporation protocol, the reaction and transport properties of the drug in the tissue, the geometrical and electrical properties of the tissue, information of the chemotherapeutic treatment (type of drug, injected dose, pharmacokinetic profile, treatment time, among others), fitting parameters of the chemotherapeutic cell death models, and characteristics related to the blood perfusion rate (vasoconstriction, blood velocity and pressure, viscosity, etc.). Accordingly, under the premise of the high correlation between the drug exposure and side effects of the therapy, this model could be very useful to complement the design of chemotherapeutic protocols by heuristic methods, focused on reducing the side effects without considerably compromising the efficacy of the treatment. This could be addressed in detail in future works”.*

- **Comment 5:**

Fine.
